# Acidity Reversal Enables Site-Specific Ring-Opening Polymerization of Epoxides from Biprotonic Compounds

**DOI:** 10.1021/jacs.4c15676

**Published:** 2025-01-22

**Authors:** Urška Češarek, Lijun Liu, Qiyi Chen, Tianyuan Wen, Ema Žagar, Junpeng Zhao, David Pahovnik

**Affiliations:** † Department of Polymer Chemistry and Technology, National Institute of Chemistry, Hajdrihova 19, 1000 Ljubljana, Slovenia; ‡ Faculty of Chemistry and Chemical Technology, University of Ljubljana, Večna Pot 113, 1000 Ljubljana, Slovenia; § Faculty of Materials Science and Engineering, South China University of Technology, Guangzhou 510640, China; ∥ Guangdong Provincial Key Laboratory of Luminescence from Molecular Aggregates, South China University of Technology, Guangzhou 510640, China

## Abstract

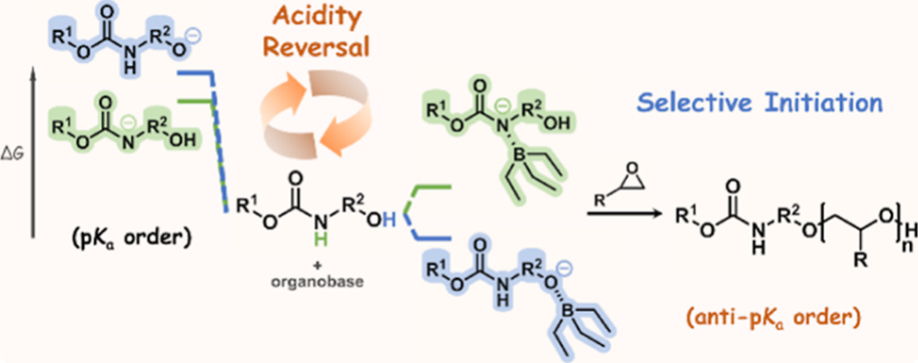

Polyethers are versatile materials extensively used in advanced as well as everyday applications. The incorporation of primary amine functionality into polyethers is particularly attractive due to its well-established coupling chemistries. However, the inherent nucleophilicity of amine group poses a challenge in the anionic ring-opening polymerization (ROP) of epoxides and requires the use of robust protecting groups that can withstand the harsh conditions of ROP without triggering undesirable side reactions. In this work, we present streamlined synthesis of amino-functionalized polyethers using classic *N*-carbamate-protected aminoalcohols as initiators for the ROP of epoxides. A Lewis acid-excess two-component organocatalytic system is found to trigger efficient anionic ROP of epoxides while preserving the integrity of the carbamate protection. Despite the higher intrinsic acidity of the carbamate group compared to the hydroxyl group, it is noncompetitive in both the deprotonation and ring-opening steps. This is due to an intriguing acidity-reversing effect of the catalyst, which allows site-specific ethoxylation to proceed exclusively from the hydroxyl group. The resulting poly(propylene oxide) and poly(ethylene oxide) exhibit the targeted molar mass, low dispersity, and well-defined end groups. The fidelity of the amino functionalities is further corroborated and utilized in construction of polypeptoide-based hybrid block copolymers using the synthesized polyethers as macroinitiators.

## Introduction

Aliphatic polyethers derived from epoxides, in particular ethylene oxide (EO) and propylene oxide (PO), are massively produced and widely studied as building blocks for a large variety of industrial products and cutting-edge materials.^1,2^ Poly(ethylene oxide) (PEO) is of particular interests for pharmaceutical applications due to its water solubility and biocompatibility. Among others, it is used for the covalent conjugation of various biopharmaceuticals, such as proteins and oligonucleotides, in order to improve their pharmacokinetic properties.^3^ In addition to the narrow molar mass distribution of PEO and the reactive end group required for conjugation, the advanced applications often demand branched polymer architecture,^4^ which can lead to challenges in ensuring the suitable end group fidelity. Traditional methods for polyether synthesis are based on the use of water/alcohols as initiators in combination with alkali metal compounds as catalysts.^2^ Among the polyether end group functionalities, the primary amino group is highly desirable due to its well-established coupling chemistry. However, due to its nucleophilicity, it has to be protected accordingly, otherwise it can easily initiate ring-opening polymerization (ROP) of epoxides.^5^ Two types of initiators are commonly used for the preparation of α-amino-functionalized polyethers; (i) alcohols with a functionality that can be modified after polymerization to α-amino-functionalized polyethers, such as propargyl alcohol,^6,7^ allyl alcohol,^8^ and α-methylbenzyl cyanide,^9,10^ and (ii) *N*-protected aminoalcohols in the forms of disilylamine,^11,12^ dibenzylamine,^13,14^ benzylideneamine,^15^ and triazine ring.^16^ In the latter case, it is important that the amino-protecting groups are robust enough to withstand the harsh anionic ROP conditions without undergoing any side reactions.

Recently, numerous alternative catalytic systems, including a considerable portion of metal-free catalysts, have been developed for the ROP of epoxides, e.g. phosphazene bases^17,18^ and *N*-heterocyclic carbenes,^6^ as well as their combinations with Lewis acids in binary catalytic systems.^19−23^ The addition of Lewis acids, which are usually considered to activate the epoxy ring for ROP, enables the transition from the use of very strong bases as catalysts to much milder bases, thereby mitigating some undesirable side reactions such as chain transfer to monomer during poly(propylene oxide) (PPO) synthesis.^24^ In addition, these catalytic systems expand the scope of initiators to nonhydroxy compounds such as carboxylic acids^25^ and secondary amides,^26^ which facilitates the preparation of α,ω-heterofunctional polyethers. The selectivity of such catalytic systems for ROP is further demonstrated by using hydroxycarboxylic esters as initiators, where transesterification, which is much faster compared to propagation in traditional anionic ROP,^27^ is completely suppressed, opening up the possibility of incorporating carbonyl-based groups in the initiator structures.^28^ However, the classic carbamate-based amino-protecting groups such as *tert*-butyloxycarbonyl (Boc) and benzyloxycarbonyl (Cbz) have not yet been considered as suitable protecting groups for use in the ROP of epoxides due to their relatively high acidity and consequently the competitiveness in deprotonation/activation in comparison with hydroxyl groups, as shown by Illy et al., who have used carbamate as an initiator with triisobutylaluminum Lewis acid and phosphazene base for the ROP of butylene oxide.^29^ Recently, triethylborane (Et_3_B) has been used as a Lewis acid in combination with a phosphazene base ^*t*^BuP_1_ for the preparation of α-amino-ω-hydroxyl-PEO in one step, where Et_3_B acts as a noncovalent protecting group that can be easily removed by precipitation in diethyl ether.^30^ However, such lability of the protecting group can be problematic if only one of the end groups is to be selectively reacted in the next step.

In this work, we present the facile synthesis of amino-functionalized polyethers using *N*-carbamate-protected aminoalcohols as initiators. We show that the ^*t*^BuP_2_-Et_3_B catalytic system tolerates the carbamate group in the structure of the initiator and ensures chemoselective initiation from the hydroxyl group by exerting a unique acidity reversal effect. This synthetic approach further enables the selective modification of both polyether chain ends and facilitates the preparation of polyether-polypept(o)ide hybrid block copolymers, particularly of complex, nonlinear architecture.

## Results and Discussion

**Scheme 1:**
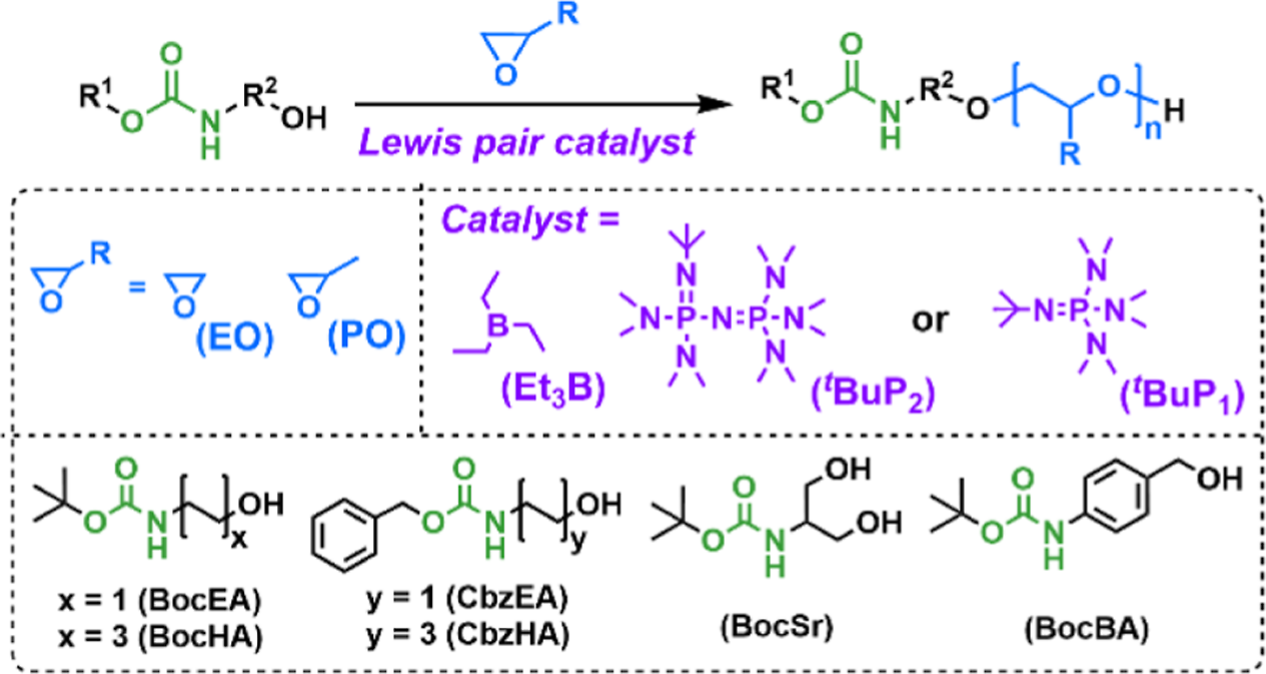
ROP of Epoxides Initiated by *N*-Carbamate-Protected Aminoalcohols

The wide variety and easy accessibility of *N*-carbamate-protected aminoalcohols makes them ideal for the introduction of structurally diverse amino functional groups into polyether chain end. In order to selectively initiate the ROP of epoxides from the hydroxyl group of a *N*-carbamate-protected aminoalcohol and perform polymerization in a controlled manner, two major challenges must be overcome: (i) preventing side reactions due to the high nucleophilicity of the growing chain end and the electrophilicity of the carbonyl group in the carbamate protecting group and (ii) preventing initiation by the carbamate group which in biprotonic *N*-carbamate-protected aminoalcohols is inherently more acidic than the hydroxyl group, meaning that any alkoxide formed is expected to be rapidly protonated by the carbamate group, leading to the formation of a carbamate anion.^31^ To overcome these challenges, we used a combination of a phosphazene base, ^*t*^BuP_2_ or ^*t*^BuP_1_, and Lewis acid, Et_3_B, as a two-component organocatalytic system for the ROP of epoxides and various *N*-carbamate-protected aminoalcohols as initiators (Scheme 1).

**Figure 1:**
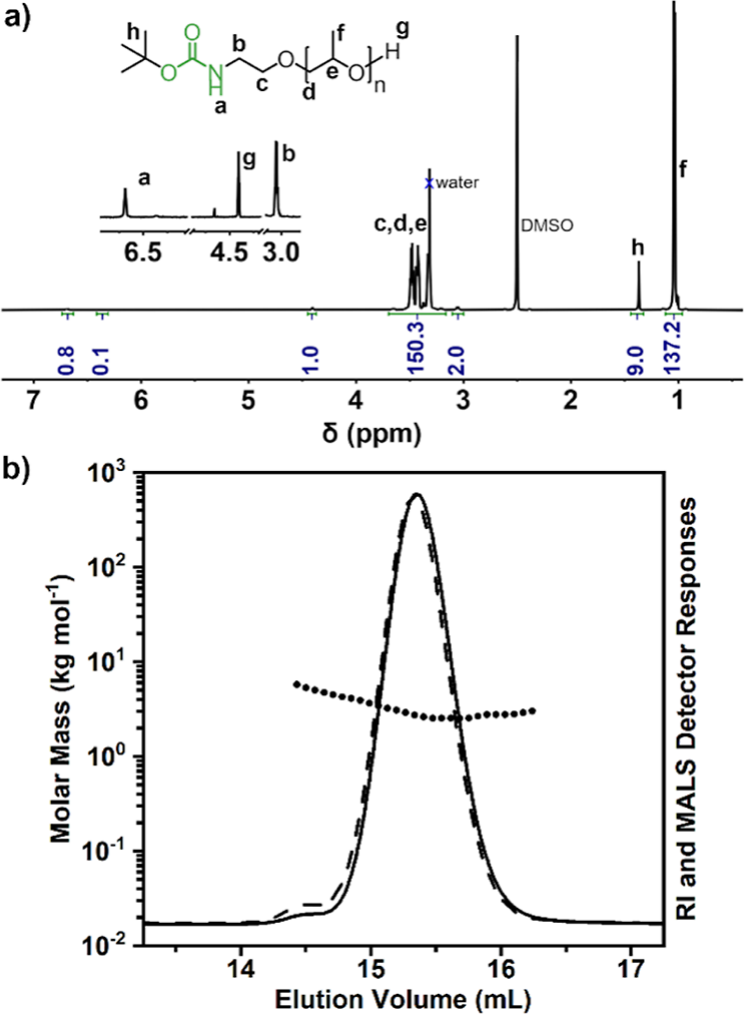
(a) ^1^H NMR spectrum and (b) SEC-MALS chromatogram of PPO initiated by BocEA (BocEA-PPO) (Table 1, entry 1).

Initially, *N*-Boc-ethanolamine (BocEA) was used as initiator for the ROP of PO (Scheme 1 and Table 1, entry 1). The ROP was performed in THF with an initiator/^*t*^BuP_2_/Et_3_B mole ratio of 1/0.025/0.1 ([PO]_0_ = 7 M). At least 4 equiv of Et_3_B per 1 equiv of phosphazene base had to be used, as the polymerization did not proceed or proceeded much slower with lower Et_3_B equiv. The reaction was carried out for 17 h until complete conversion of the monomer was confirmed by ^1^H NMR. The polymerization time of PO can be shortened to a few hours by performing reaction in bulk instead without affecting the structure of the obtained PPO (Table S1, entry 1). After quenching the reaction with acetic acid, the liquid PPO was isolated by first removing THF under reduced pressure and exchanging it with chloroform, so that water could be used for purification by extraction. The ^1^H NMR spectrum of the final product shows the typical signals of PPO with BocEA (1.38 ppm (C*H*_3_)_3_–; 3.06 ppm, −NHC*H*_2_CH_2_O–; 6.70 ppm −OCON*H*−) and hydroxyl (4.42 ppm, −CH(CH_3_)O*H*) end groups. The proton signals of ^*t*^BuP_2_ and Et_3_B are absent, indicating efficient removal of the catalytic system by extraction. The number-average molar mass (*M*_n,NMR_) of PPO, determined from the integral ratio of the signals for the PPO methyl group in the main chain (1.04 ppm, −CH_2_CH(C*H*_3_)−) and the initiator methylene group (3.06 ppm, −NHC*H*_2_CH_2_O−), agrees well with the target molar mass (Table 1, entry 1). The size exclusion chromatography (SEC) chromatogram of PPO shows a monomodal peak with a narrow molar mass distribution (*Đ* = 1.07) (Figure 1) and a trace of water-initiated PPO chains, which have twice the molar mass because water is a difunctional initiator.

**Table 1:** ROP of Epoxides from Carbamate-Containing Hydroxy Initiators^a^

entry	init.	M	[M]_0_/[init.]_0_/[^*t*^BuP_2_]/[Et_3_B]^b^	time (h)	*M*_n,th_^c^ (kg mol^–1^)	*M*_n,NMR_^d^ (kg mol^–1^)	*M*_w,SEC-MALS_^e^ (kg mol^–1^)	*Đ*^f^
1	BocEA	PO	45/1/0.025/0.1	17	2.8	2.8	2.9	1.07
2	BocEA^g^^,^^h^		45/1/0.025/0.1	4d	2.8	2.1	2.0	1.06
3	BocEA		86/1/0.05/0.2	17	5.2	5.4	5.0	1.17
4	BocHA		45/1/0.025/0.1	17	2.8	2.4	2.5	1.06
5	CbzEA		45/1/0.025/0.1	17	2.8	3.0	3.1	1.07
6	CbzEA		86/1/0.05/0.2	17	5.2	5.1	5.3	1.18
7	FmocEA		45/1/0.025/0.1		2.9			
8	BocSr		45/1/0.05/0.2	17	2.8	3.0	3.0	1.08
9	BocSr		86/1/0.05/0.2	17	5.2	5.4	5.1	1.22
10	BocEA	EO	45/1/0.025/0.1	3	2.0	2.1	2.0	1.03
11	BocEA^g^		110/1/0.025/0.1	17	5.0	5.9	5.5	1.07
12	BocEA		180/1/0.025/0.1	5	8.1	7.9	7.1	1.15
13	BocEA		460/1/0.05/0.2	5	20.4	21.6	20.5	1.10
14	BocHA		110/1/0.025/0.1	3	5.1	6.4	5.8	1.08
15	BocBA		110/1/0.025/0.1	3	5.1	5.4	5.1	1.07
16	CbzHA		110/1/0.025/0.1	2.5	5.1	5.5	5.3	1.07
17	CbzHA^g^		110/1/0.025/0.1	17	5.1	5.2	4.9	1.06
18	BocSr		110/1/0.025/0.1	3	5.0	4.9	4.6	1.06

a Performed at room temperature (*ca* 25 °C) in THF with [PO]_0_ or [EO]_0_ = 7 M, except for entry 13 where [EO]_0_ = 15 M.

b Mole feed ratio of monomer, initiator,^*t*^BuP_2_, and Et_3_B.

c Theoretical number-average molar mass calculated from the mole ratio of monomer to initiator in the feed.

d Calculated from the integral ratio of the signals of the main chain and the initiator protons of the isolated products.

e Weight-average molar mass determined by SEC–MALS.

f Dispersity determined by SEC and column calibration with PEO standards.

g ^*t*^BuP_1_ used as basic catalytic component.

h 82% conversion of the monomer was achieved.

**Figure 2:**
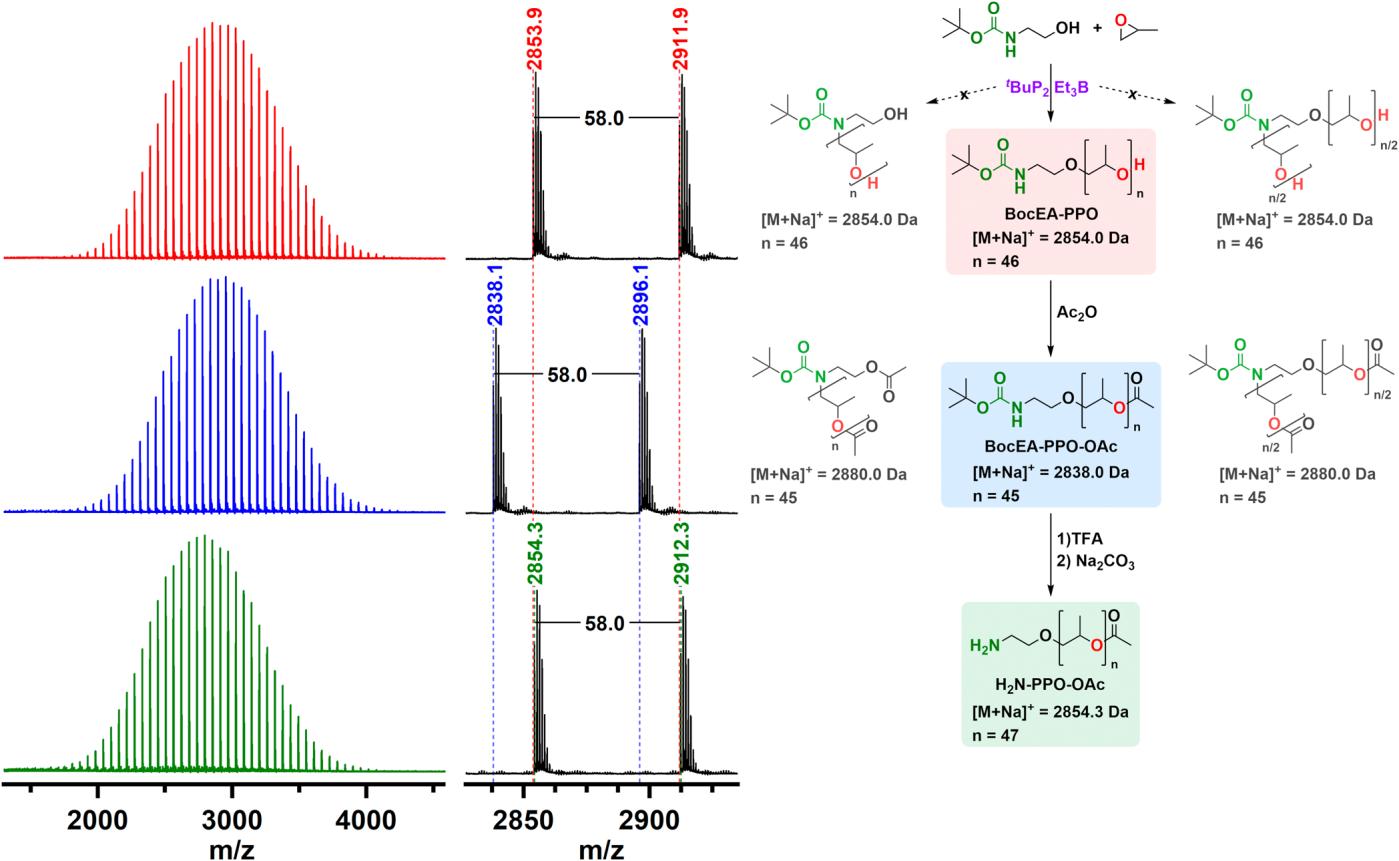
MALDI–TOF mass spectra of BocEA-PPO (Table 1, entry 1), after acetylation (BocEA-PPO-OAc), and after deprotection (H_2_N-PPO-OAc). Possible products obtained, depending on which of the two protonic species acts as initiator, with calculated exact masses, ionized with the sodium ion. The measured monoisotopic signals are indicated in the enlarged regions of the mass spectra.

Matrix-assisted laser desorption/ionization time-of-flight (MALDI–TOF) mass spectrum of BocEA-initiated PPO shows the expected molecular mass distribution (Figure 2). If the ROP of PO was initiated by the carbamate group alone or simultaneously by the carbamate and hydroxyl groups of BocEA and not selectively by the hydroxyl group, polyethers with similar molecular mass characteristics would be expected as long as initiation is not slower than propagation. Such products would also have exactly the same molecular mass distribution in the MALDI–TOF mass spectra, as they are structural isomers. However, the PPO chains that are selectively initiated by the hydroxyl group have only one hydroxyl end group, whereas the PPO chains in the other two cases would have two hydroxyl end groups in the structure (Figure 2). To further confirm that the initiation was selective from the hydroxyl group and not from the carbamate group, the BocEA–PPO sample was acetylated with acetic anhydride (Figure 2). The complete conversion of the hydroxyl group is verified by ^1^H NMR (Figure S34), where the proton signal of the hydroxyl group disappears and the methine group of the terminal PO repeating unit appears next to an ester group. The monoacetylation of the product is confirmed by the integral ratio of the signals at 1.97 and 1.38 ppm, corresponding to the ω-acetyl end group and the Boc protecting group, respectively. The end group fidelity was also confirmed by MALDI-TOF MS (Figures 2 and S35), as the structure of the obtained PPO corresponds to the monoacetylated product, while the peak distribution, which would indicate the presence of the doubly acetylated product, is absent. Finally, the Boc protecting group was selectively removed with either trifluoroacetic acid in chloroform or HCl in dioxane to obtain amino-functionalized PPO, leaving the ester group intact. In most of the MALDI–TOF mass spectra of the Boc-protected products, an additional peak distribution (−46.9 Da from the main distributions) of very low intensity was observed, corresponding to the in-source fragmentation of the Boc protecting group during MALDI–TOF MS analyses, as indicated by its disappearance after deprotection.

BocEA was then used as an initiator for the ROP of EO (Table 1, entries 10, 12 and 13). The polymerization was performed in THF under the same conditions as for PO (mole ratio initiator/^*t*^BuP_2_/Et_3_B = 1/0.025/0.1 and [EO]_0_ = 7 M), or with catalyst-to-initiator ratio and [EO]_0_ increased for higher targeted molar mass. Due to the higher reactivity of EO compared to PO, the complete conversion of EO was achieved in 3–5 h, even with lower catalyst-to-monomer ratios. The structures of the as-formed BocEA–PEOs with the expected end groups and narrow molar mass distributions were confirmed by ^1^H NMR, MALDI–TOF MS and SEC–MALS (Figures S16, S17 and S20–S23), indicating that this synthetic method is also suitable for the preparation of carbamate-protected amino-functionalized PEOs. In this case, also the weaker ^*t*^BuP_1_ can be used instead of ^*t*^BuP_2_ without affecting the structural characteristics of the obtained PEO (Table 1, entry 11 and Figures S18 and S19), only the rate of polymerization somewhat slows down (17 h is needed to achieve complete monomer conversion). Similar decrease in the polymerization rate was observed in the case of PO, where it took several days to achieve complete conversion (Table 1, entry 2).

In the case of ethanolamine-based initiators, there is a possibility that the starting anionic species form 5-membered cyclic structures through van der Waals interactions or weak intramolecular hydrogen bonds (Figure S57 and Table S2). Complexation of the *O*- or *N*-anionic species with Et_3_B increases the steric effect and coordination number, resulting in weakening of the hydrogen bonds which finally has no significant effect on epoxide ring opening. This is consistent with the results obtained with aminoalcohol initiators with longer/larger spacers, such as *N*-Boc-6-aminohexanol (BocHA) (Table 1, entries 4 and 14), *N*-Boc-4-aminobenzylalcohol (BocBA) (entry 15) and *N*-Boc-serinol (BocSr) (entries 8, 9, and 18) as they produce well-defined polyether products within comparable reaction time, suggesting that the structure of the aminoalcohol does not have a significant impact on the efficiency and selectivity of the ROP. To further demonstrate the compatibility of the catalytic system with carbamate protecting groups, Cbz-protected aminoalcohols (*N*-Cbz-ethanolamine (CbzEA) and *N*-Cbz-6-amino-1-hexanol (CbzHA)) were used to initiate the ROP of PO and EO (Table 1, entries 5–6 and 16–17). Under the same experimental conditions as for the BocEA initiator, CbzEA and CbzHA gave similar results, i.e. well-defined polyethers with controlled molar mass, low *Đ*, and complete chain-end fidelity (Figures S8–S11 and S28–S31). The selectivity of initiation by the hydroxyl group was also confirmed by ^1^H NMR and MALDI–TOF MS in combination with the acetylation strategy (Figures S38 and 39). In contrast, no polymerization was observed when *N*-fluorenylmethoxycarbonyl-protected ethanolamine (FmocEA) (Table 1, entry 7) was used as initiator, as expected, since the Fmoc protecting group can be easily removed even by weak bases such as the commonly used piperidine^32^ and is therefore incompatible with our catalytic system.

When Boc protected amines such as *N*-*tert*-butyl-methylcarbamate (BocMC) or *N*-*tert*-butyl-phenylcarbamate (BocPC) without hydroxyl groups in the structure were used as initiators (Table S1, entries 5–6), the ROP of EO proceeded rapidly, but the products have high molar masses and broad molar mass distributions, indicating that most likely impurities such as traces of water in the reaction mixtures and/or a small amount of Boc-protected amines initiated the ROP. This hence provides another evidence for the poor effectiveness of carbamate groups for reacting with the epoxide and initiating the ROP. To understand the influence of the carbamate group on the polymerization kinetics, ROP of PO in bulk was performed using either BocEA, methanol, or a methanol-BocMC mixture, as initiator (Table S1, entries 1–3). Under the same reaction conditions, the ROP of PO was only slightly slower when performed with the carbamate-containing initiator compared to the methanol initiator (Figure S54), suggesting that the carbamate group has an insignificant effect on the polymerization efficiency. In the case of ROP of PO initiated by a methanol-BocMC mixture, only PPO chains initiated by methanol were formed, as shown by MALDI–TOF MS (Figure S56). Completely selective ROP initiation of PO from methanol was also achieved with a mixture of methanol and ethyl *N*-methylcarbamate as one of the simplest carbamates, indicating that steric hindrance due to the bulkiness of the Boc and Cbz groups is not the reason for the selective initiation.

**Figure 3:**
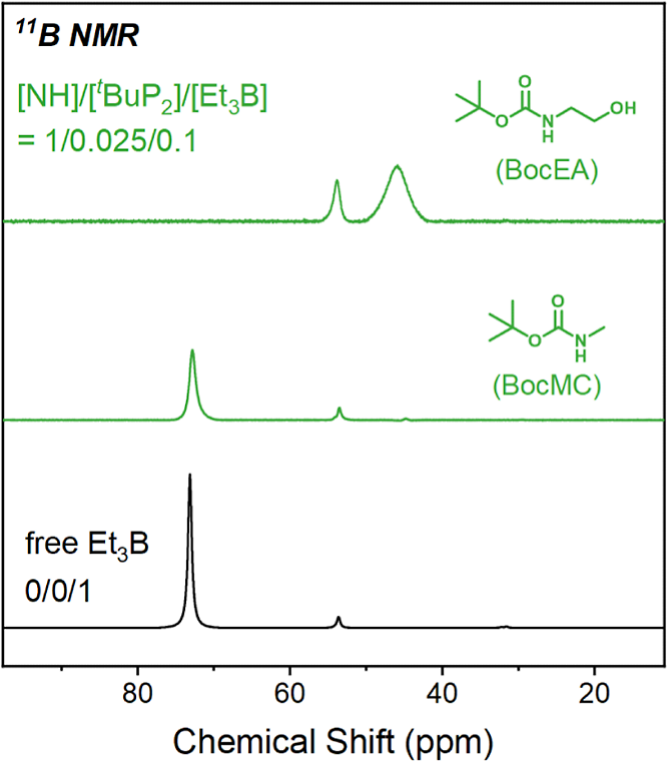
^11^B NMR spectra of Et_3_B mixed with ^*t*^BuP_2_ and carbamate (NHCOO) species compared to Et_3_B alone.

To shed the first light on the reason for the great disparity exhibited by carbamate and hydroxyl groups toward the reaction with epoxide, their interactions with the catalysts are investigated by ^11^B NMR. Carbamates with (BocEA) and without (BocMC) hydroxyl group were mixed with ^*t*^BuP_2_ and Et_3_B at the same catalyst-to-initiator ratio as in the ROP experiments to record ^11^B NMR spectra (Figure 3). In the case of BocMC, the boron signal in the ^11^B NMR spectrum almost does not shift, indicating a negligible interaction between Et_3_B and the ^*t*^BuP_2_-deprotonated carbamate group, while a significant shift to the upper field is observed for BocEA, confirming the strong and selective interaction of Et_3_B with the ^*t*^BuP_2_-deprotonated hydroxyl group.

**Figure 4:**
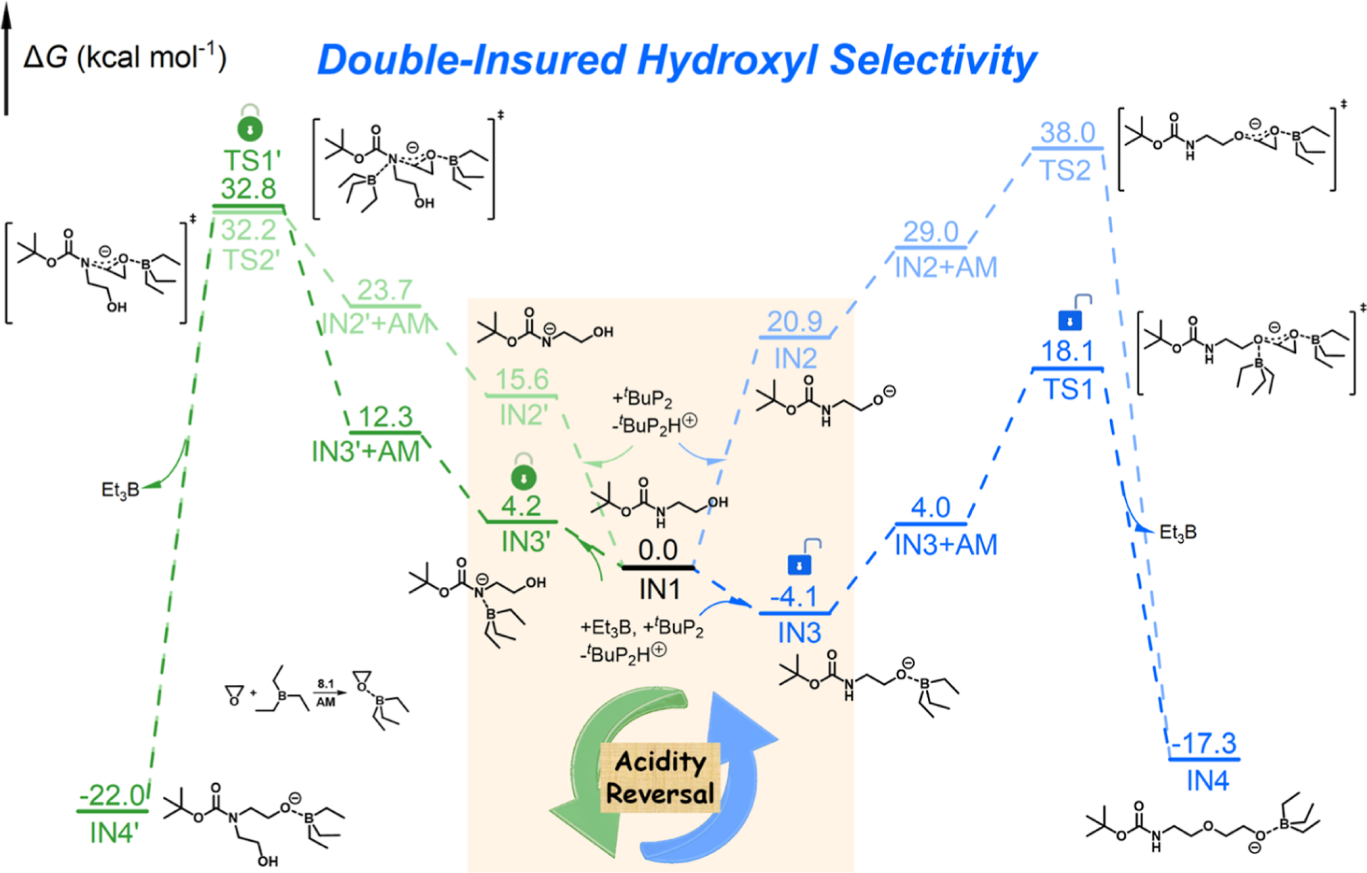
DFT-calculated Δ*G* of the intermediates and transition states for the comparison between hydroxyl-initiated (blue) and carbamate-initiated (green) ROP of EO with or without Et_3_B involved in deprotonation. Δ*G* values (kcal mol^–1^) are given above the solid lines.

Density functional theory (DFT) calculations were conducted to acquire mechanistic understanding of the hydroxyl group selectivity. EO and BocEA (**IN1**) were used as model epoxide monomer and biprotonic initiator, respectively. The Gibbs free energy (Δ*G*) values for the deprotonation of the hydroxyl group (formation of **IN2**) and the carbamate group (formation of **IN2′**) by ^*t*^BuP_2_ alone are 20.9 and 15.6 kcal mol^–1^, respectively, which is consistent with their p*K*a values^33^ and confirms that the carbamate group is inherently more acidic than the hydroxyl group (Figure 4). Nevertheless, the hydroxyl group is more easily deprotonated in the presence of Et_3_B due to the strong *O*–*B* interaction, which facilitates the formation of a more stable ternary ionic complex with ^*t*^BuP_2_ and Et_3_B (**IN3**; Δ*G* = −4.1 kcal mol^–1^) as compared with the complex formed by the deprotonated carbamate (**IN3′**; Δ*G* = 4.2 kcal mol^–1^). This acidity reversal effect thus serves as the first insurance for the site-specific activation of the hydroxyl group. **IN3** subsequently reacts with the activated monomer (**AM**) to produce **IN4**, with one EO unit added to the hydroxyl group, surmounting an energy barrier of 22.2 kcal mol^–1^ (Δ*G*_TS1_^⧧^, Figure S58). The nucleophilic attack of the **AM** by **IN3′** to add one EO unit to the carbamate group encounters a significantly higher energy barrier of 32.8 kcal mol^–1^ (Δ*G*_TS1′_^⧧^, Figure S59). Such a difference provides the second insurance for the site-specific ethoxylation of the hydroxyl group. On the other hand, both of the two “naked” anionic species (**IN2** and **IN2′**) need to overcome substantially high energy barriers of 38.0 and 32.2 kcal mol^–1^ to react with **AM**, thus ruling out these routes. Replacing the primary hydroxyl group with a secondary one (modeling the end group of PPO), or replacing the (protected) aliphatic amino group with an aromatic one (in BocBA), is not found to change the rule of acidity reversal or site-specific ethoxylation (Figures S60 and S61).

**Figure 5:**
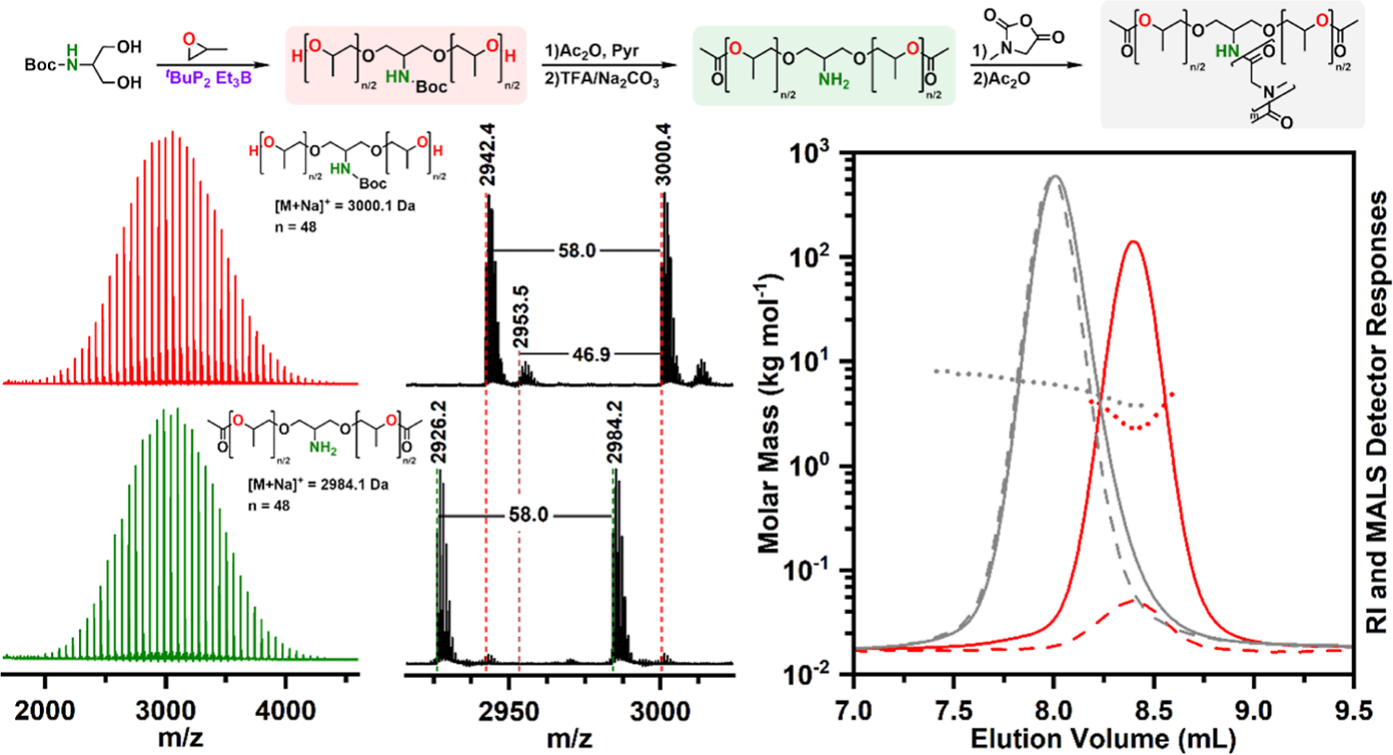
Synthesis of PSar-*b*-(PPO)_2_ AB_2_ miktoarm star from BocSr-(PPO)_2_ (Table 1, entry 8). MALDI–TOF mass spectra of PPO initiated by BocSr (BocSr-(PPO)_2_) after acetylation and deprotection (H_2_N-(PPO-OAc)_2_). SEC–MALS chromatograms of BocSr-(PPO)_2_ (red) and PSar-*b*-(PPO)_2_ AB_2_ miktoarm star (gray).

A straightforward pathway for the preparation of α-protected amino-ω-hydroxyl polyethers is of clear advantage as it allows the selective modification of the hydroxyl chain end prior to the removal of the carbamate protecting group to obtain the amino end-functionalized polyether chain. In addition to acetylation with acetic anhydride, the hydroxyl end group can also be readily esterified with carboxylic acids using coupling reagents. The coupling of *N*-Boc-glycine using 1-ethyl-3-(3-dimethylaminopropyl)carbodiimide hydrochloride to CbzEA-PPO allowed us to prepare orthogonally protected α,ω-amino-PPO (Scheme S1). The complete conversion of the hydroxyl groups was confirmed by ^1^H NMR (shifting of the terminal methine group and absence of the proton signal of the hydroxyl group) and MALDI–TOF MS (Figures S42 and S43). Both amino groups can be selectively deprotected, which would simplify the preparation of linear ABC triblock copolymers.^34^ This is particularly important for the preparation of hybrid block copolymers based on polypept(o)ides via ROP of amino acid *N*-carboxyanhydrides. While the developed synthetic approach to α-protected amino-ω-hydroxyl polyethers proves useful for the preparation of linear block copolymers, it is essential for the preparation of more complex polymer architectures such as miktoarm stars from a heterofunctional core. In this case, the coexistence of different functional groups is unavoidable and several additional steps were usually required to prepare the suitable macroinitiator. In our approach, a well-defined trifunctional PPO with a carbamate-protected amino group between the two PPO chains and a hydroxyl end group at both PPO chain ends is prepared by using BocSr as initiator (Table 1, entry 8 and Figure 5). After acetylation of the hydroxyl group and removal of the Boc protecting group, the obtained amino-functionalized macroinitiator H_2_N-(PPO-OAc)_2_ was successfully used to prepare well-defined amphiphilic PSar-*b*-(PPO)_2_ AB_2_ miktoarm star copolymers (Table S3 and Figure 5 and S63).

## Conclusions

In summary, we have shown that the classic carbamate protecting groups remain intact during ROP of epoxides when ^*t*^BuP_1_/^*t*^BuP_2_ and Et_3_B are used as the organocatalytic system. The carbamate group, despite its high intrinsic acidity, is noncompetitive as a precursor nucleophile in both deprotonation and ring-opening processes, and thus allows site-specific ethoxylation (initiation and propagation) from the hydroxyl group of biprotonic compounds, i.e. *N*-carbamate-protected aminoalcohols. The reversal of relative acidity, enabled by the much stronger *O*–*B* interaction compared to the *N*–*B* interaction, has been shown to be a key mechanism for selectivity between the two coexisting protonic groups. This synthetic approach, utilizing robust covalent amino-protection, facilitates the selective modification of both α- and ω-chain ends. The high fidelity of amino functionality allows the polyethers to be used as macroinitiators for the preparation of complex polyether-polypept(o)ide hybrid block copolymers, such as AB_2_ miktoarm stars, with high precision. This approach considerably expedites the synthesis of amino-functionalized polyethers that can be used as versatile building blocks for the design and construction of polyether-polypeptide hybrid block copolymers, poly(urea-urethane)s, polyether-protein conjugates, etc. with tailored properties and expandable (biomedical) applications.

## Supporting Information Available

Experimental procedures and characterization data for products including ^1^H NMR spectra, MALDI–TOF mass spectra and SEC–MALS chromatograms, and summary of DFT computational studies (PDF)
