# Supplementary material for: Acidity Reversal Enables Site-Specific Ring-Opening Polymerization of Epoxides from Biprotonic Compounds
Source: J Am Chem Soc. 2025 Jan 22;147(6):5189–96. doi: 10.1021/jacs.4c15676 (PMC11826984; doi:10.1021/jacs.4c15676)
Supplement: Supplementary file 1 — ja4c15676_si_001.pdf [file ja4c15676_si_001.pdf]

Supporting Information for

Acidity Reversal Enables Site-Specific Ring-Opening Polymerization of Epoxides from Biprotonic Compounds

Urška Češarek,[a,b,#] Lijun Liu,[c,#] Qiyi Chen,[c] Tianyuan Wen,[c] Ema Žagar,[a] Junpeng Zhao,[c,d,*] David Pahovnik[a,*]

[a] Department of Polymer Chemistry and Technology, National Institute of Chemistry, Hajdrihova 19, 1000 Ljubljana, Slovenia
[b] Faculty of Chemistry and Chemical Technology, University of Ljubljana, Večna pot 113, 1000 Ljubljana, Slovenia
[c] Faculty of Materials Science and Engineering, South China University of Technology, Guangzhou 510640, China
[d] Guangdong Provincial Key Laboratory of Luminescence from Molecular Aggregates, South China University of Technology, Guangzhou 510640, China

[#] These authors contributed equally.
[*] Address correspondence to david.pahovnik@ki.si and msjpzhao@scut.edu.cn

Table of Contents

S1. Experimental S2
S2. Characterization data of *N*-protected-amino-polyethers S6
S3. End-group modification and characterization S22
S4. Mechanistic studies S33
S5. Block copolymers preparation and characterization S38
S6. Computational Details S40
S7. Reference S65

S1. Experimental

Materials

Ethylene oxide (EO; Aldrich, 99%) was condensed from a steel cylinder into a Schlenk flask prefilled with sodium hydride (NaH) by cooling the flask to 0 °C under vacuum and stirred with NaH in an ice water bath for 4 h. Then, EO was cryo-condensed into a graduated cylindrical flask with *n*-butyllithium (^{n}BuLi) and finally into the reaction flask. *EO is volatile, potentially explosive and toxic if inhaled. Great care must be taken when handling EO.* Propylene oxide (PO; Sigma-Aldrich, 99%) was stirred with calcium hydride (CaH_2) at room temperature before it was finally cryo-condensed into the reaction flask. Tetrahydrofuran (THF; Shanghai Titan Scientific Co., Ltd., AR; anhydrous, Sigma-Aldrich ≥99.9%) was successively dried with CaH_2 and ^{n}BuLi before being cryo-distilled. *N*-Boc-ethanolamine (BocEA; Sigma-Aldrich, 98%), *N*-Cbz-ethanolamine (CbzEA; Sigma-Aldrich, 98%), *N*-Fmoc-ethanolamine (FmocEA; Sigma-Aldrich, 97%), *N*-*tert*-butyl-methylcarbamate (BocMC; Energy Chemical, >98%), *N*-*tert*-butyl-phenylcarbamate (BocPC; Energy Chemical, >97%), *N*-Boc-6-aminohexanol (BocHA; Merck, ≥98%), *N*-Boc-4-aminobenzylalcohol (BocBA; Energy Chemical, >98%), *N*-Cbz-6-amino-1-hexanol (CbzHA; Energy Chemical, >97%) and *N*-Boc-serinol (BocSr; Sigma-Aldrich, 97%) were dried by azeotropic distillation of THF prior to use. Sarcosine (Sar; Iris Biotech), *tert*-butylimino-tris(dimethylamino)phosphorane (^{t}BuP$_1$; Aldrich, >97%), 1-*tert*-butyl-2,2,4,4,4-pentakis(dimethylamino)-2λ^5,4λ^5-catenadi(phosphazene) (^{t}BuP$_2$; Sigma-Aldrich, 2.0 mol L^{-1} in THF), triethylborane (Et_3B, 1 M in hexane, Sigma-Aldrich), triphosgene (Aldrich, 98%), pyridine (Pyr, anhydrous, Fischer scientific), acetic anhydride (Ac_2O, Sigma-Aldrich, 99%), acetic acid (AcOH, Sigma-Aldrich, ≥99%), *N*-Boc-glycine (BocGly; Sigma-Aldrich, 99%), 4-dimethylaminopyridine (DMAP; Sigma-Aldrich, 99%), 1-ethyl-3-(3-dimethylaminopropyl)carbodiimide hydrochloride (EDC·HCl, IRIS Biotech, >98%), palladium on carbon (Pd/C; 10 wt.%, Sigma-Aldrich), trifluoroacetic acid (TFA; Sigma-Aldrich, 99%), hydrochloric acid solution (4 M solution in dioxane, Sigma-Aldrich), $MgSO_4$ (anhydrous, Sigma-Aldrich, 99.5%), Na_2HSO_4 (Fluka, 95%), Na_2CO_3 (anhydrous, Sigma-Aldrich), NaCl (Sigma-Aldrich, ≥99.5%), chloroform (anhydrous, Sigma-Aldrich, ≥99% and Sigma-Aldrich, ≥99.8%), dichloromethane (DCM; anhydrous, Sigma-Aldrich, ≥99.8% and Sigma-Aldrich, ≥99.9%), methanol (Energy Chemical, 99.8%) and *n*-hexane (Sigma-Aldrich, ≥99%) were used as received.

Instrumentation

^{1}H and ^{11}B NMR spectra of the samples were recorded on a Bruker AVANCE NEO 600 MHz or a Bruker AV500 NMR spectrometer at room temperature. The spectra were recorded either in DMSO-d_6 or $CDCl_3$ with or without a few drops of TFA for ^{1}H NMR, and in THF-d_8 for ^{11}B NMR. Tetramethylsilane (TMS, $\delta = 0$) was used as an internal chemical-shift standard. A small intensity signal in ^{11}B NMR spectra, which does not shift upon addition of the base and alcohol, corresponds to the air-oxidation of Et_3B during the measurement process.[1]

Size-exclusion chromatographic measurements coupled with a multi-angle light-scattering photometer (SEC-MALS) were performed using an HPLC system consisting of an isocratic pump, a degasser and an autosampler (Agilent Technologies, USA), and equipped with a Dawn Heleos II multi-angle light-scattering photometer (Wyatt Technology Corp., USA) with a linearly polarized GaAs laser (λ_0 = 661 nm) and an Optilab rEX interferometric refractometer (RI) (Wyatt Technology Corp., USA), which operates at the same wavelength as the photometer. Calibration of the 90° light scattering detector was performed with toluene, while normalization of the other LS detectors was performed with a PEO standard with a weight-average molar mass of 4 kg mol^{-1} and a dispersity of 1.04. The homopolymers were separated by size using two successively coupled SEC columns TSKgel Alpha-3000 (7.8 mm ID × 30.0 cm L, particle size 7 µm and exclusion limit 60 kDa) and TSKgel Alpha-2500 (7.8 mm ID × 30.0 cm L, particle size 7 µm and exclusion limit 10 kDa) with a precolumn (all Tosoh Bioscience GmbH, Germany) at room temperature. Methanol (Merck) with a flow rate of 0.7 mL min^{-1} was used as solvent and mobile phase. The block copolymers were size-separated at 50 °C using a TSKgel Alpha-3000 SEC column with a precolumn in 0.05 M LiBr in *N*,*N*-dimethylacetamide (Sigma-Aldrich) at a flow rate of 0.4 mL min^{-1}. The masses of the samples injected onto the column were typically 1.0 × 10^{-3} g, while the solution concentrations were typically 1.0 × 10^{-2} g mL^{-1}. The samples' d*n*/d*c* values, required for the calculation of the molar mass characteristics, were determined assuming 100% mass recovery of the samples from the columns. Astra 8 software (Wyatt Technology Corp., USA) was used for data acquisition and evaluation.

Matrix-assisted laser desorption/ionization time-of-flight mass spectrometry (MALDI-TOF MS) measurements were performed using a Bruker UltrafleXtreme MALDI-TOF mass spectrometer (Bruker Daltonics). Homopolymers were dissolved in THF (10 mg mL^{-1}) or reaction aliquots were diluted with THF and mixed with a solution of *trans*-2-[3-(4-*tert*-butylphenyl)-2-methyl-2-propenylidene]-malononitrile (DCTB, 20 mg mL^{-1}) or 2,5-dihydroxybenzoic acid (DHB, 30 mg mL^{-1}) as matrix and sodium trifluoroacetate (10 mg mL^{-1}) as cationizing agent in THF in a volume ratio of 1 : 10 : 3. 0.5 µL of the prepared solution was spotted onto the target plate. The reflective positive ion mode was used to acquire the mass spectra of the samples. Calibration was performed externally with a mixture of poly(methyl methacrylate) standards dissolved in THF (MALDI validation set PMMA, Fluka Analytical) covering the measured molecular weight range.

Ring-opening polymerization

ROP of PO initiated by BocEA. A typical procedure is given as follows (Table 1, entry 1). BocEA (256.0 mg, 1.59 mmol) was first added to a flame-dried Schlenk flask under argon atmosphere and dissolved in dry THF (5 mL). Purified PO (5 mL, 71.45 mmol, $[PO]_0$ = 7 M) was then slowly cryo-condensed at –20 °C. Finally, Et_3B solution (160 µL, 0.16 mmol) and ^{t}BuP_2 solution (20 µL, 0.04 mmol) were added sequentially. The reaction mixture was stirred at room temperature under vacuum. After completion of

polymerization, the reaction mixture was concentrated under reduced pressure and the crude product was diluted with chloroform and washed with water, 10% Na_2CO_3 aq., 0.1 M $NaHSO_4$ aq., and water. The organic layer was dried with $MgSO_4$ and the solvent was removed under reduced pressure to obtain a transparent liquid product (BocEA-PPO; 90% yield). Entries 2-9 in Table 1 and entries 1-4 in Table S1 were prepared by the same procedure. The ^{1}H NMR and MALDI-TOF MS spectra with signal assignments are shown in Figures 1, S1-S15 and S55-S56.

ROP of EO initiated by BocEA. A typical procedure is given as follows (Table 1, entry 10). A Schlenk flask equipped with a Teflon stopcock was dried at 110 °C for 12 h in a blast drying oven and transferred to a glovebox, where BocEA (215.1 mg, 1.34 mmol), THF (5.6 mL), Et_3B (1.0 mol L^{-1}, 133.5 μL, 0.13 mmol), and ^{t}BuP$_2$ (16.7 μL, 0.033 mmol) were added sequentially. The flask was then docked to the vacuum line and purified EO (3.0 mL, 60.1 mmol) was slowly cryo-condensed at –20 °C. The mixture was stirred at 0 °C in an ice bath, which was gradually warmed to room temperature. Within 3 h, a large amount of a white solid (crystallized PEO) appeared. The product was dissolved in DCM containing a few drops of acetic acid and precipitated in cold diethyl ether. The white powder was then collected and dried in vacuum (BocEA-PEO; >95% yield). Entries 10-18 in Table 1 and entries 5-6 in Table S1 were prepared by the same procedure. The ^{1}H NMR and MALDI-TOF MS spectra with signal assignments are shown in Figures S16-S33.

Postpolymerization modifications of *N*-protected-amino-polyethers

O-Acetylation of the hydroxyl end group. To a solution of BocEA-PPO (2.00 g, 0.71 mmol) in anhydrous chloroform (7 mL), pyridine (1.14 mL, 14.12 mmol), and DMAP (8.81 mg, 0.07 mmol) were added. Acetic anhydride (0.67 mL, 7.06 mmol) was then added dropwise on the ice bath and the reaction mixture stirred overnight at room temperature. The reaction mixture was then diluted with chloroform and washed with water, 10% Na_2CO_3 aq., 0.1 M $NaHSO_4$ aq., and water. The organic layer was dried with $MgSO_4$ and the solvent was removed under reduced pressure to obtain a transparent liquid product (BocEA-PPO-OAc; 86% yield). In the case of the PEO samples, isolation was instead performed by precipitation of the reaction mixture in diethyl ether, followed by drying of the product in vacuum. The ^{1}H NMR and MALDI-TOF MS spectra with signal assignments are shown in Figures S34-S41.

Synthesis of diamino end-functionalized PPO. To a solution of CbzEA-PPO (0.90 g, 0.30 mmol) in anhydrous DCM (6 mL), DMAP (36.2 mg, 0.30 mmol), *N*-Boc-glycine (0.13 g, 0.74 mmol), and EDC-HCl (0.17 g, 0.89 mmol) were added sequentially. The reaction was stirred overnight at room temperature. The reaction was diluted with DCM and washed with water, 10% Na_2CO_3 aq., 0.1 M $NaHSO_4$, and water. The organic layer was dried ($MgSO_4$) and the solvent was removed under reduced pressure to give a transparent liquid product (CbzEA-PPO-Boc; 93% yield). The ^{1}H NMR and MALDI-TOF MS spectra with signal assignments are shown in Figures S42 and S43.

Removal of protecting groups from *N*-protected-amino-polyethers

<u>*Boc deprotection*</u>. BocEA-PPO-OAc (0.25 g, 0.05 mmol) was dissolved in anhydrous chloroform (2.8 mL) at 0 °C. TFA (0.70 mL) was added dropwise over a period of 10 min and the resulting mixture was stirred at room temperature for about 1.5 h. Then the reaction mixture was diluted with chloroform and washed with 10% Na_2CO_3 aq. and water. The organic layer was dried ($MgSO_4$) and the solvent was removed under reduced pressure to give a transparent liquid product (H_2N-PPO-OAc; 87% yield). Alternatively, a 4 M solution of HCl in dioxane was used instead of TFA in chloroform. In the case of the PEO samples, isolation was instead performed by precipitating the reaction mixture in diethyl ether, followed by drying the product in vacuum. The ^{1}H NMR and MALDI-TOF MS spectra with signal assignation are shown in Figures S44-S51.

<u>*Cbz deprotection*</u>. To a solution of CbzEA-PPO-OAc (0,15 g, 0.09 mmol) in anhydrous DCM (2.00 mL) and acetic acid (0.30 mL), Pd/C (10 wt.%, 80.0 mg) was added. The apparatus for catalytic hydrogenolysis was flushed with H_2 (2.2 bar) three times and then the reaction mixture was shaken at room temperature for approximately 2 h. The catalyst was then filtered through a Celite pad, washed with 10% Na_2CO_3 aq., and water. The organic layer was dried with $MgSO_4$, and the solvent was removed under reduced pressure to give a transparent liquid product (H_2N-PPO-OAc; 79% yield). The ^{1}H NMR and MALDI-TOF MS spectra with signal assignments are shown in Figures S52 and S53.

Synthesis of sarcosine *N*-carboxyanhydride (Sar NCA)

Sar (2.00 g, 22.4 mmol) was suspended in dry THF (40 mL) in a flame-dried flask purged with argon. A solution of triphosgene (2.66 g, 8.98 mmol) in dry THF (10 mL) was then added slowly. The reaction mixture was stirred and heated in an oil bath at 65 °C for 3 h to obtain a clear solution. The reaction mixture was filtered under vacuum. The solvent was removed under reduced pressure to obtain a solid brownish product. The crude product was dissolved in 30 mL THF. Then, 15 mL of *n*-hexane was slowly added and the reaction mixture was kept at -18 °C overnight. The white crystals obtained were filtered under argon and dried under reduced pressure. The crude product was sublimated at 90 °C under high vacuum for 2 h in the sublimation apparatus (62% yield). The ^{1}H NMR spectrum with signal assignments is shown in Figure S62.

Synthesis of PSar-*b*-$(PPO)_2$ block copolymer

Sar NCA (140.0 mg, 1.22 mmol) was suspended in anhydrous chloroform (2 mL), followed by the addition of a solution of H_2N-$(PPO\text{-}OAc)_2$ (1.90 mL, 0.06 mmol, 32 mM) in anhydrous chloroform, dried over 4 Å molecular sieves. The reaction mixture was stirred overnight at room temperature. The solvent was then removed under reduced pressure to give a transparent, waxy product (89% yield). The ^{1}H NMR spectrum with signal assignments is shown in Figures S63.

S2. Characterization data of *N*-protected-amino-polyethers

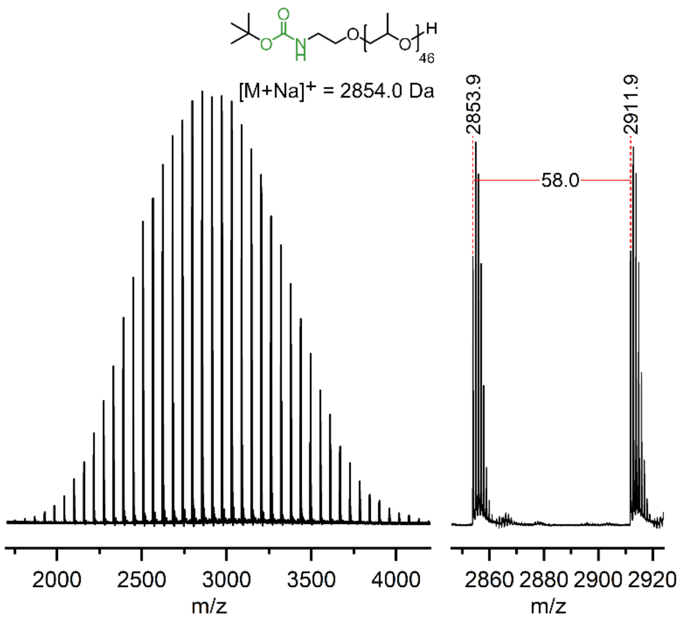

Figure S1. MALDI-TOF mass spectrum and its enlarged region with denoted measured monoisotopic signals for BocEA-PPO (Table 1, entry 1).

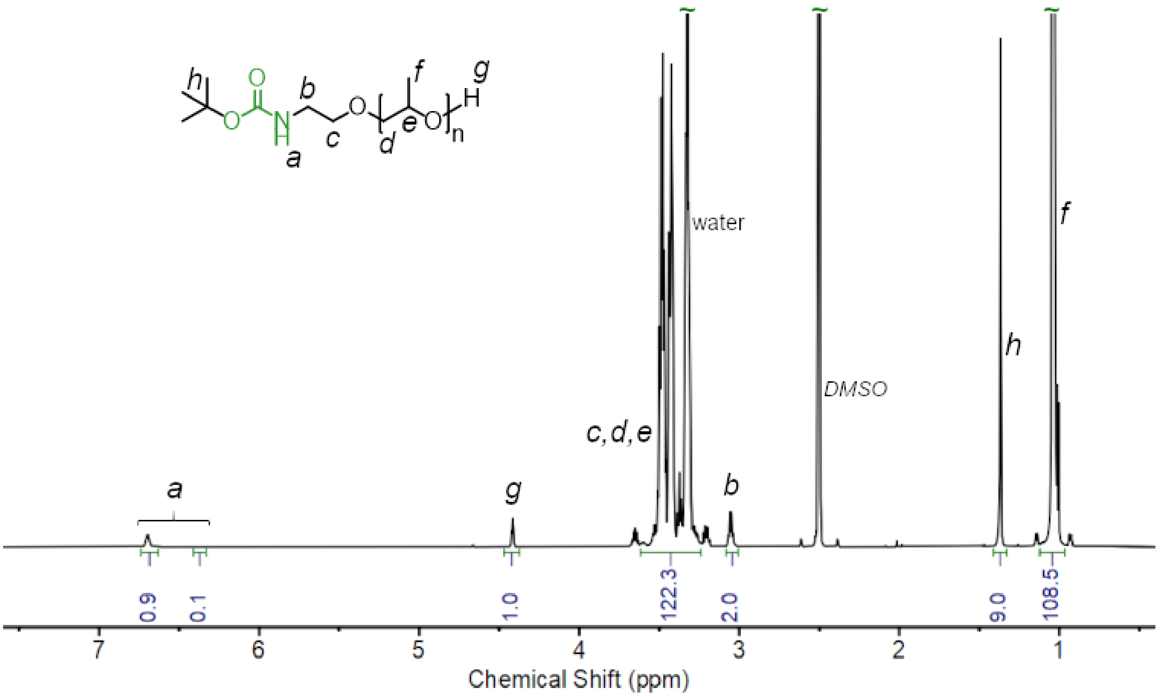

Figure S2. ^{1}H NMR spectrum of BocEA-PPO (Table 1, entry 2) prepared by using ^{t}BuP$_1$ as catalyst.

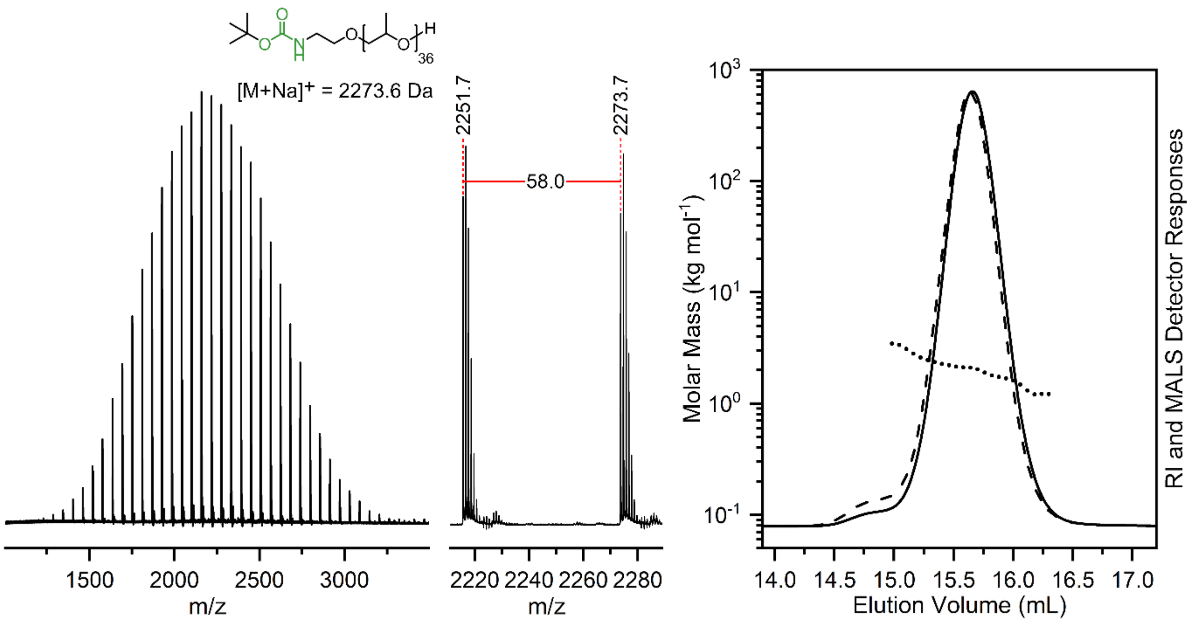

Figure S3. Left: MALDI-TOF mass spectrum and its enlarged region with denoted measured monoisotopic signals for BocEA-PPO (Table 1, entry 2) using ^{t}BuP$_1$ as catalyst. Right: SEC-MALS-RI chromatogram of BocEA-PPO. The solid and dashed lines represent the RI and 90° LS detector responses, respectively, while the dotted line represents molar mass as a function of elution volume.

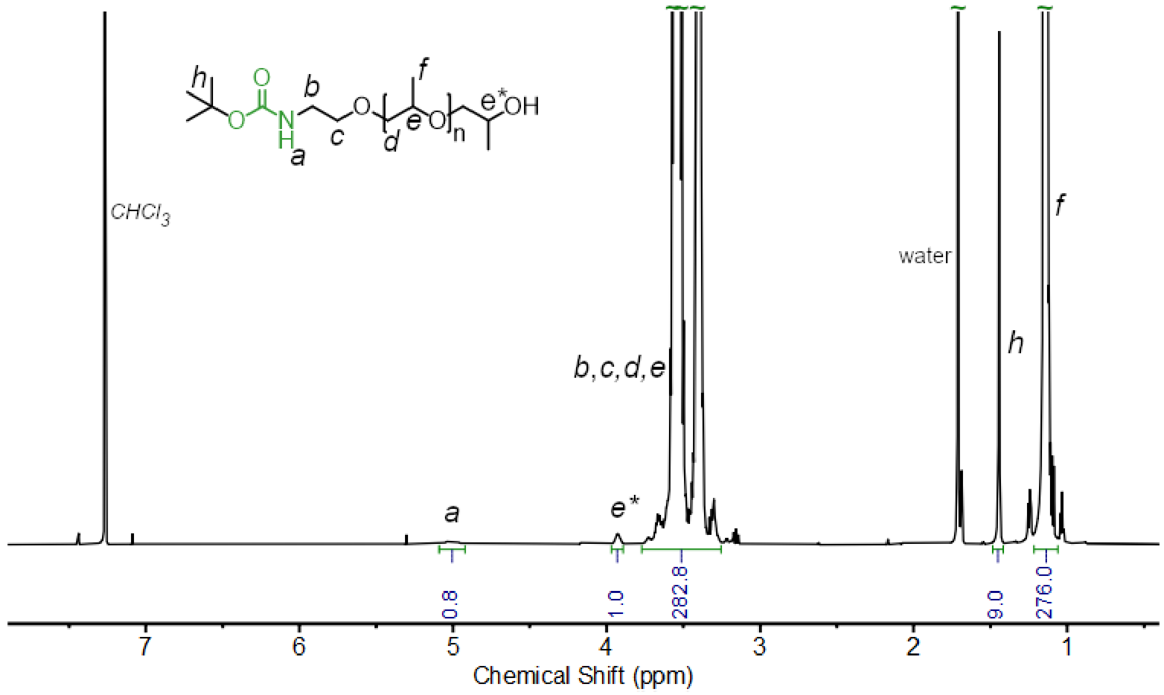

Figure S4. ^{1}H NMR spectrum of BocEA-PPO in $CDCl_3$ (Table 1, entry 3).

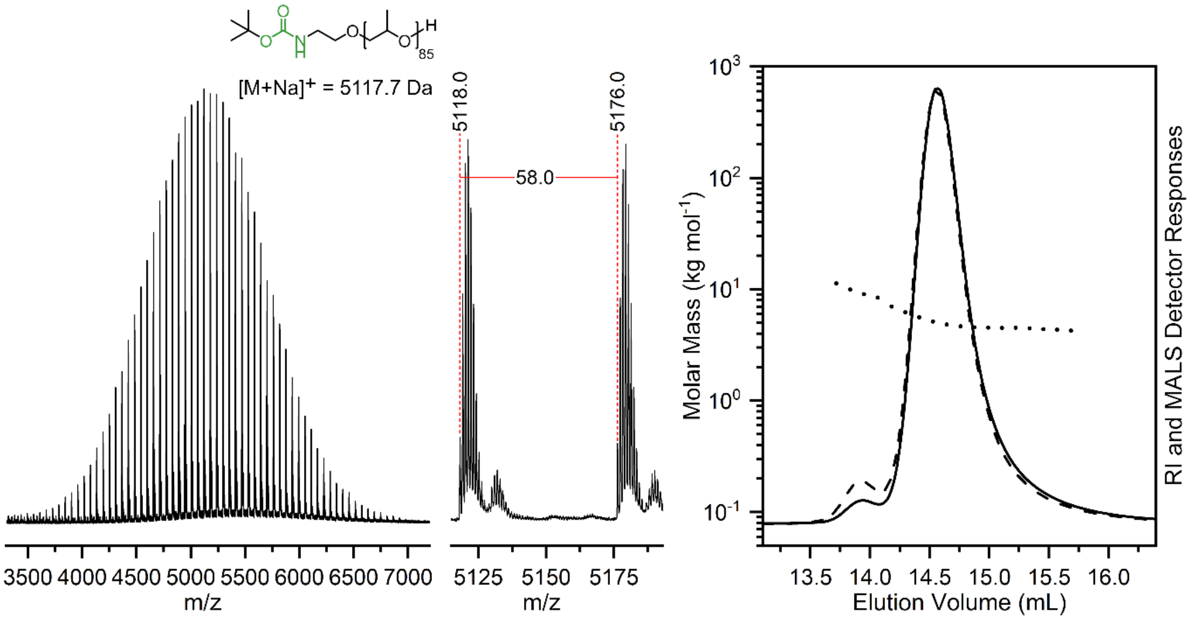

Figure S5. Left: MALDI-TOF mass spectrum and its enlarged region with denoted measured monoisotopic signals for BocEA-PPO (Table 1, entry 3). Additional low intensity peak distribution (Δ = –46.9 Da) corresponds to the species formed by Boc fragmentation during MALDI-TOF MS measurements. Right: SEC-MALS-RI chromatogram of BocEA-PPO. The solid and dashed lines represent the RI and 90° LS detector responses, respectively, while the dotted line represents molar mass as a function of elution volume.

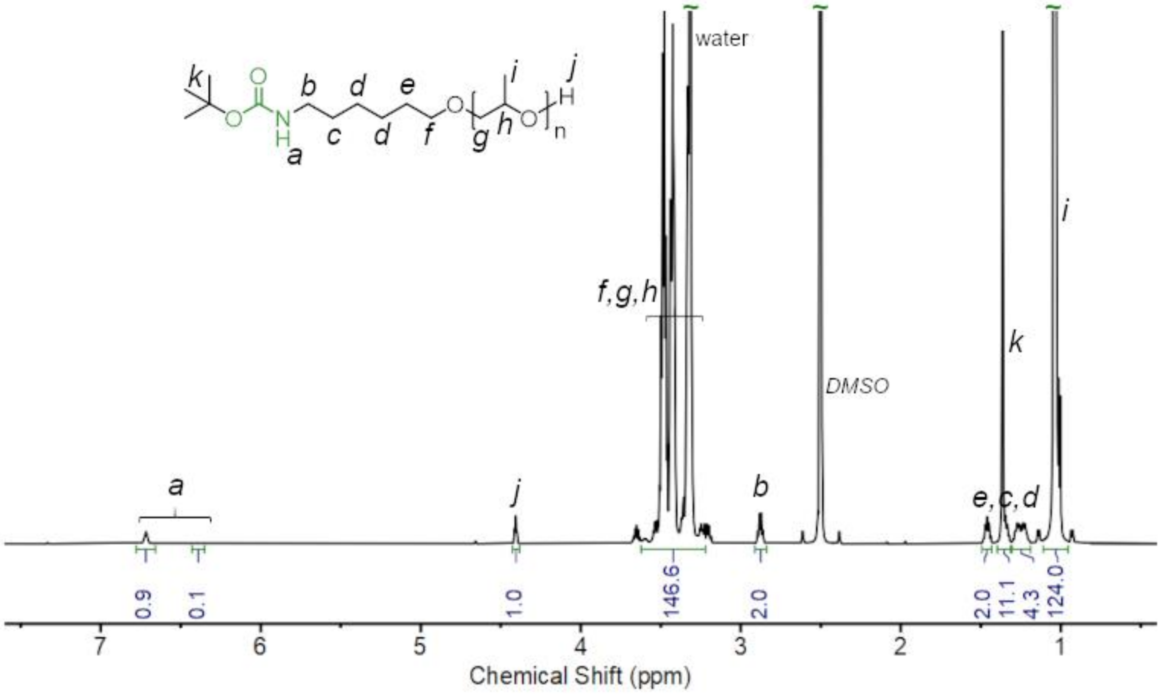

Figure S6. ^{1}H NMR spectrum of BocHA-PPO (Table 1, entry 4).

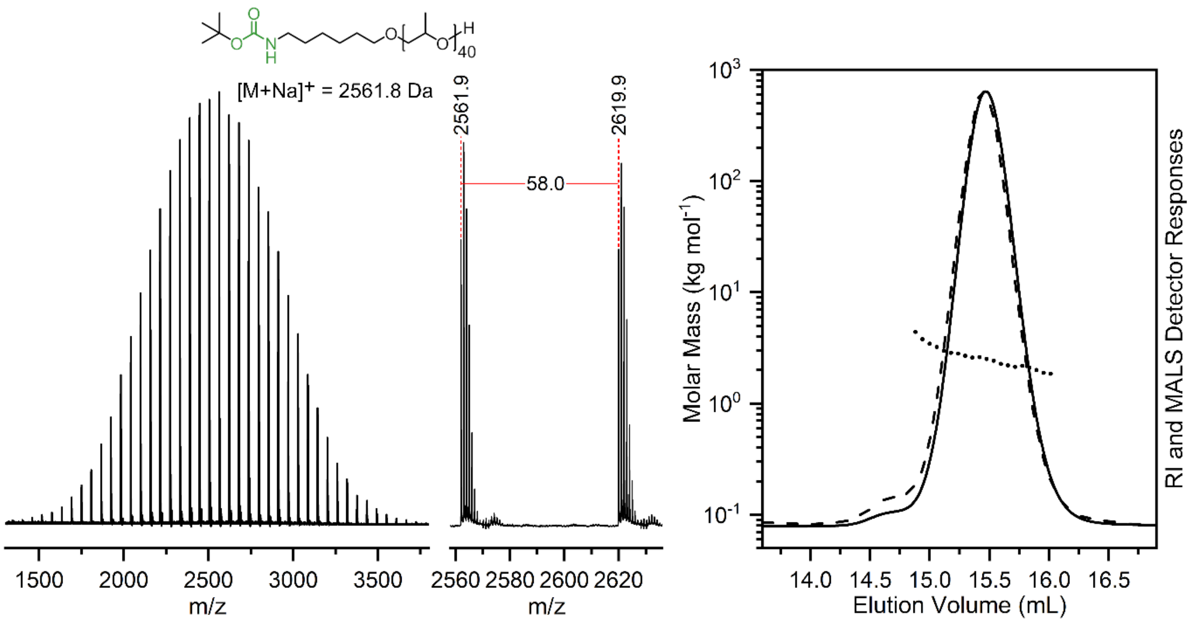

Figure S7. Left: MALDI-TOF mass spectrum and its enlarged region with denoted measured monoisotopic signals for BocHA-PPO (Table 1, entry 4). Additional low intensity peak distribution (Δ = –46.9 Da) corresponds to the species formed by Boc fragmentation during MALDI-TOF MS measurements. Right: SEC-MALS-RI chromatogram of BocHA-PPO. The solid and dashed lines represent the RI and 90° LS detector responses, respectively, while the dotted line represents molar mass as a function of elution volume.

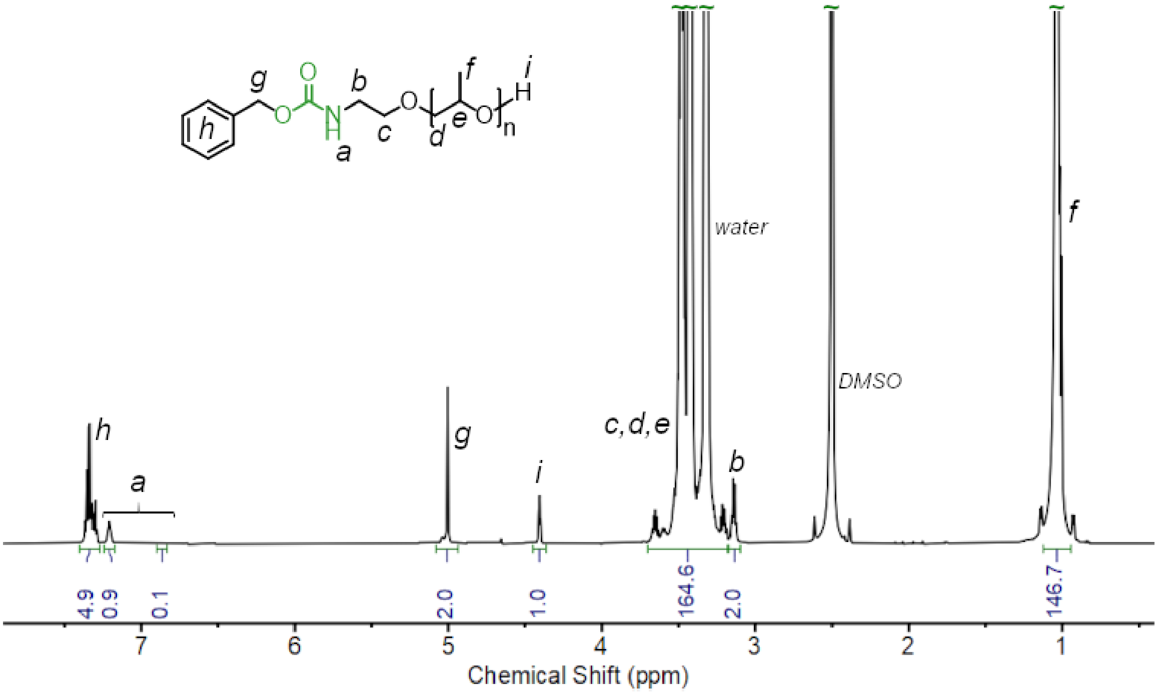

Figure S8. ^{1}H NMR spectrum of CbzEA-PPO (Table 1, entry 5).

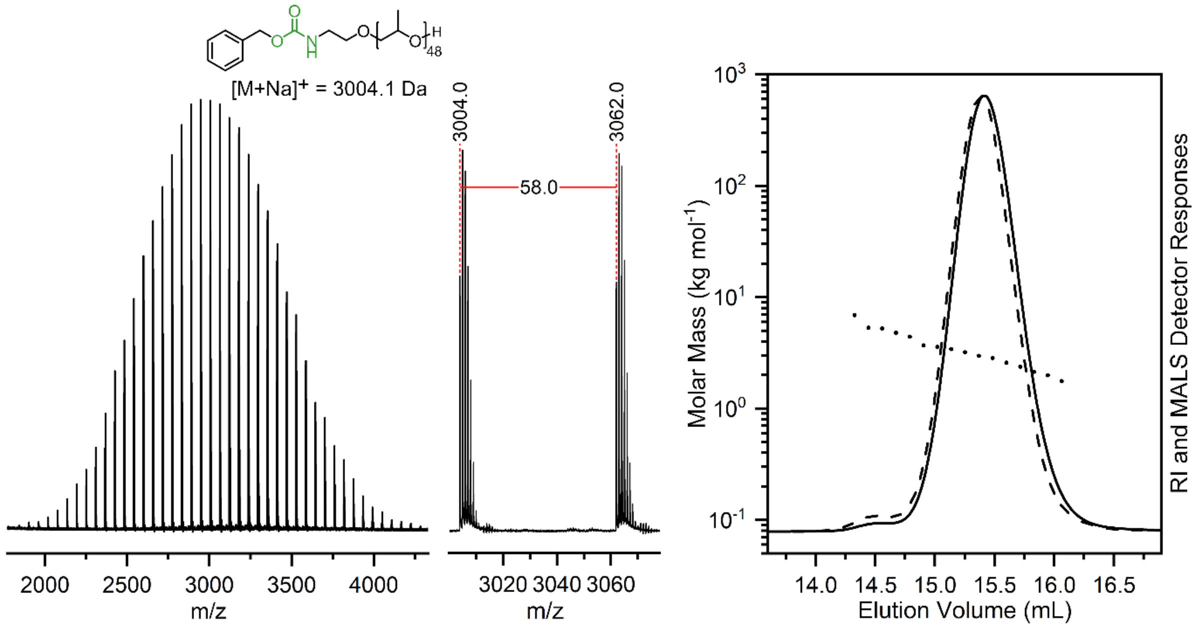

Figure S9. Left: MALDI-TOF mass spectrum and its enlarged region with denoted measured monoisotopic signals for CbzEA-PPO (Table 1, entry 5). Right: SEC-MALS-RI chromatogram of CbzEA-PPO. The solid and dashed lines represent the RI and 90° LS detector responses, respectively, while the dotted line represents molar mass as a function of elution volume.

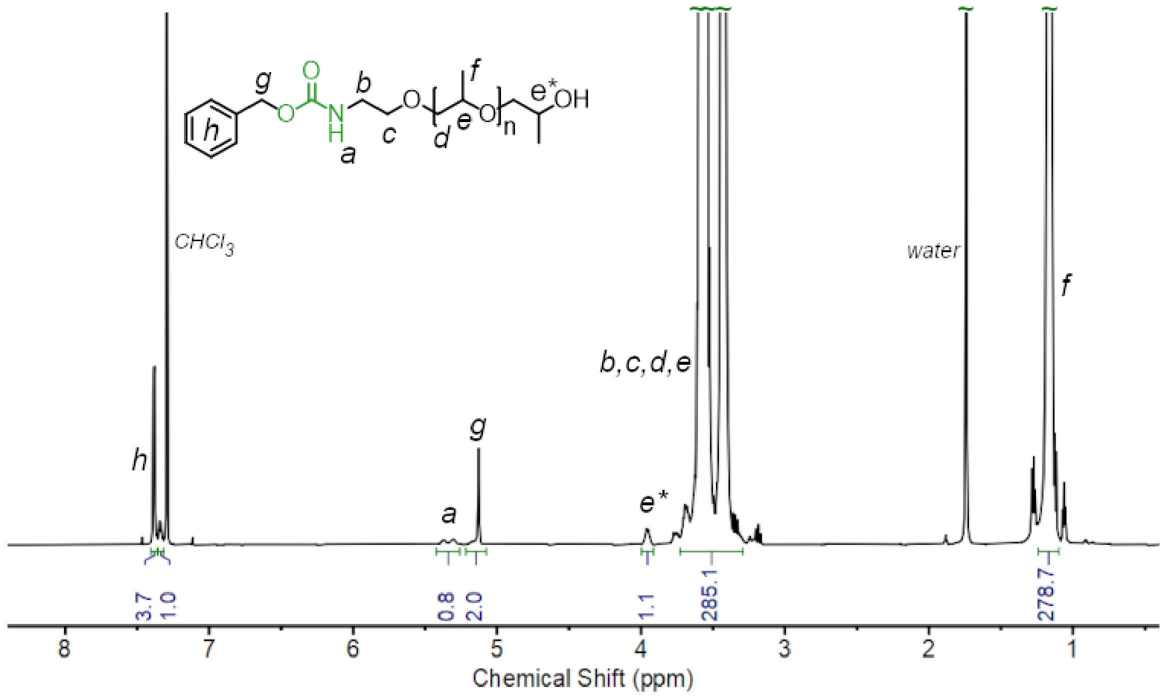

Figure S10. ^{1}H NMR spectrum of CbzEA-PPO in $CDCl_3$ (Table 1, entry 6).

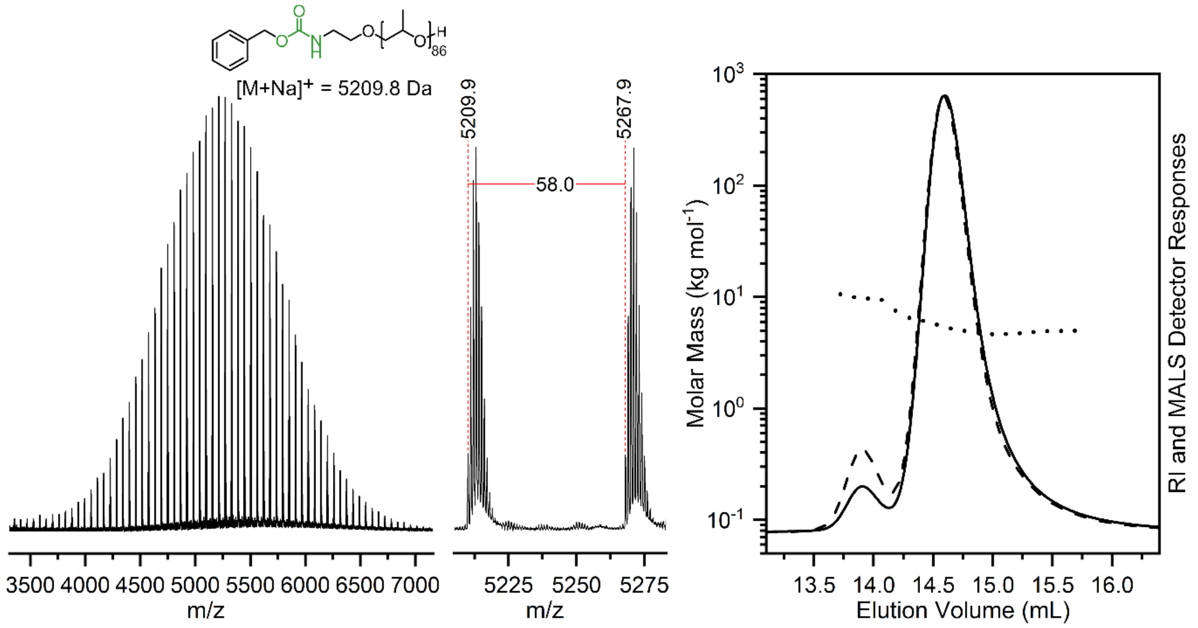

Figure S11. Left: MALDI-TOF mass spectrum and its enlarged region with denoted measured monoisotopic signals for CbzEA-PPO (Table 1, entry 6). Right: SEC-MALS-RI chromatogram of CbzEA-PPO. The solid and dashed lines represent the RI and 90° LS detector responses, respectively, while the dotted line represents molar mass as a function of elution volume.

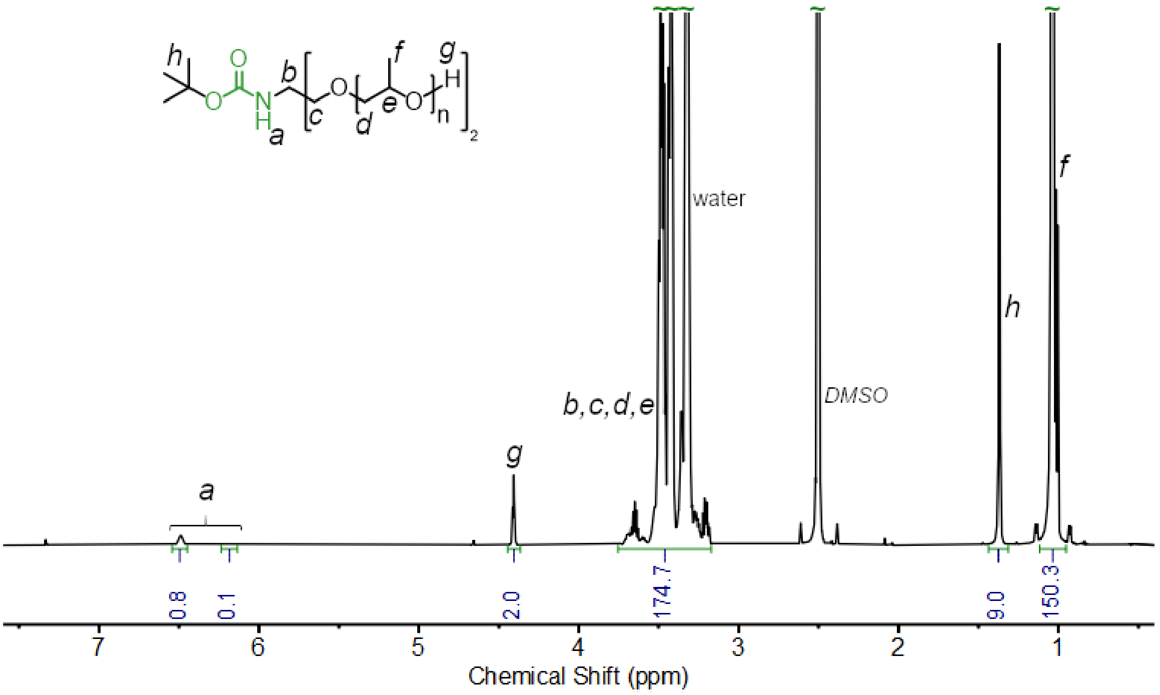

Figure S12. ^{1}H NMR spectrum of BocSr-(PPO)$_2$ (Table 1, entry 8).

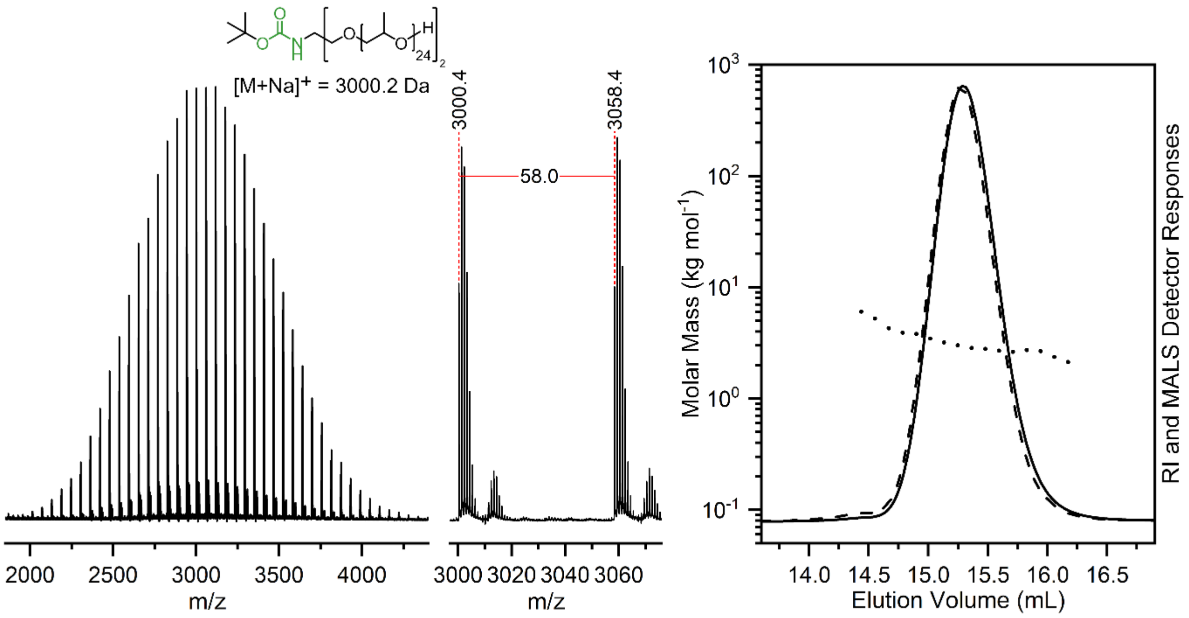

Figure S13. Left: MALDI-TOF mass spectrum and its enlarged region with denoted measured monoisotopic signals for BocSr-$(PPO)_2$ (Table 1, entry 8). Additional low intensity peak distribution (Δ = –46.9 Da) corresponds to the species formed by Boc fragmentation during MALDI-TOF MS measurements. Right: SEC-MALS-RI chromatogram of BocSr-$(PPO)_2$. The solid and dashed lines represent the RI and 90° LS detector responses, respectively, while the dotted line represents molar mass as a function of elution volume.

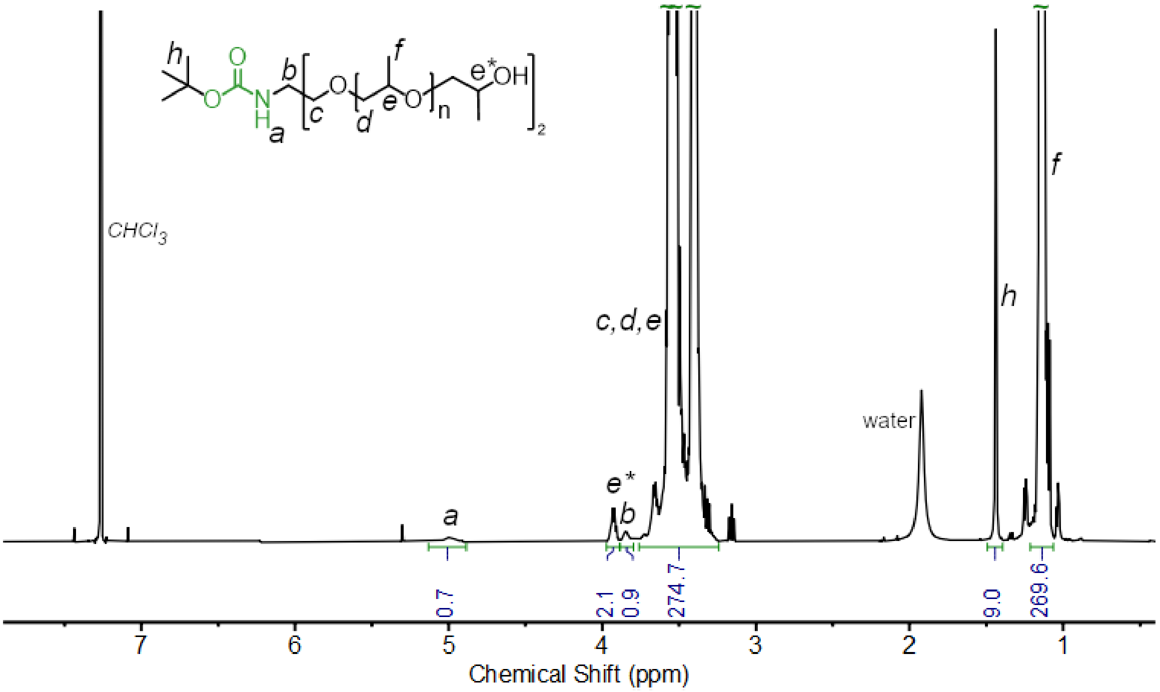

Figure S14. ^{1}H NMR spectrum of BocSr-$(PPO)_2$ in $CDCl_3$ (Table 1, entry 9).

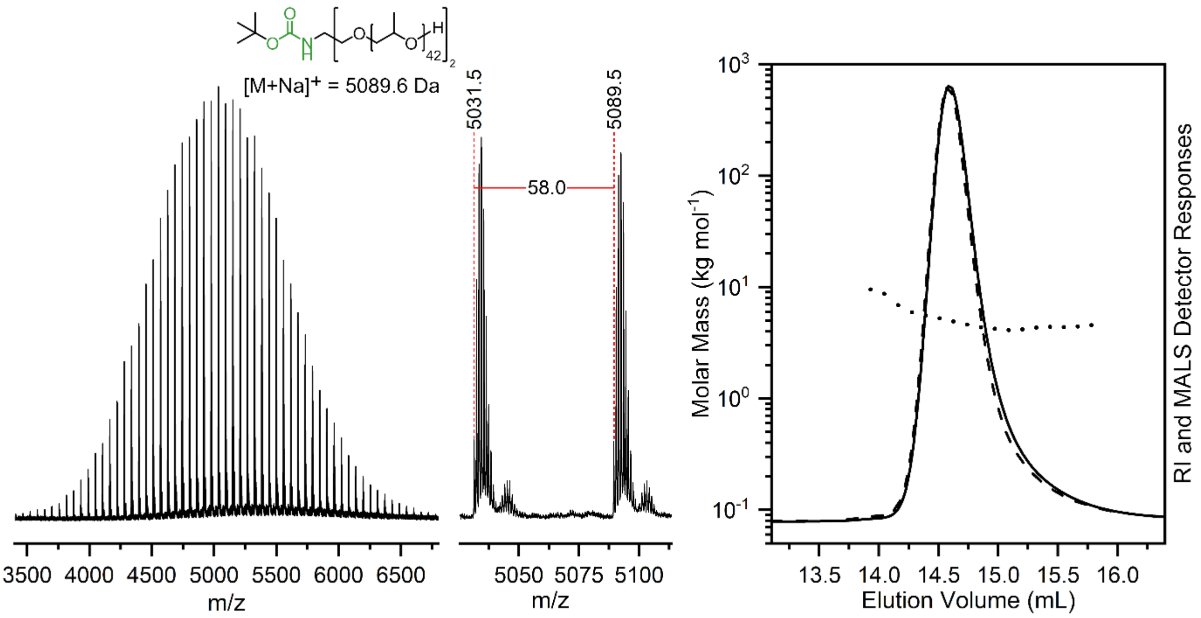

Figure S15. Left: MALDI-TOF mass spectrum and its enlarged region with denoted measured monoisotopic signals for BocSr-$(PPO)_2$ (Table 1, entry 9). Additional low intensity peak distribution (Δ = –46.9 Da) corresponds to the species formed by Boc fragmentation during MALDI-TOF MS measurements. Right: SEC-MALS-RI chromatogram of BocSr-$(PPO)_2$. The solid and dashed lines represent the RI and 90 °LS detector responses, respectively, while the dotted line represents molar mass as a function of elution volume.

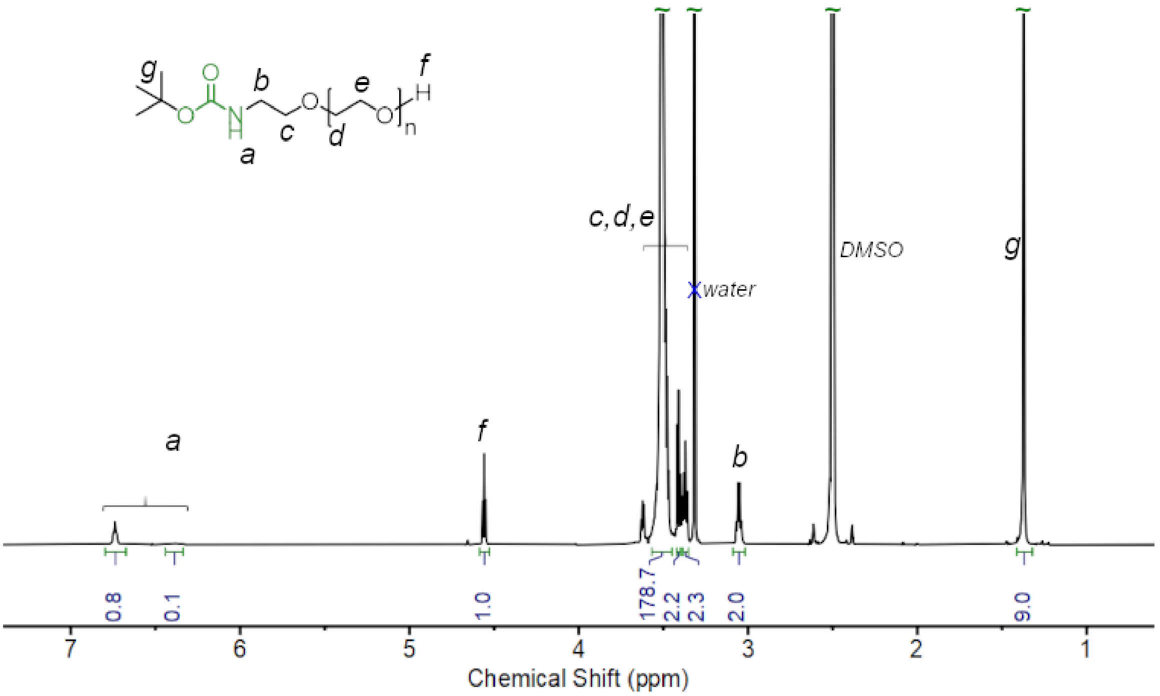

Figure S16. ^{1}H NMR spectrum of BocEA-PEO (Table 1, entry 10).

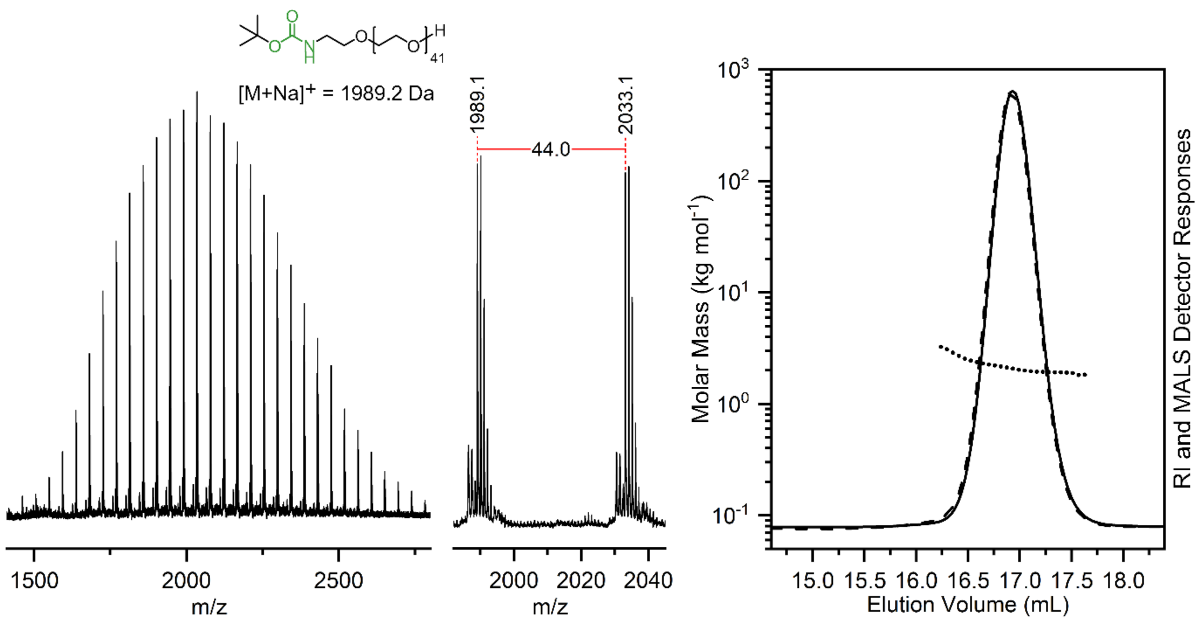

Figure S17. Left: MALDI-TOF mass spectrum and its enlarged region with denoted measured monoisotopic signals for BocEA-PEO (Table 1, entry 10). Additional, low intensity peak distribution (Δ = –46.9 Da) corresponds to the species formed by Boc fragmentation during MALDI-TOF MS measurements. Right: SEC-MALS-RI chromatogram of BocEA-PEO. The solid and dashed lines represent the RI and 90° LS responses, respectively, while the dotted line represents molar mass as a function of elution volume.

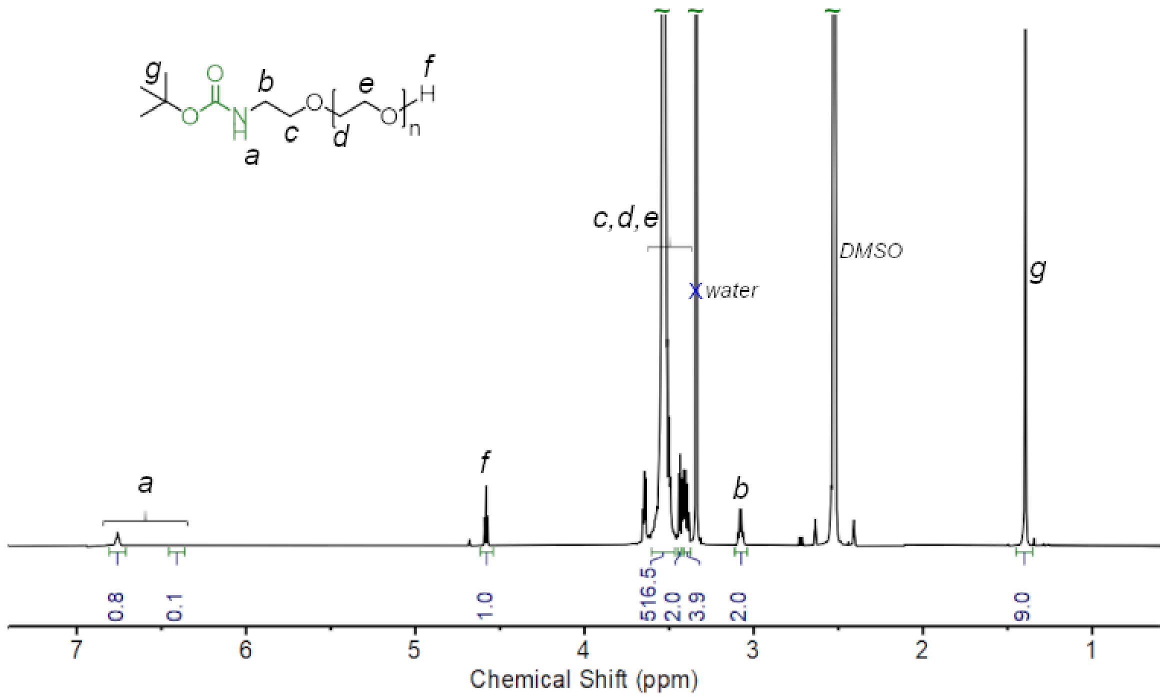

Figure S18. ^{1}H NMR spectrum of BocEA-PEO (Table 1, entry 11) prepared by using ^{t}BuP$_1$ as catalyst.

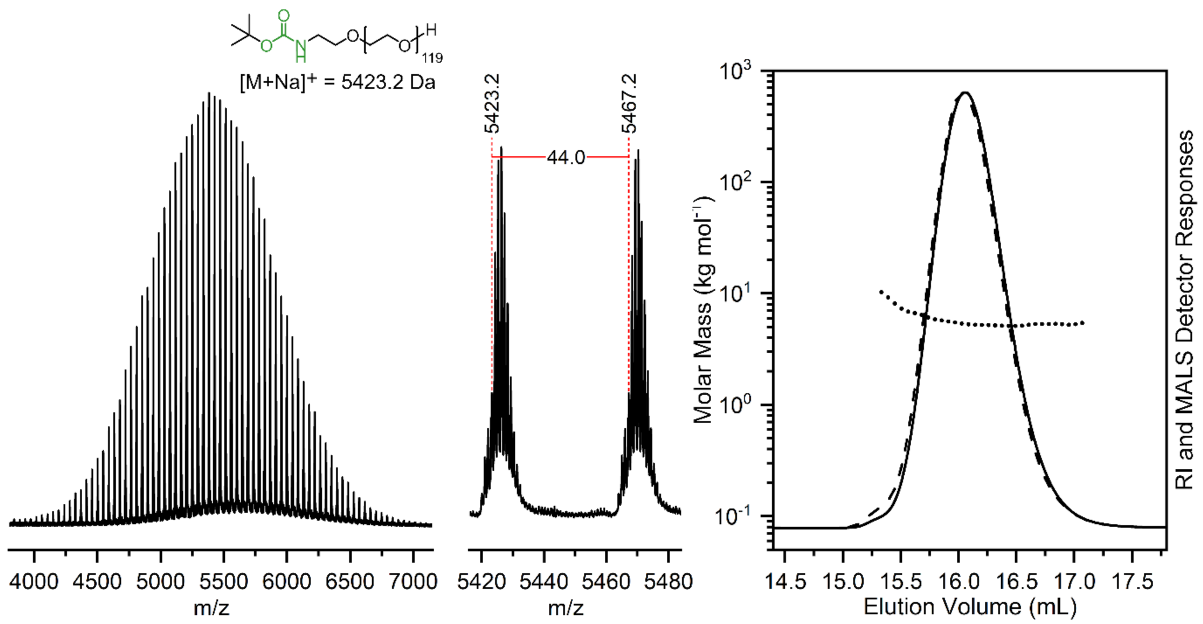

Figure S19. Left: MALDI-TOF mass spectrum and its enlarged region with denoted measured monoisotopic signals for BocEA-PEO (Table 1, entry 11) using ^{t}BuP$_1$ as catalyst. Right: SEC-MALS-RI chromatogram of BocEA-PEO. Additional, low intensity peak distribution (Δ = –46.9 Da) corresponds to the species formed by Boc fragmentation during MALDI-TOF MS measurements. The solid and dashed lines represent the RI and 90° LS detector responses, respectively, while the dotted line represents molar mass as a function of elution volume.

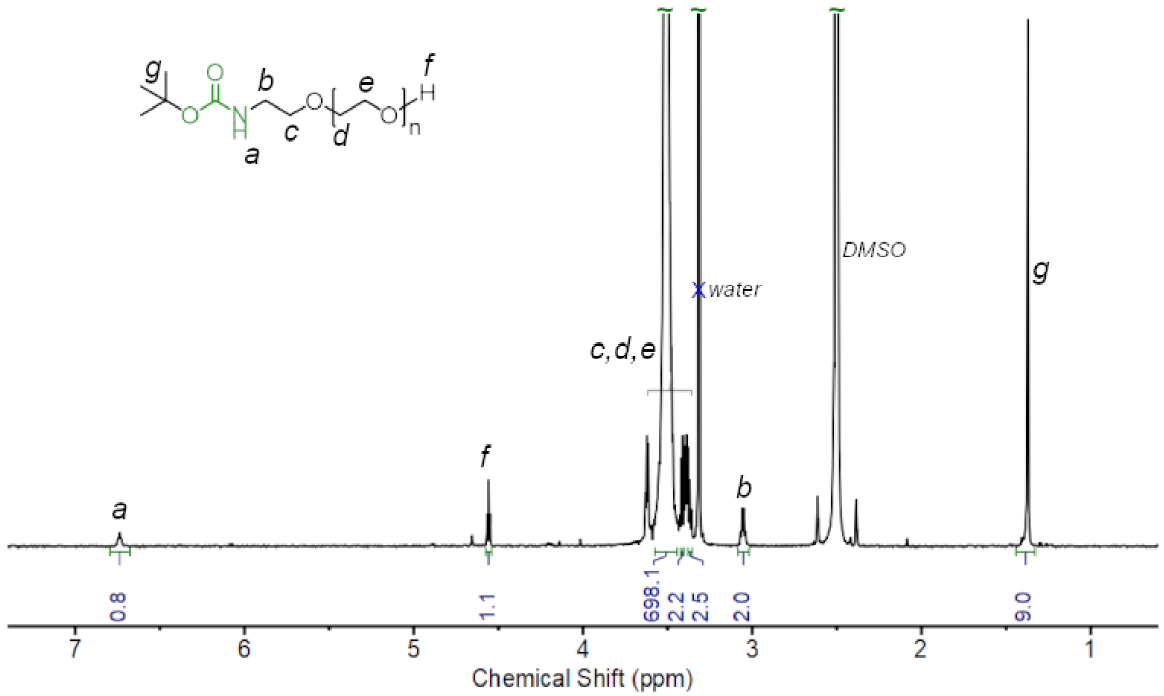

Figure S20. ^{1}H NMR spectrum of BocEA-PEO (Table 1, entry 12).

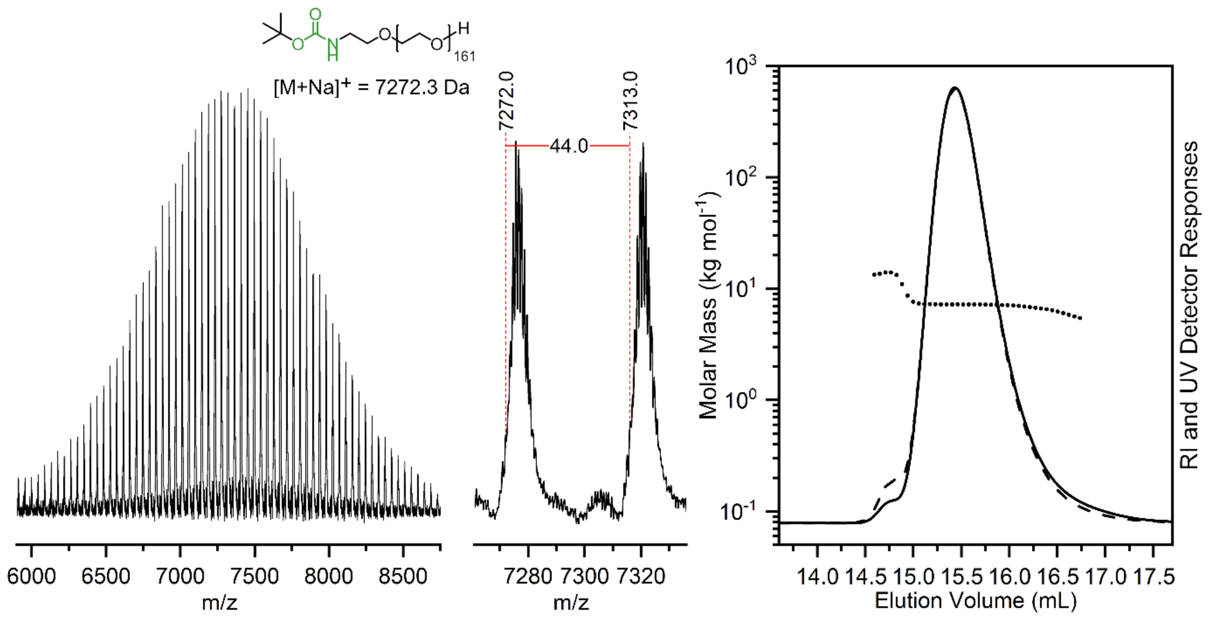

Figure S21. Left: MALDI-TOF mass spectrum and its enlarged region with denoted measured monoisotopic signals for BocEA-PEO (Table 1, entry 12). Right: SEC-MALS-RI chromatogram of BocEA-PEO. The solid and dashed lines represent the RI and 90° LS detector responses, respectively, while the dotted line represents molar mass as a function of elution volume.

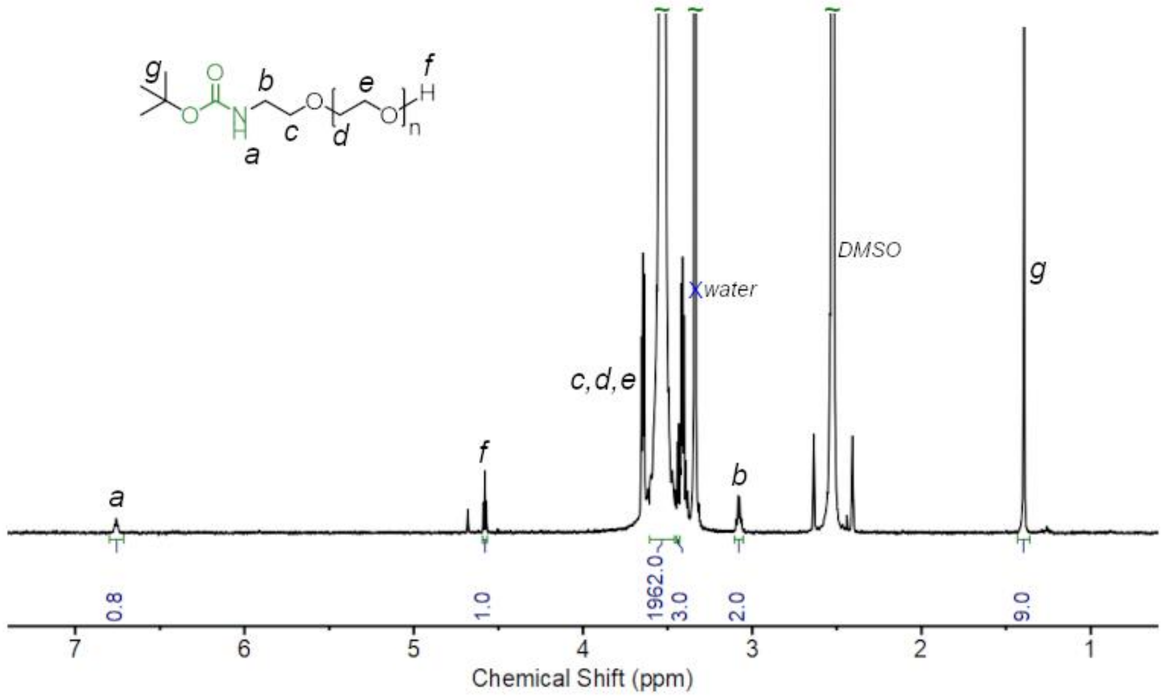

Figure S22. ^{1}H NMR spectrum of BocEA-PEO (Table 1, entry 13).

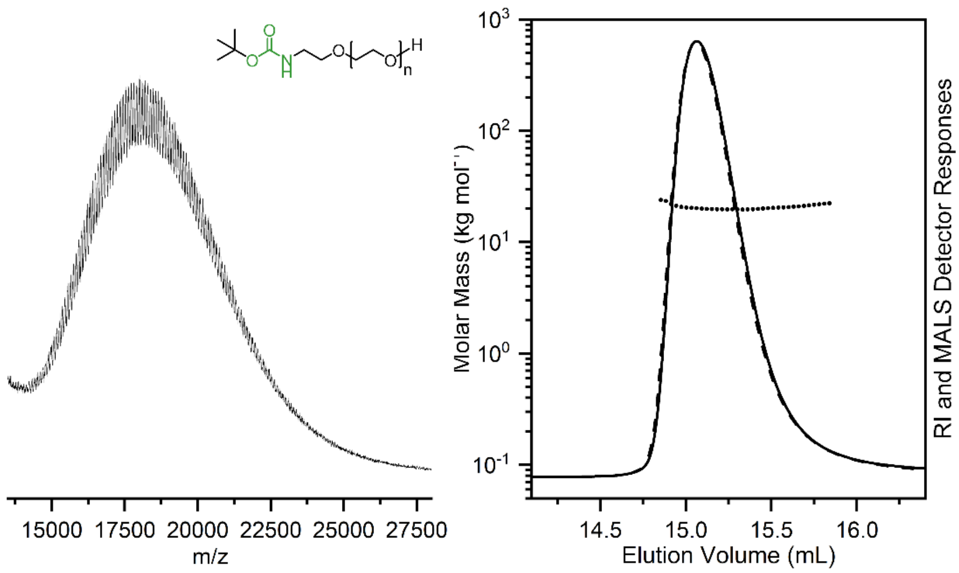

Figure S23. Left: MALDI-TOF mass spectrum for BocEA-PEO (Table 1, entry 13). Right: SEC-MALS-RI chromatogram of BocEA-PEO. The solid and dashed lines represent the RI and 90° LS detector responses, respectively, while the dotted line represents molar mass as a function of elution volume.

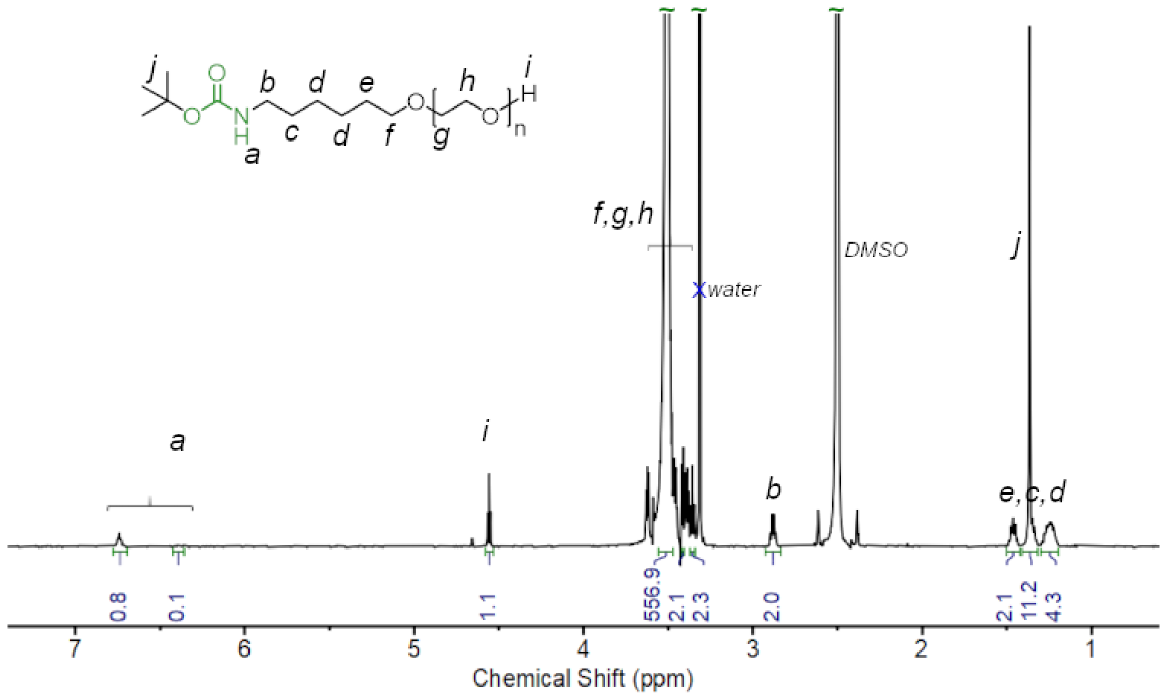

Figure S24. ^{1}H NMR spectrum of BocHA-PEO (Table 1, entry 14).

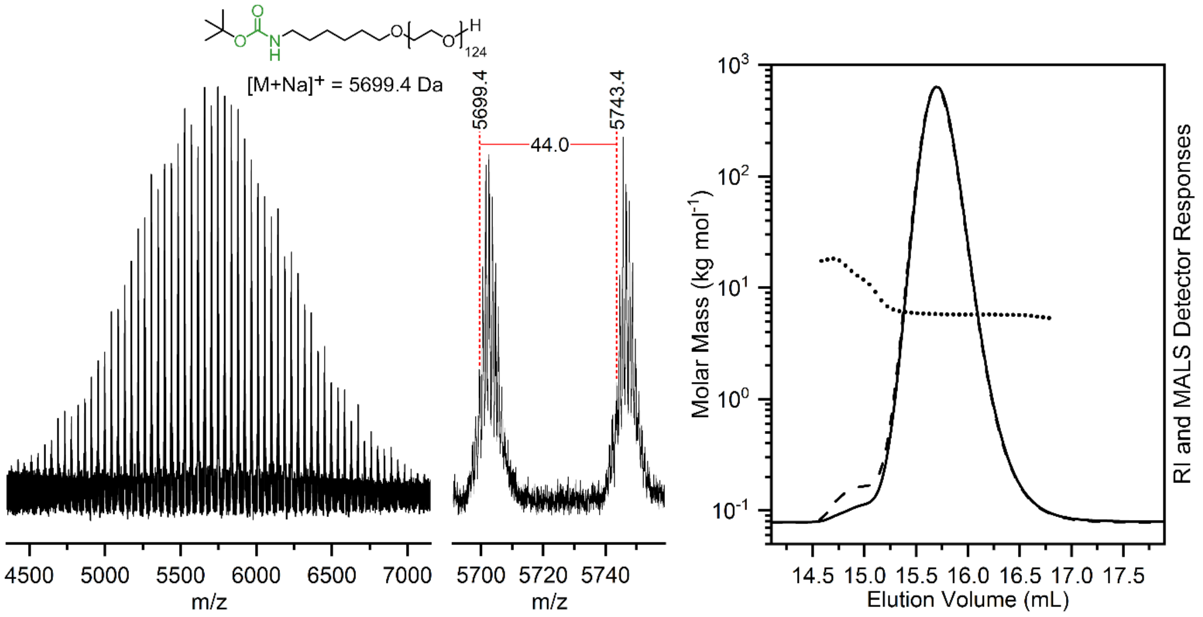

Figure S25. Left: MALDI-TOF mass spectrum and its enlarged region with denoted measured monoisotopic signals for BocHA-PEO (Table 1, entry 14). Additional low intensity peak distribution (Δ = –46.9 Da) corresponds to the species formed by Boc fragmentation during MALDI-TOF MS measurements. Right: SEC-MALS-RI chromatogram of BocHA-PEO. The solid and dashed lines represent the RI and 90° LS detector responses, respectively, while the dotted line represents molar mass as a function of elution volume.

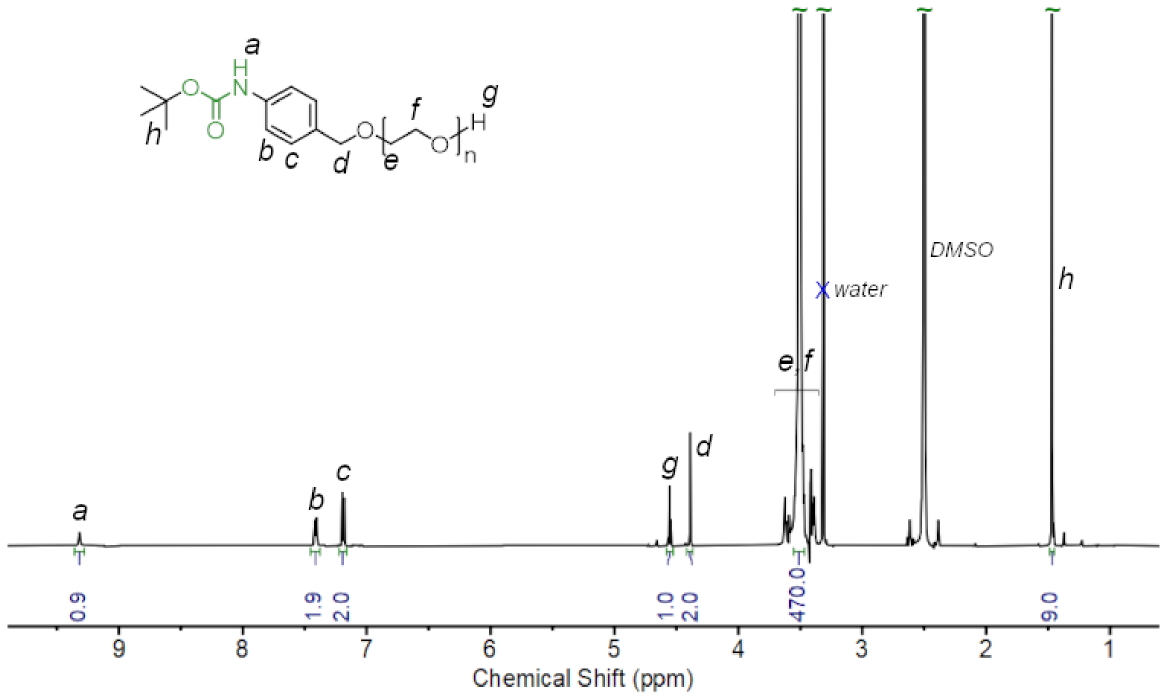

Figure S26. ^{1}H NMR spectrum of BocBA-PEO (Table 1, entry 15).

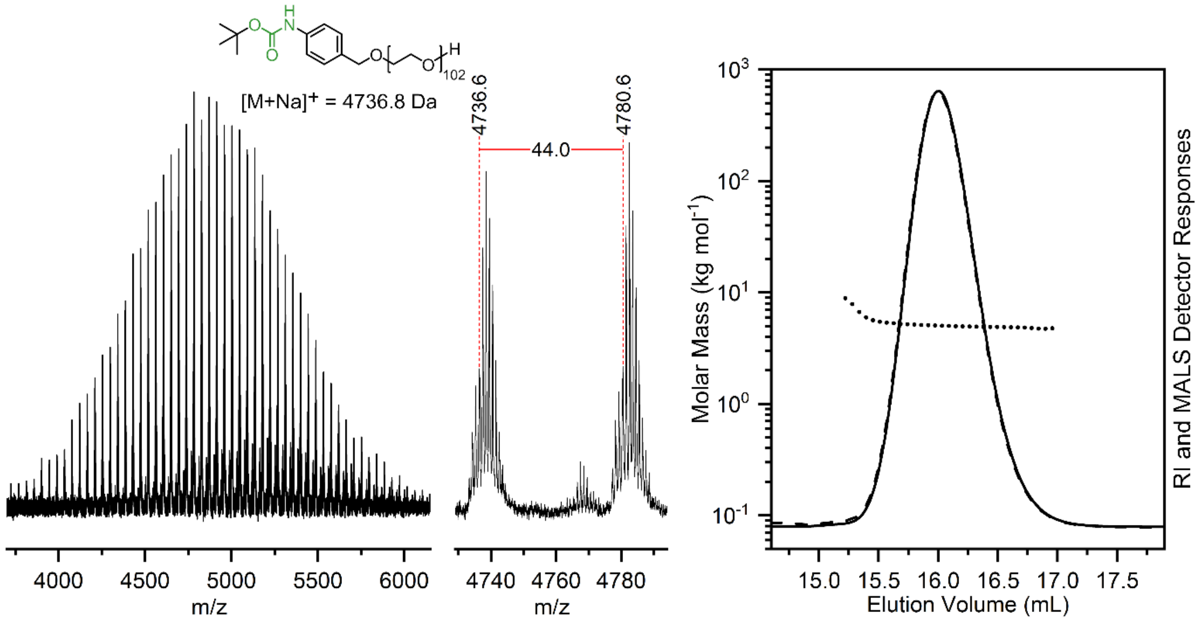

Figure S27. Left: MALDI-TOF mass spectrum and its enlarged region with denoted measured monoisotopic signals for BocBA-PEO (Table 1, entry 15). Additional low intensity peak distribution (Δ = –46.9 Da) corresponds to the species formed by Boc fragmentation during MALDI-TOF MS measurements. Right: SEC-MALS-RI chromatogram of BocBA-PEO. The solid and dashed lines represent the RI and 90° LS detector responses, respectively, while the dotted line represents molar mass as a function of elution volume.

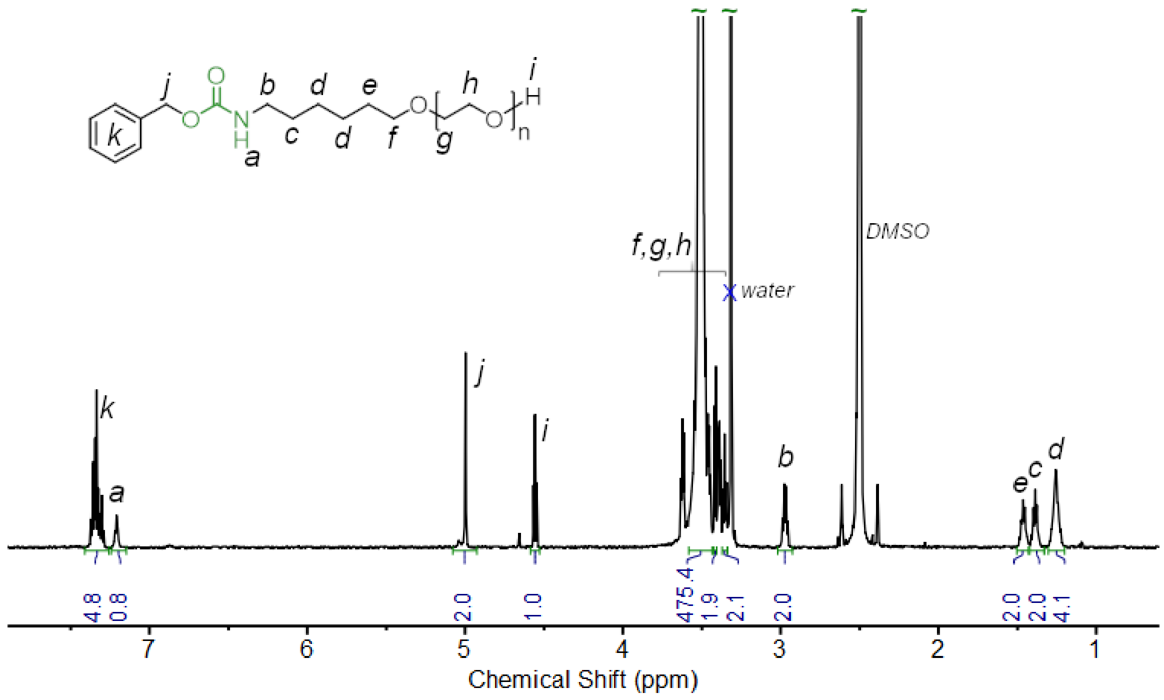

Figure S28. ^{1}H NMR spectrum of CbzHA-PEO (Table 1, entry 16).

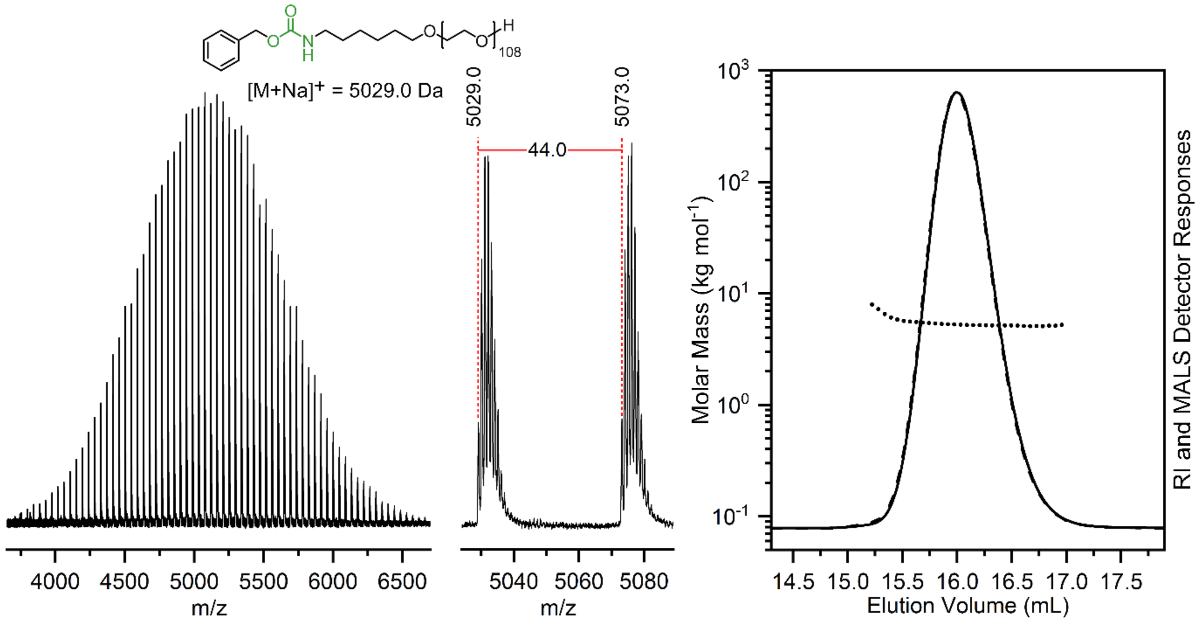

Figure S29. Left: MALDI-TOF mass spectrum and its enlarged region with denoted measured monoisotopic signals for CbzHA-PEO (Table 1, entry 16). Right: SEC-MALS-RI chromatogram of CbzHA-PEO. The solid and dashed lines represent the RI and 90° LS detector responses, respectively, while the dotted line represents molar mass as a function of elution volume.

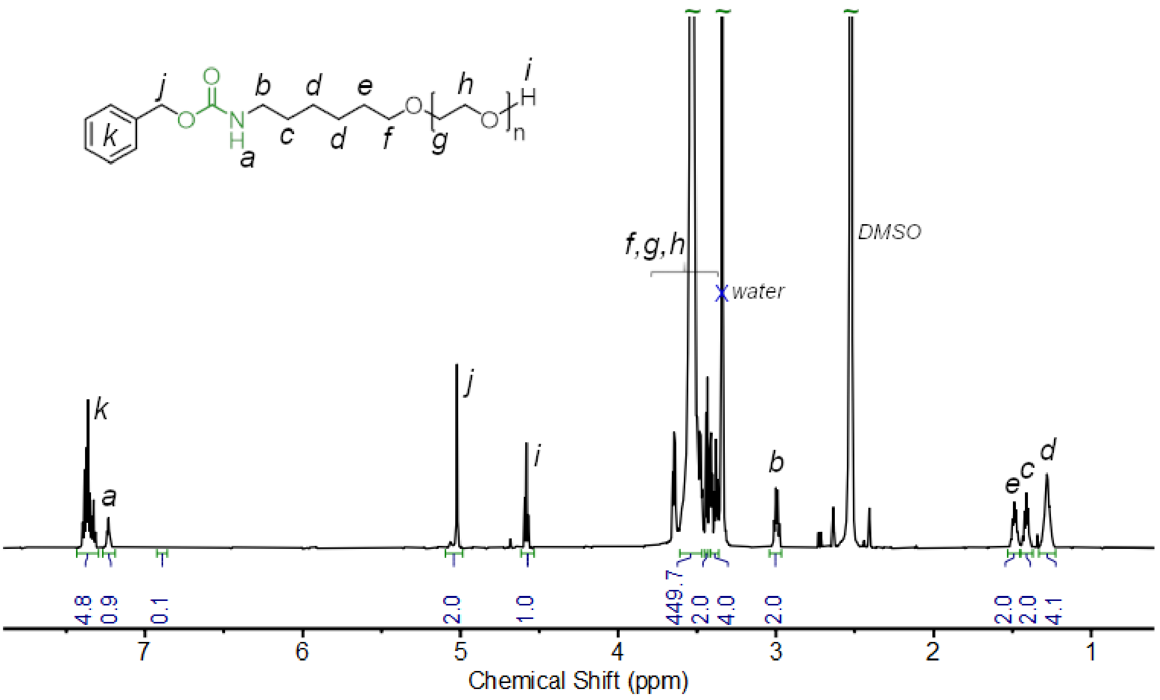

Figure S30. ^{1}H NMR spectrum of CbzHA-PEO (Table 1, entry 17) using ^{t}BuP$_1$ as a catalyst.

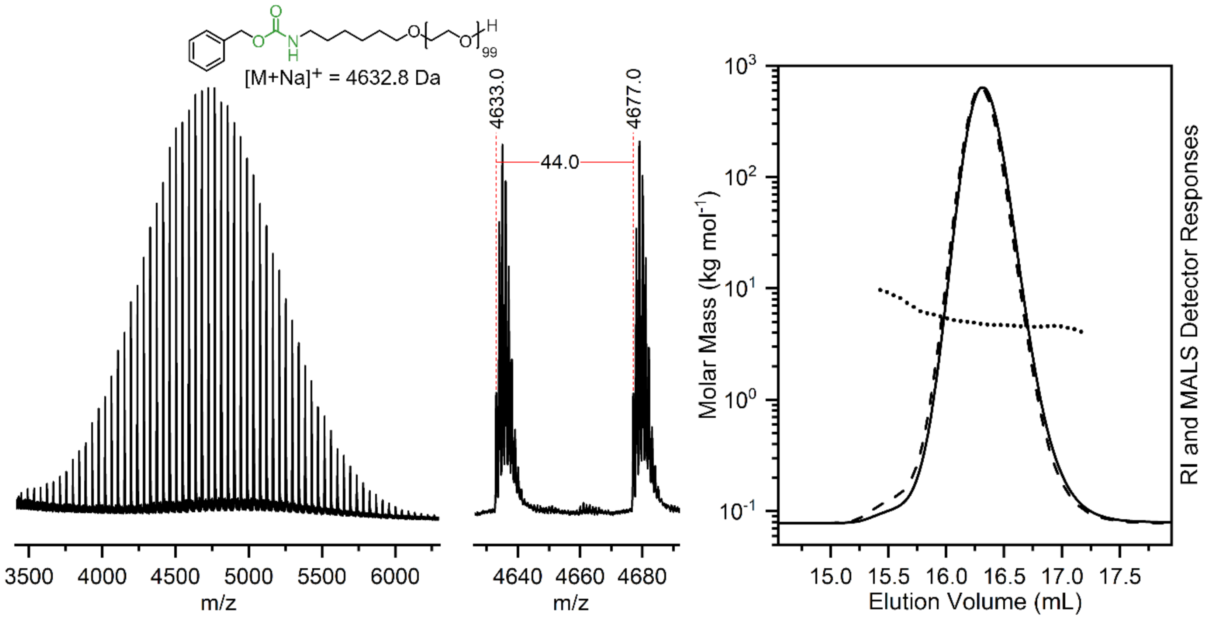

Figure S31. Left: MALDI-TOF mass spectrum and its enlarged region with denoted measured monoisotopic signals for CbzHA-PEO (Table 1, entry 17) using $^{t}BuP_1$ as a catalyst. Right: SEC-MALS-RI chromatogram of CbzHA-PEO. The solid and dashed lines represent the RI and 90° LS detector responses, respectively, while the dotted line represents molar mass as a function of elution volume.

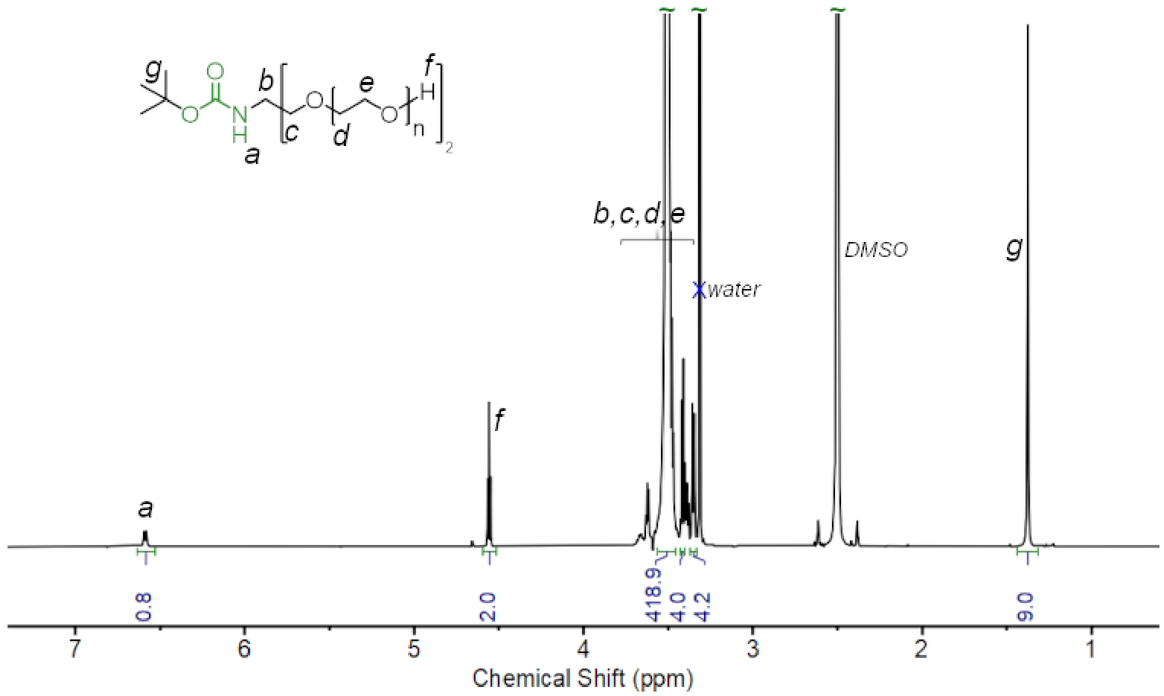

Figure S32. ^{1}H NMR spectrum of BocSr-(PEO)$_2$ (Table 1, entry 18).

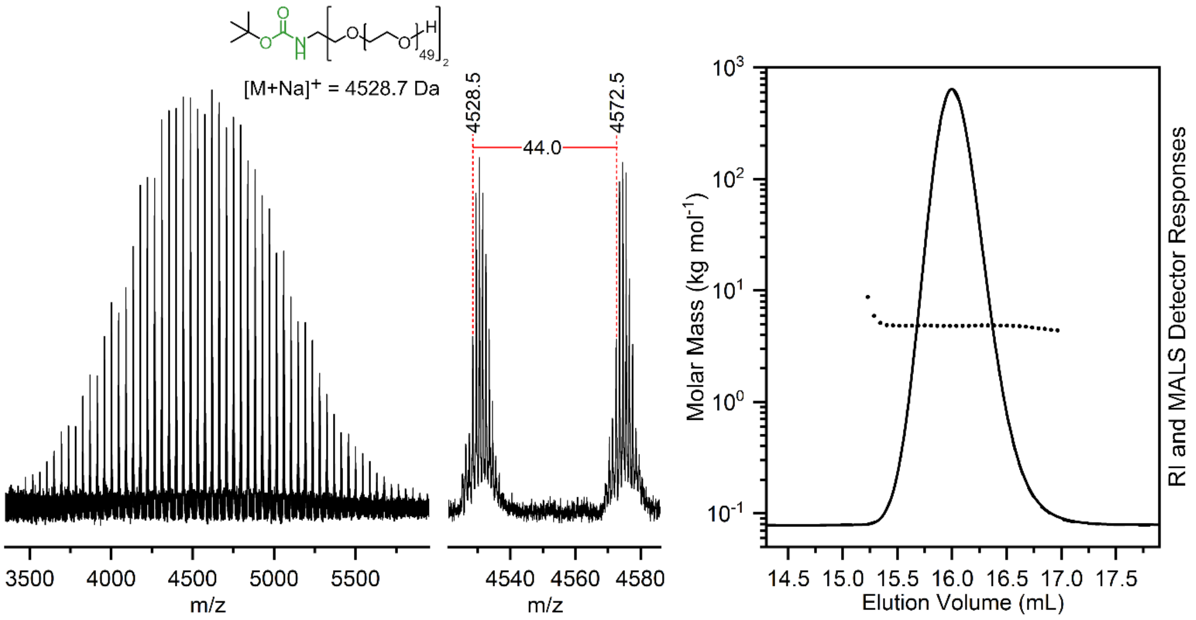

Figure S33. Left: MALDI-TOF mass spectrum and its enlarged region with denoted measured monoisotopic signals for BocSr-PEO$_2$ (Table 1, entry 18). Additional, low intensity peak distribution (Δ = –46.9 Da) corresponds to the species formed by Boc fragmentation during MALDI-TOF MS measurements. Right: SEC-MALS-RI chromatogram of BocSr-(PEO)$_2$. The solid and dashed lines represent the RI and 90° LS detector responses, respectively, while the dotted line represents molar mass as a function of elution volume.

S3. End-group modification and characterization

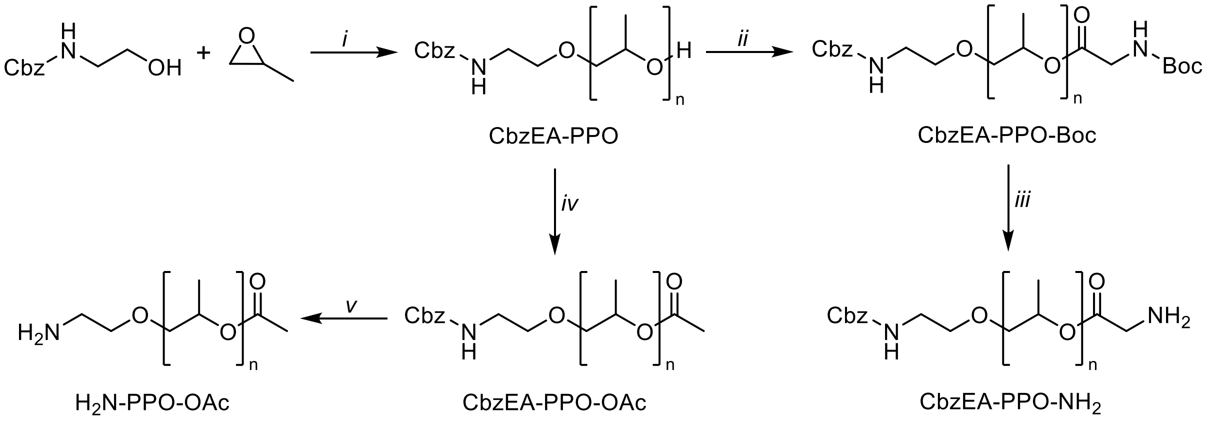

Scheme S1. *i*. ^{t}BuP$_2$, Et$_3$B, THF; *ii*. BocGly, EDC·HCl, DMAP, DCM; *iii*. TFA, chloroform or HCl, dioxane; *iv*. Ac$_2$O, Pyr, chloroform; *v*. H$_2$ (g), Pd/C, AcOH, DCM.

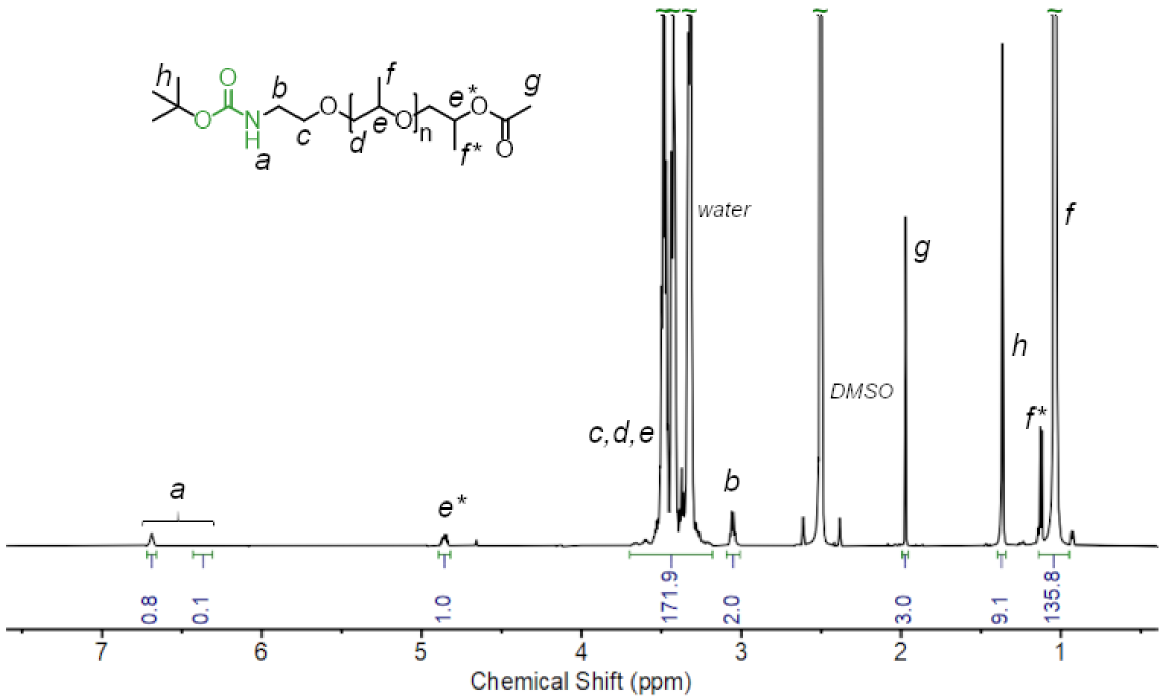

Figure S34. ^{1}H NMR spectrum of acetylated BocEA-PPO-OAc prepared from BocEA-PPO (Table 1, entry 1).

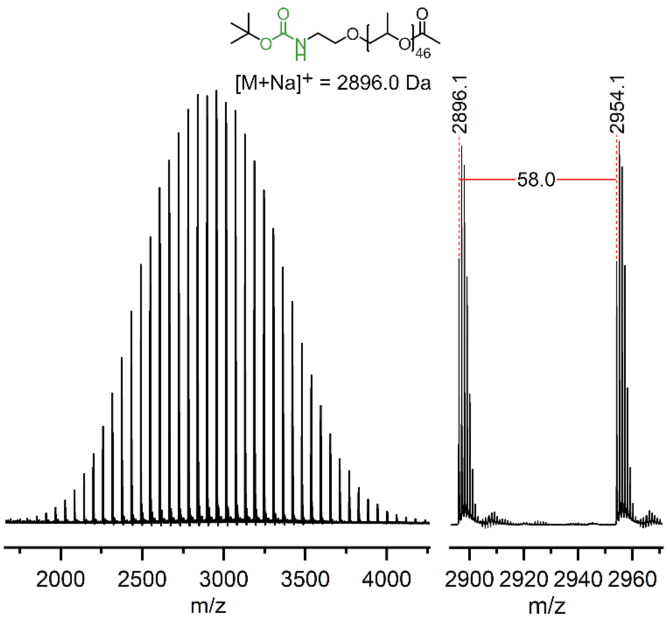

Figure S35. MALDI-TOF mass spectrum and its enlarged region with denoted measured monoisotopic signals for BocEA-PPO-OAc prepared from BocEA-PPO (Table 1, entry 1).

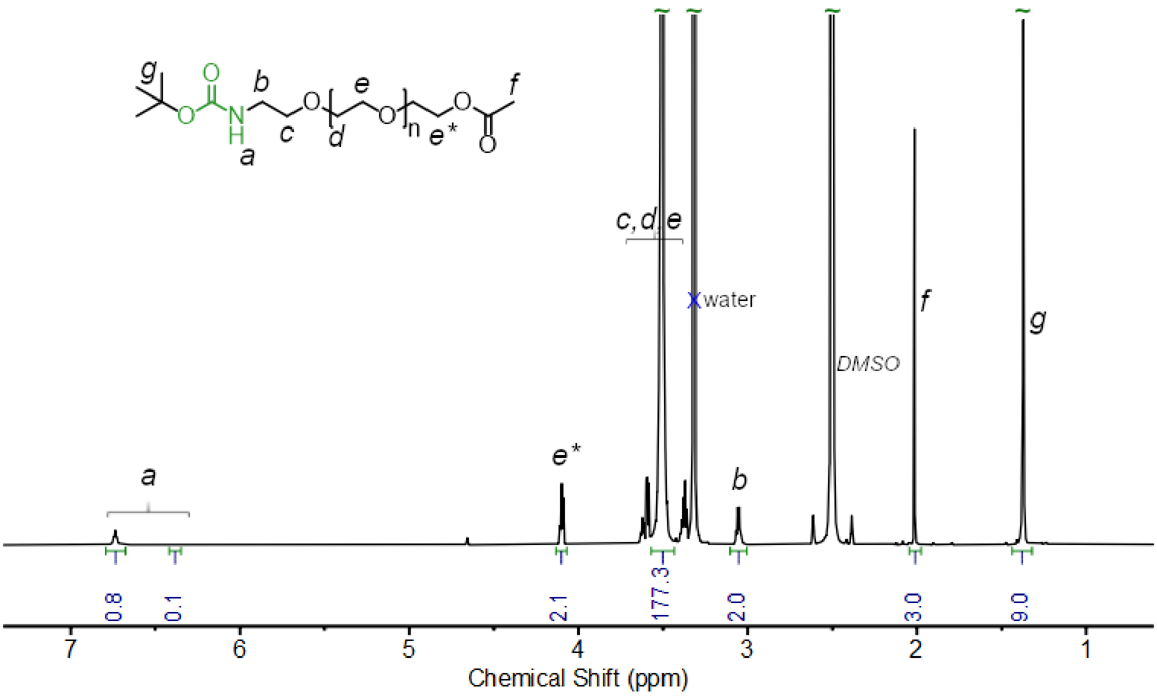

Figure S36. ^{1}H NMR spectrum of acetylated BocEA-PEO-OAc prepared from BocEA-PEO (Table 1, entry 10).

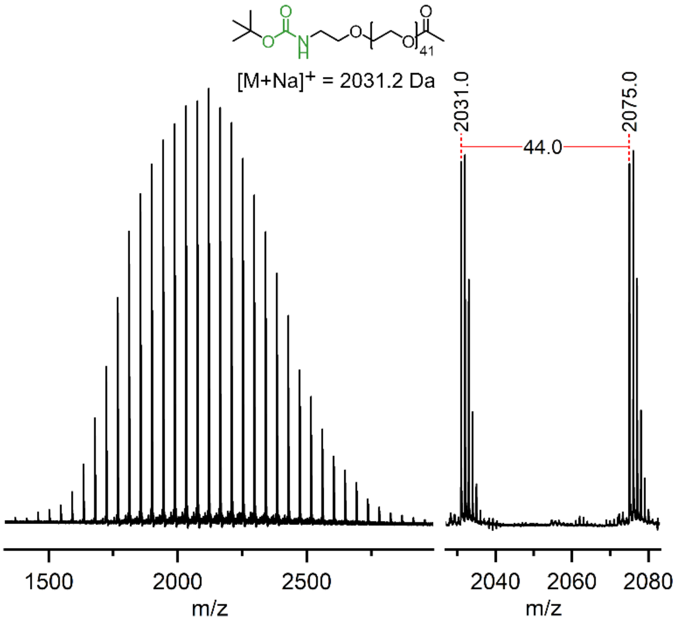

Figure S37. MALDI-TOF mass spectrum and its enlarged region with denoted measured monoisotopic signals for BocEA-PEO-OAc prepared from BocEA-PEO (Table 1, entry 10). Additional low intensity peak distribution (Δ = –46.9 Da) corresponds to the species formed by Boc fragmentation during MALDI-TOF MS measurements.

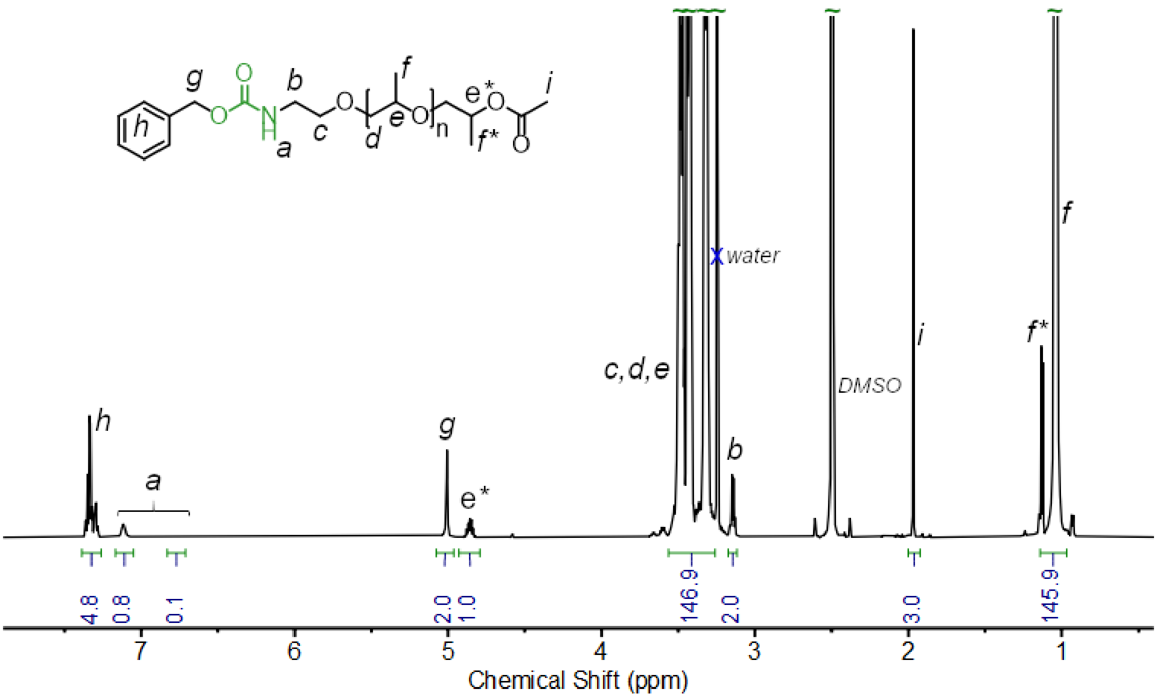

Figure S38. ^{1}H NMR spectrum of acetylated CbzEA-PPO-OAc prepared from CbzEA-PPO (Table 1, entry 5).

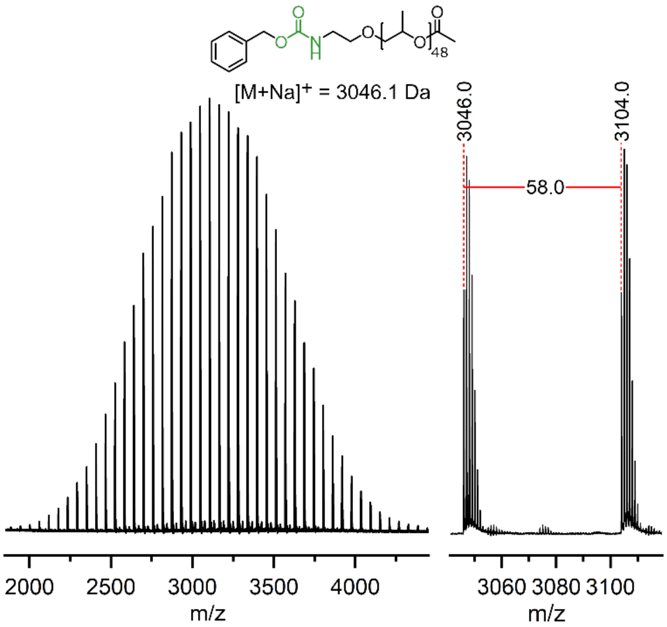

Figure S39. MALDI-TOF mass spectrum and its enlarged region with denoted measured monoisotopic signals for CbzEA-PPO-OAc prepared from CbzEA-PPO (Table 1, entry 5).

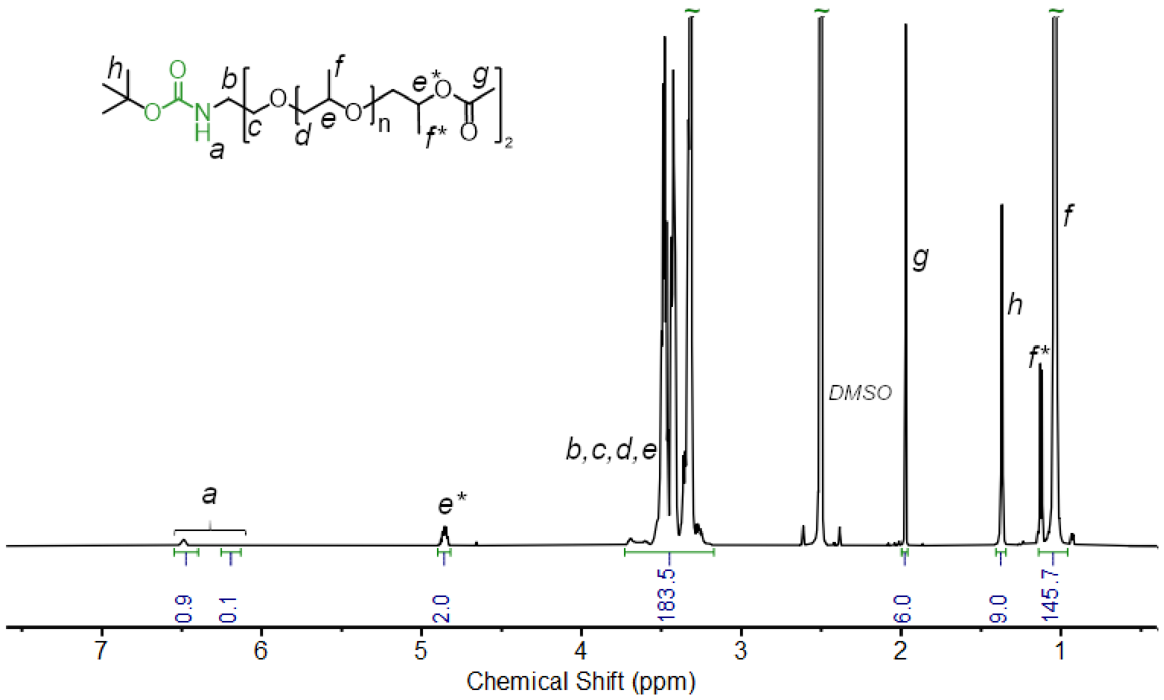

Figure S40. ^{1}H NMR spectrum of acetylated BocSr-(PPO-OAc)$_2$ prepared from BocSr-(PPO)$_2$ (Table 1, entry 8).

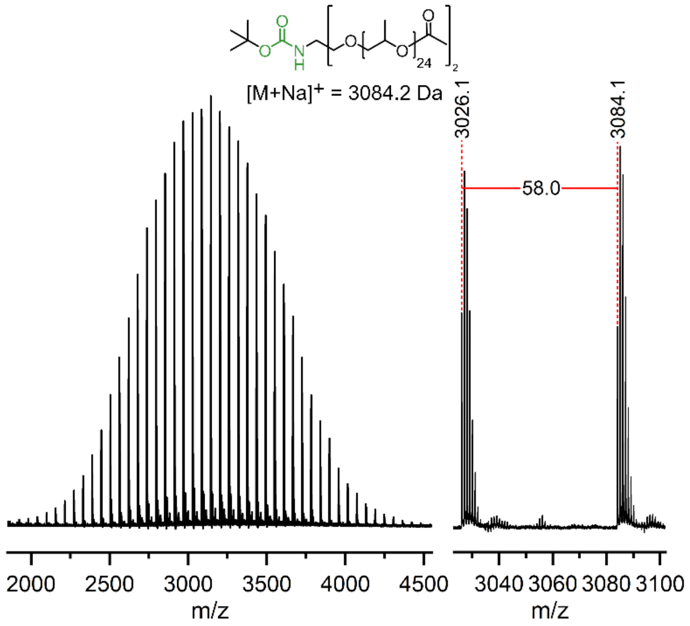

Figure S41. MALDI-TOF mass spectrum and its enlarged region with denoted measured monoisotopic signals for BocSr-(PPO-OAc)$_2$ prepared from BocSr-(PPO)$_2$ (Table 1, entry 8).

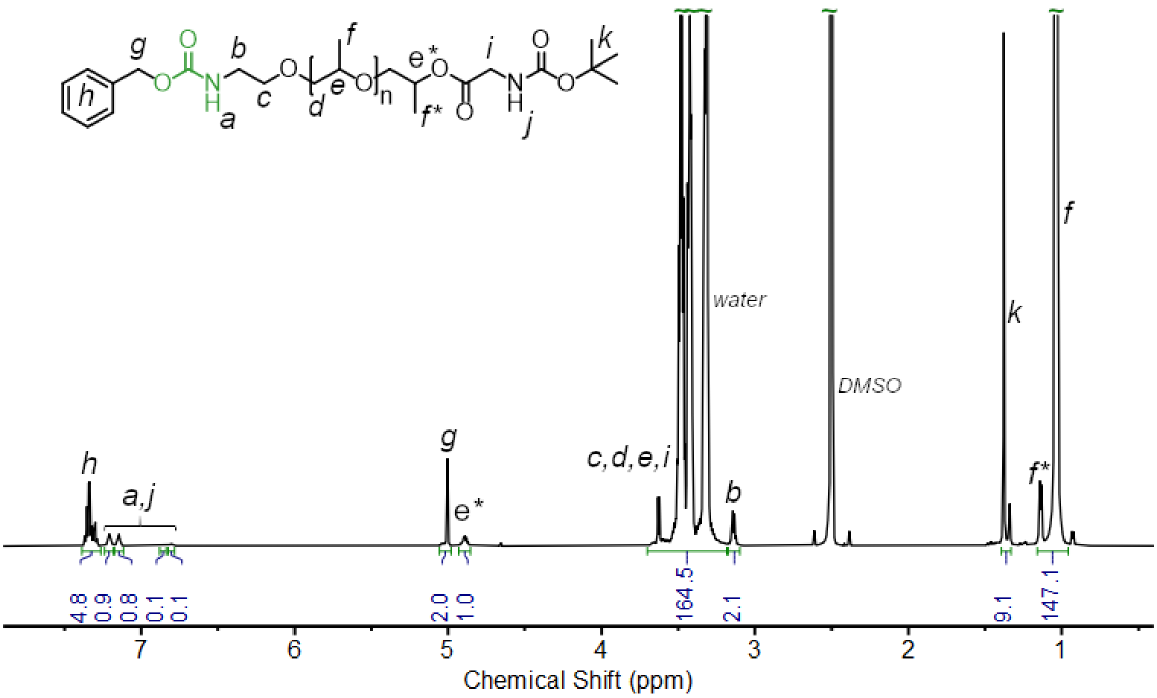

Figure S42. ^{1}H NMR spectrum of CbzEA-PPO-Boc prepared from CbzEA-PPO (Table 1, entry 5).

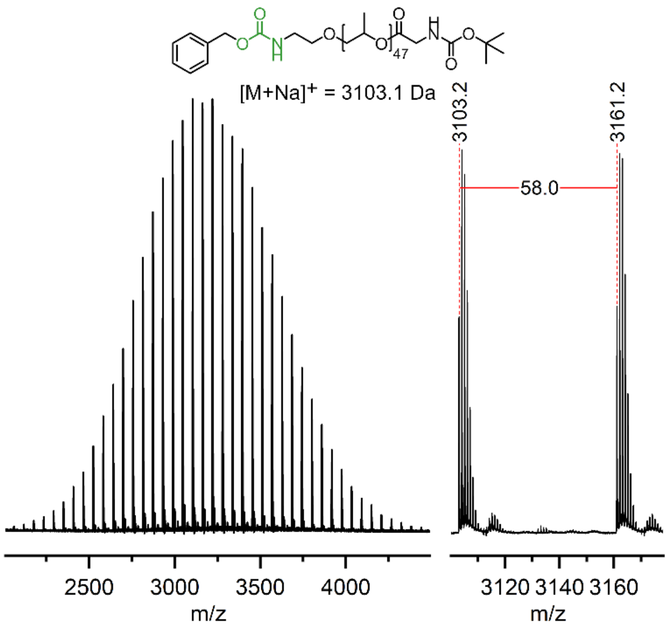

Figure S43. MALDI-TOF mass spectrum and its enlarged region with denoted measured monoisotopic signals for CbzEA-PPO-Boc prepared from CbzEA-PPO (Table 1, entry 5). Additional low intensity peak distribution (Δ = –46.9 Da) corresponds to the species formed by Boc fragmentation during MALDI-TOF MS measurements.

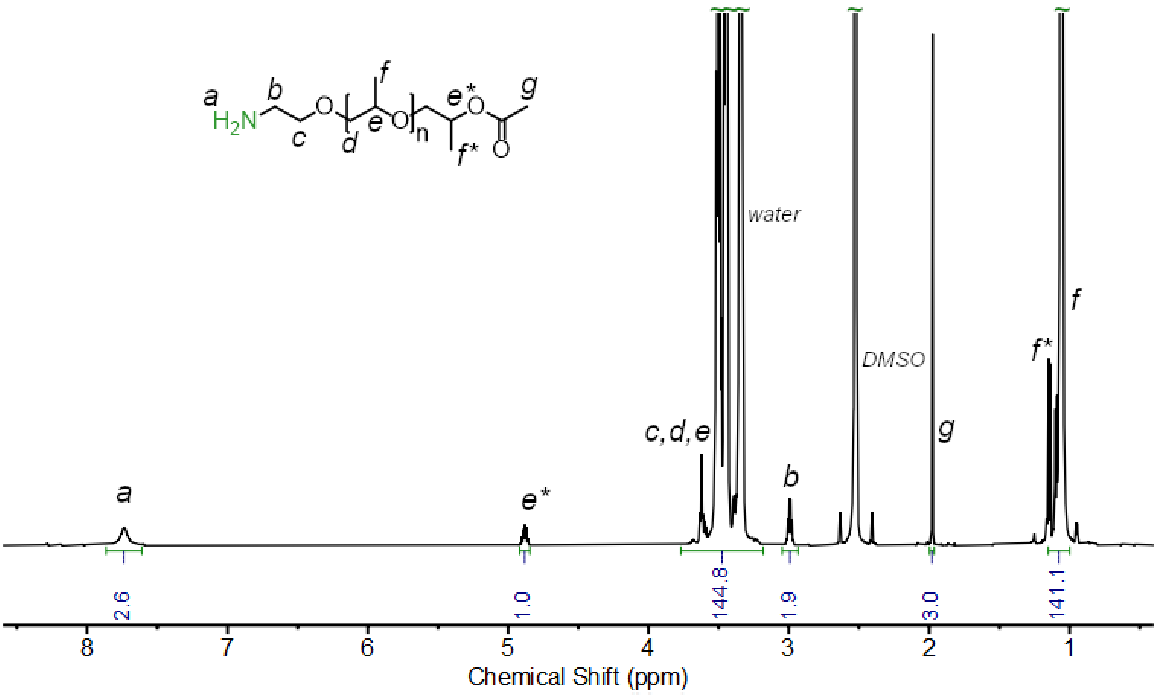

Figure S44. ^{1}H NMR spectrum of amino end-functionalized H_2N-PPO-OAc after the deprotection of BocEA-PPO-OAc prepared from BocEA-PPO (Table 1, entry 1) recorded in DMSO-d_6 with added TFA.

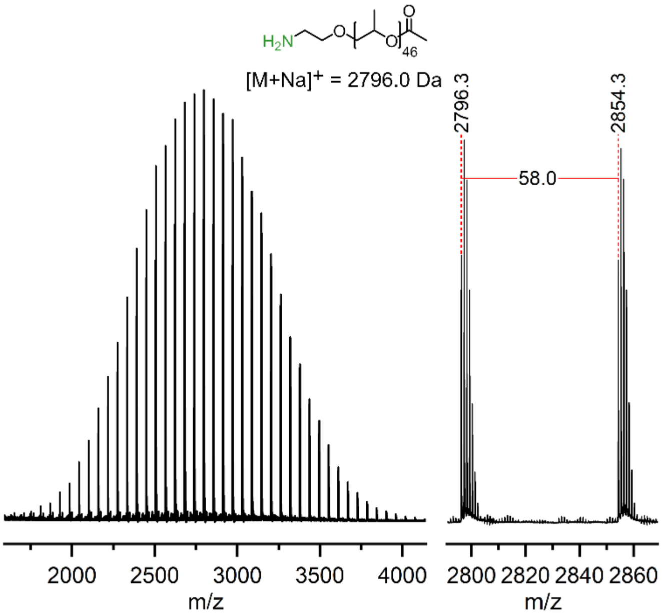

Figure S45. MALDI-TOF mass spectrum and its enlarged region with denoted measured monoisotopic signals for H_2N-PPO-OAc obtained from BocEA-PPO (Table 1, entry 1).

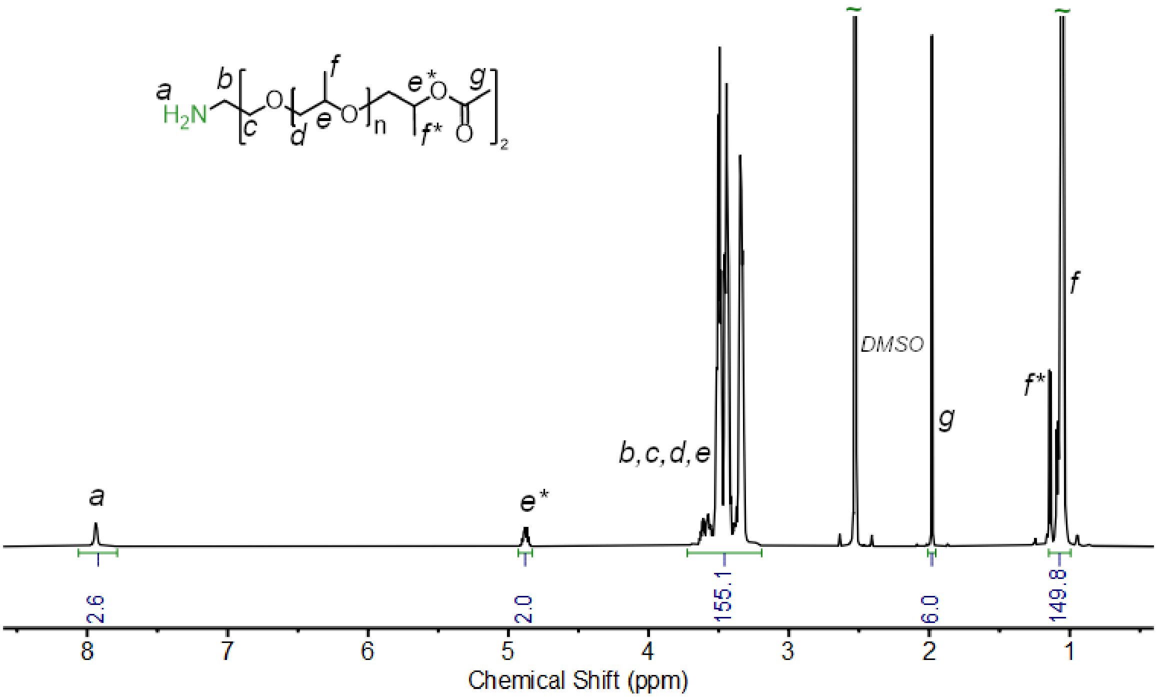

Figure S46. ^{1}H NMR spectrum of amino end-functionalized H_2N-(PPO-OAc)$_2$ after the deprotection of BocSr-(PPO-OAc)$_2$ prepared from BocSr-(PPO)$_2$ (Table 1, entry 8) recorded in DMSO-d_6 with added TFA.

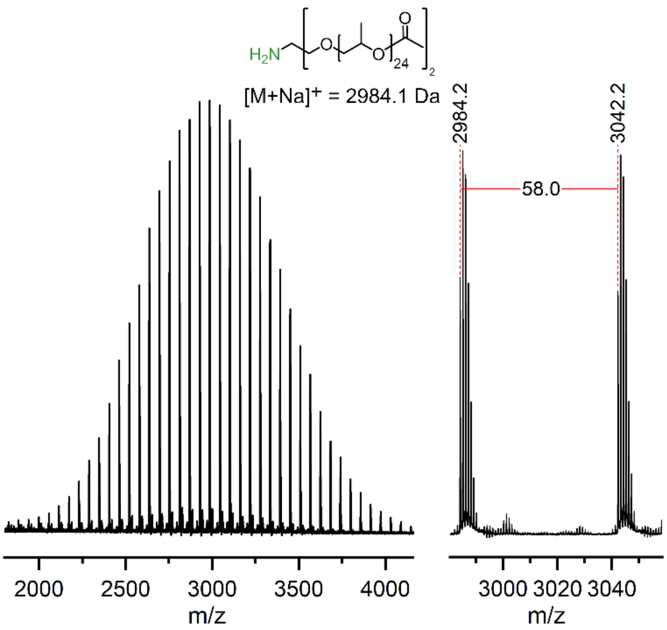

Figure S47. MALDI-TOF mass spectrum and its enlarged region with denoted measured monoisotopic signals for H_2N-(PPO-OAc)$_2$ obtained from BocSr-(PPO)$_2$ (Table 1, entry 8).

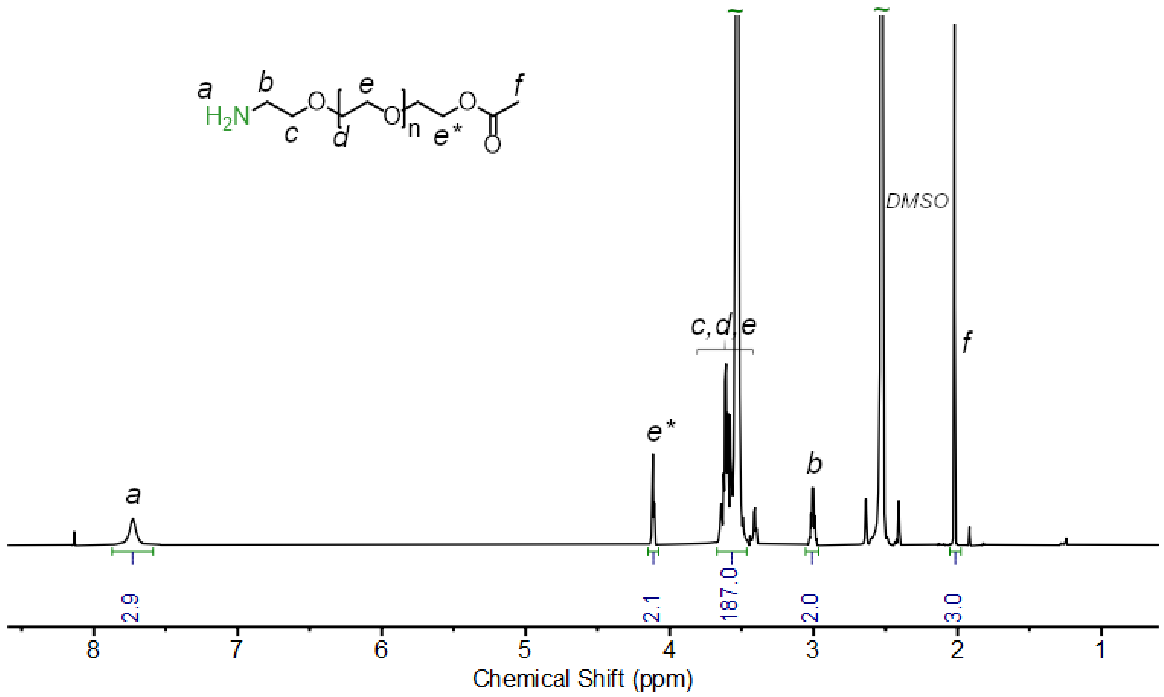

Figure S48. ^{1}H NMR spectrum of amino end-functionalized H_2N-PEO-OAc after the deprotection of BocEA-PEO-OAc prepared from BocEA-PEO (Table 1, entry 10) recorded in DMSO-d_6 with added TFA.

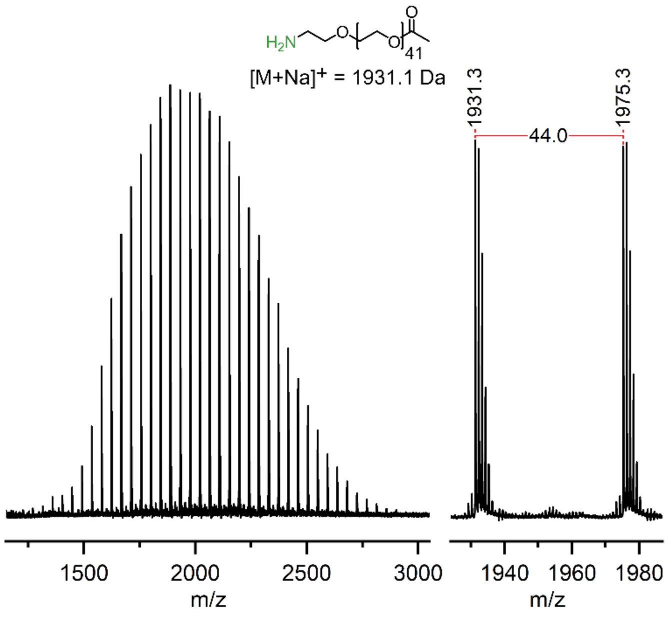

Figure S49. MALDI-TOF spectrum and its enlarged region with denoted measured monoisotopic signal for H_2N-PEO-OAc obtained from BocEA-PEO (Table 1, entry 10).

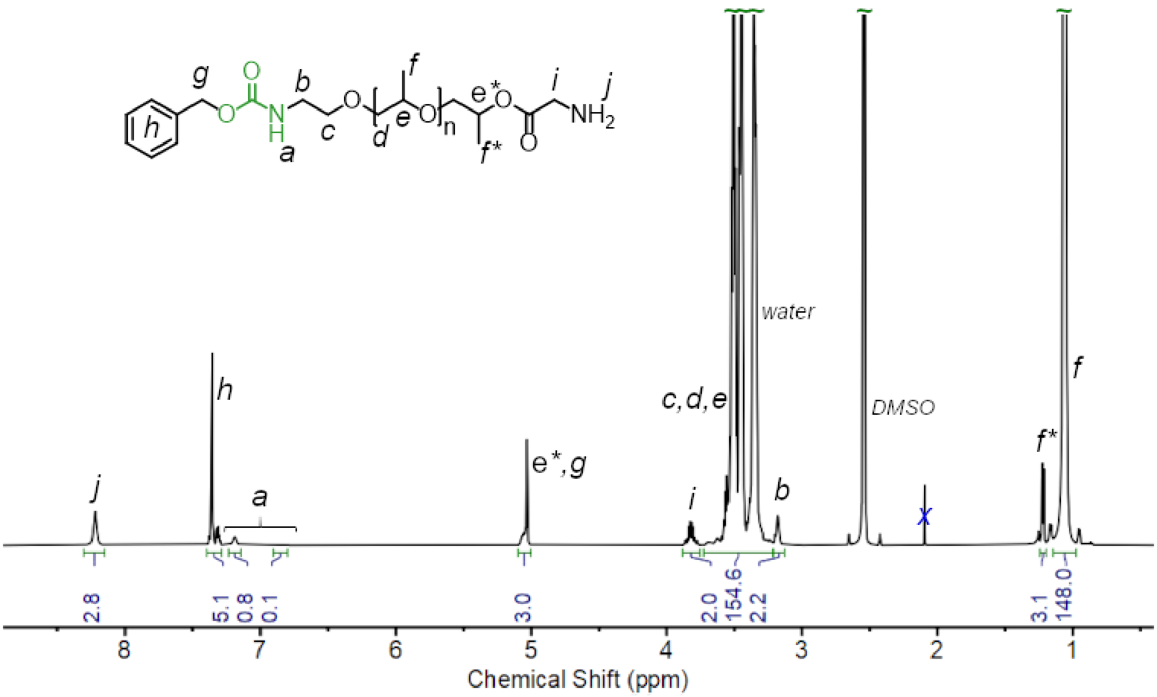

Figure S50. ^{1}H NMR spectrum of amino end-functionalized CbzEA-PPO-NH_2 after Boc deprotection of CbzEA-PPO-Boc prepared from CbzEA-PPO (Table 1, entry 5) recorded in DMSO-d_6 with added TFA.

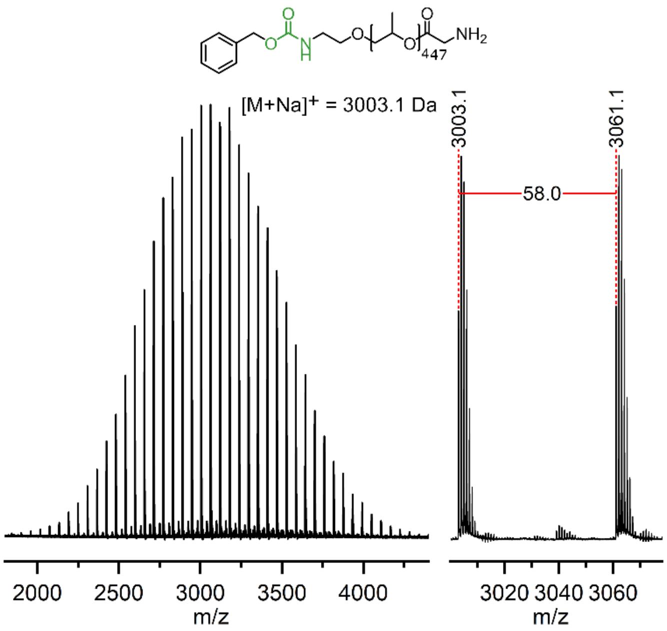

Figure S51. MALDI-TOF mass spectrum and its enlarged region with denoted measured monoisotopic signals for CbzEA-PPO-NH_2 obtained from CbzEA-PPO (Table 1, entry 5).

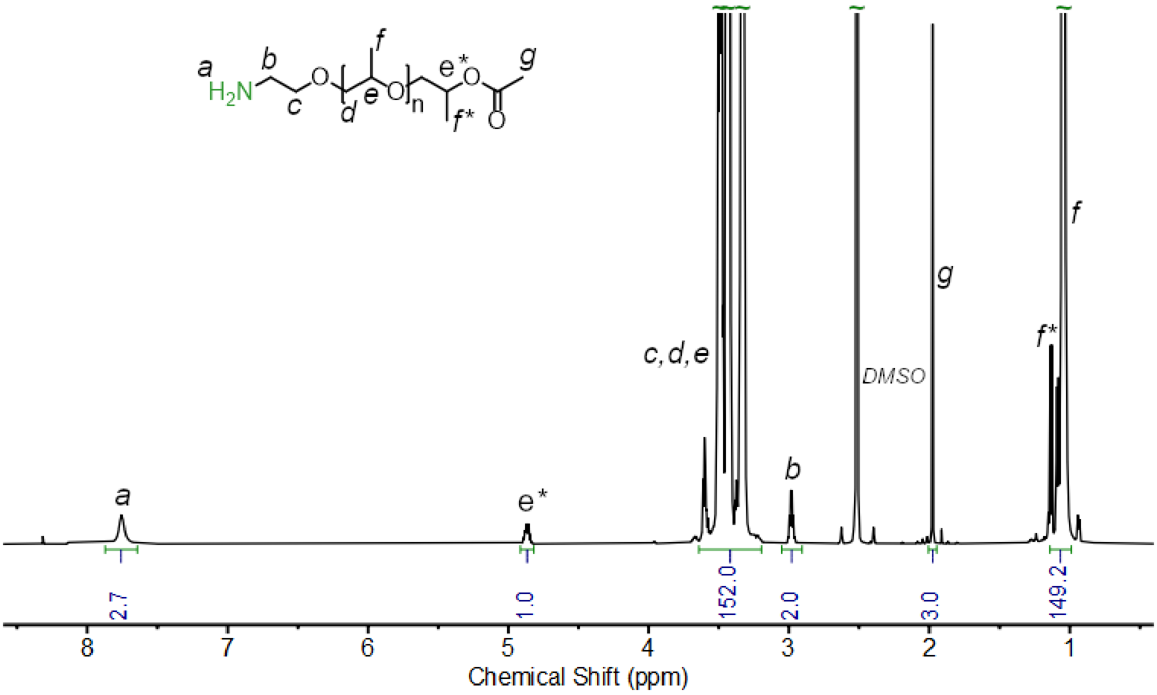

Figure S52. ^{1}H NMR spectrum of amino end-functionalized H_2N-PPO-OAc after the deprotection of CbzEA-PPO-OAc prepared from CbzEA-PPO (Table 1, entry 5) recorded in DMSO-d_6 with added TFA.

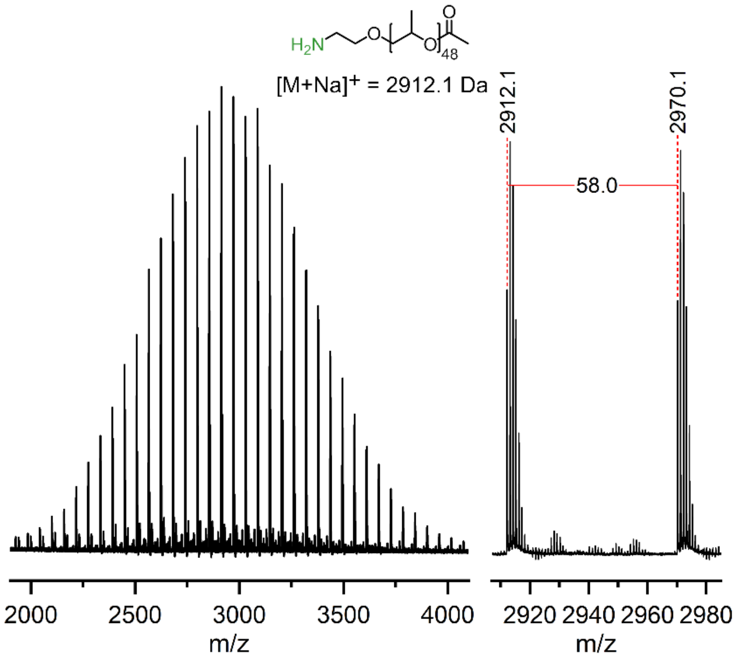

Figure S53. MALDI-TOF mass spectrum and its enlarged region with denoted measured monoisotopic signals for H_2N-PPO-OAc obtained from CbzEA-PPO (Table 1, entry 5).

S4. Mechanistic studies

Table S1. ROP of epoxides (EO, PO) with initiators containing carbamate group in the structure.

entry	init.	M[a]	$[M]_0/[init.]_0/[^tBuP_2]/[Et_3B]$	time (h)	$M_{n.th}$[b] (kg mol^{-1})	M_w[c] (kg mol^{-1})	$Đ$[d]
1	BocEA	PO	80/1/0.025/0.1	5	4.8	5.5	1.06
2	MeOH		80/1/0.025/0.1	5	4.6	5.6	1.06
3	MeOH-BocMC		80/1/0.025/0.1	5	4.6	4.7	1.15
4	BocMC		45/1/0.025/0.1	2	2.6	36.7	1.31
5	BocMC	EO	45/1/0.025/0.1	0.5	2.0	73.6	1.29
6	BocPC		45/1/0.025/0.1	0.5	2.0	40.0	1.38

[a]$[EO]_0$ = 7 M in THF or $[PO]_0$ = 14 M in bulk. [b]Calculated from the monomer to initiator feed ratio. [c]Determined by SEC-MALS. [d]Determined by SEC and column calibration with PEO standards.

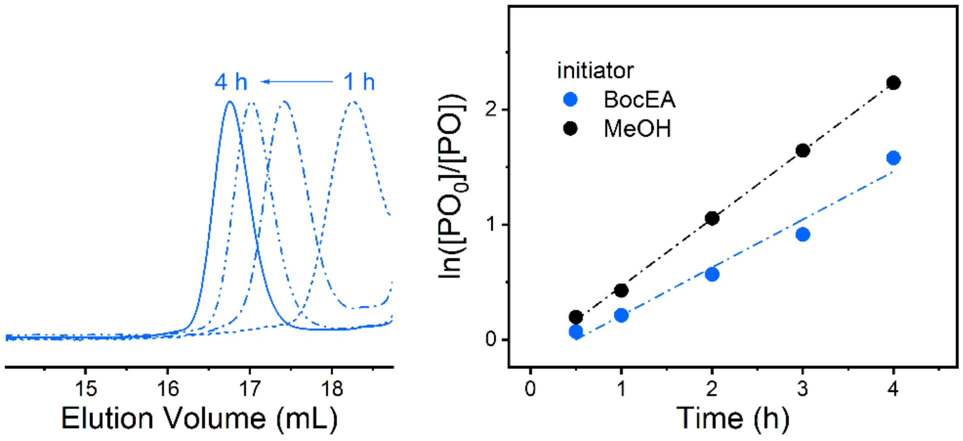

Figure S54. Left: SEC traces of the aliquots withdrawn at different reaction time from ROP of PO in bulk initiated by BocEA. Right: kinetic plots for the ROP of PO in bulk initiated by BocEA and MeOH with $[PO]_0/[init.]_0/[^tBuP_2]/[Et_3B]$ = 80/1/0.025/0.1, respectively (Table S1; entries 1 and 2).

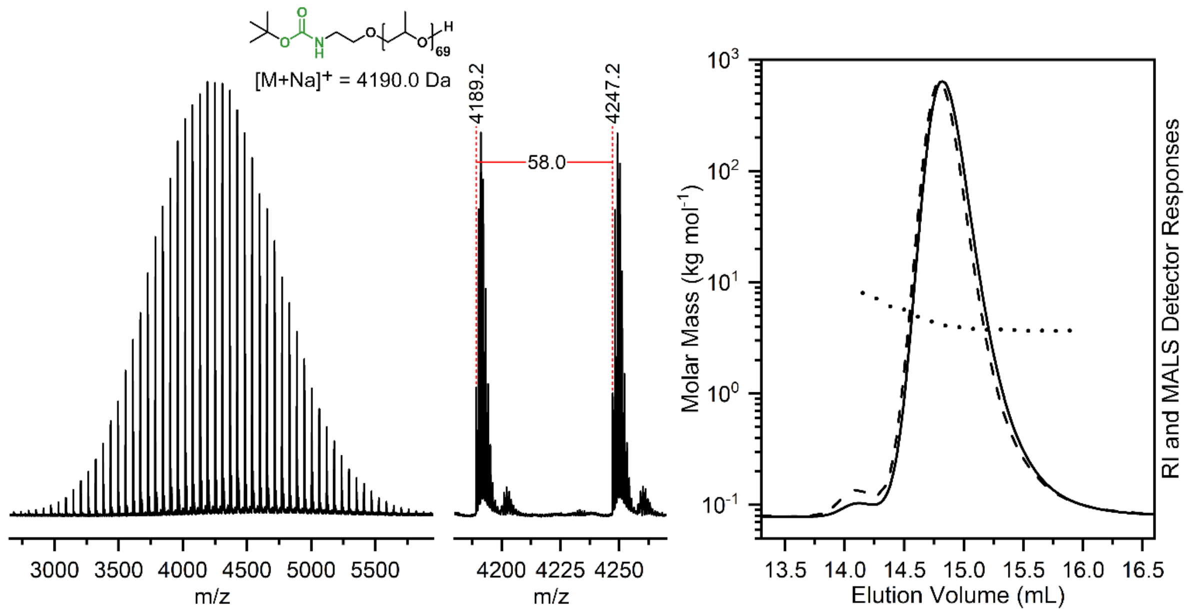

Figure S55. Left: MALDI-TOF mass spectrum and its enlarged region with denoted measured monoisotopic signal for BocEA-PPO (Table S1, entry 1). Additional, low intensity peak distribution (Δ = –46.9 Da) corresponds to the species formed by Boc fragmentation during MALDI-TOF MS measurements. The solid and dashed lines represent the RI and 90° LS detector responses, respectively, while the dotted line represents molar mass as a function of elution volume.

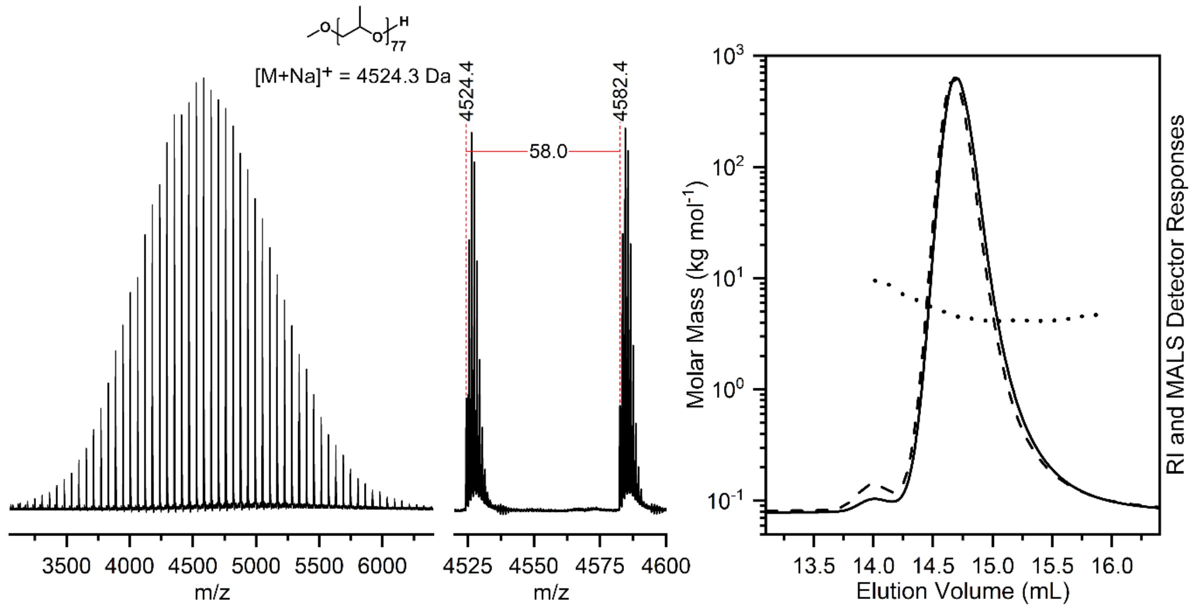

Figure S56. Left: MALDI-TOF mass spectrum and its enlarged region with denoted measured monoisotopic signals for PPO (Table S1, entry 3) prepared in the present BocMC and MeOH. Right: SEC-MALS-RI chromatogram of MeOH-PPO. The solid and dashed lines represent the RI and 90° LS detector responses, respectively, while the dotted line represents molar mass as a function of elution volume.

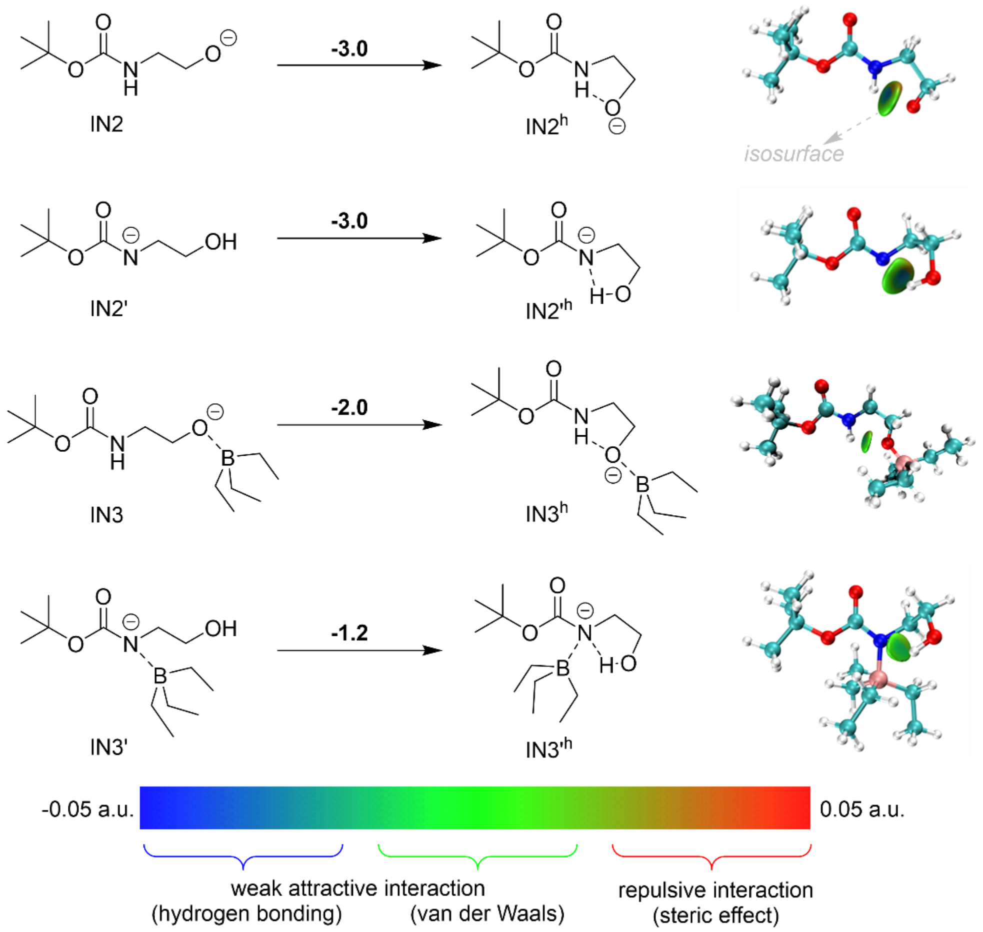

Figure S57. Formation of 5-membered cyclic structures of **IN2**, **IN2'**, **IN3**, and **IN3'** through intramolecular hydrogen bonding with DFT-calculated Gibbs free energy (Δ*G*, in kcal mol^{-1}) given above the arrows. The 3D images of optimized structures with the isosurfaces visualized by VMD are shown on the right (isovalue = 0.004 a.u.), and a color bar with interpretation is attached below.

Table S2. CVB indices calculated by Multiwfn[a]

	**IN2^{h}**	**IN2'^{h}**	**IN3^{h}**	**IN3'^{h}**
CVB index	0.004083	-0.00207	0.05079	0.05476

[a]The CVB indices are around 0 in general, with positive values indicating weaker hydrogen bonding.

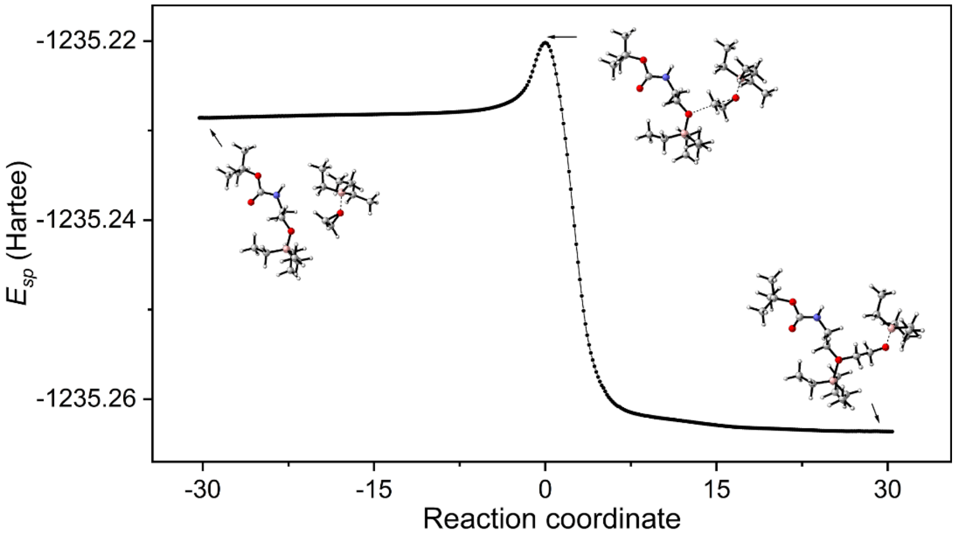

Figure S58. IRC of hydroxyl-initiated ROP of EO in Figure 4 (**IN3+AM→TS1→IN4**).

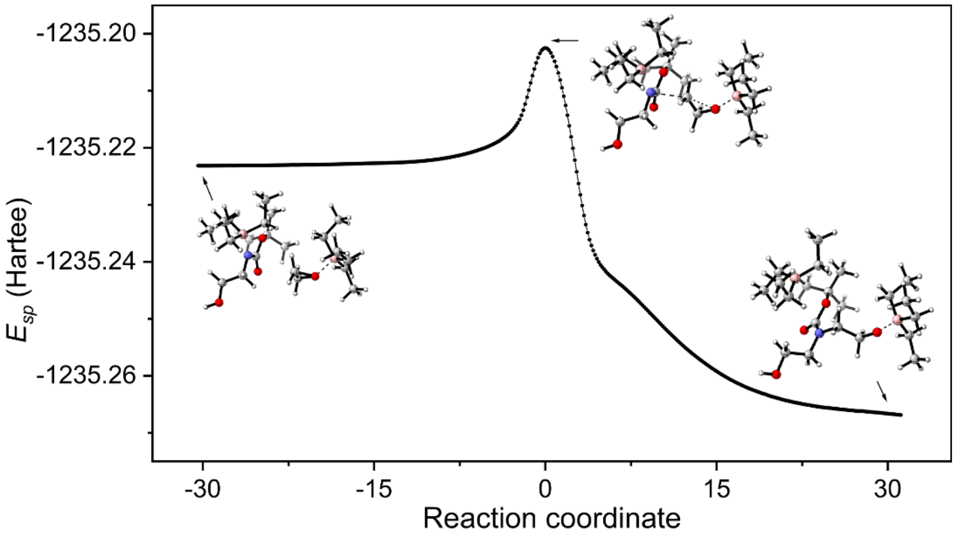

Figure S59. IRC of carbamate-initiated ROP of EO in Figure 4 (**IN3’+AM→TS1’→IN4’**).

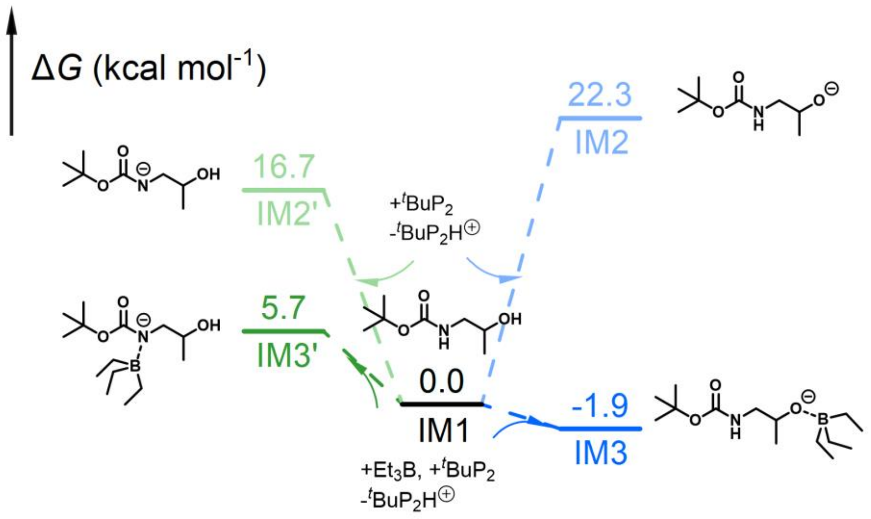

Figure S60. DFT-calculated Gibbs free energy of the intermediates, modelling the deprotonation of secondary hydroxyl and carbamate groups by ${}^tBuP_2$-Et_3B Lewis pair or by ${}^tBuP_2$ alone.

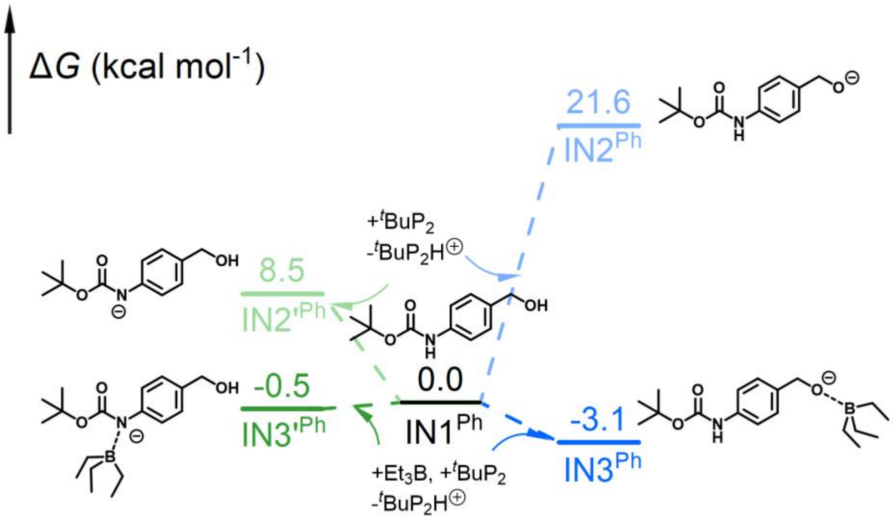

Figure S61. DFT-calculated Gibbs free energy of the intermediates for the deprotonation of BocBA by ${}^tBuP_2$-Et_3B Lewis pair or by ${}^tBuP_2$ alone.

S5. Block copolymers preparation and characterization

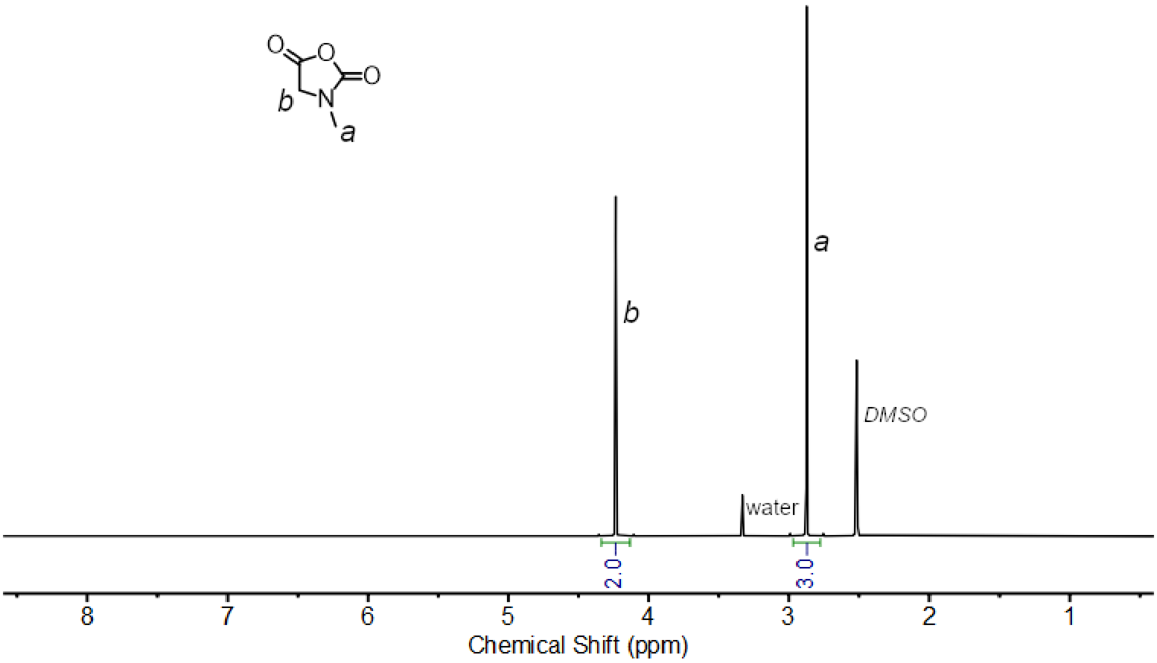

Figure S62. ^{1}H NMR spectrum of Sar NCA.

Table S3. Results for amphiphilic PSar-*b*-$(PPO)_2$ AB_2 miktoarm star prepared from amino end-functionalized PPO macroinitiator H_2N-$(PPO\text{-}OAc)_2$ obtained from BocSr-PPO_2 (Table 1, entry 8).

Block copolymer	$DP_{(Sar)th}$[a]	$M_{n.th}$[a] (kg mol^{-1})	$DP_{(Sar)}$[b]	M_n[b] (kg mol^{-1})	M_w[c] (kg mol^{-1})	$Đ$[d]
PSar-*b*-$(PPO)_2$	20	4.5	21	4.6	4.6	1.10

[a]Calculated from the monomer to initiator feed ratio. [b]Calculated from ^{1}H NMR data. [c]Determined by SEC-MALS. [d]Determined by SEC and column calibration with PEO standards.

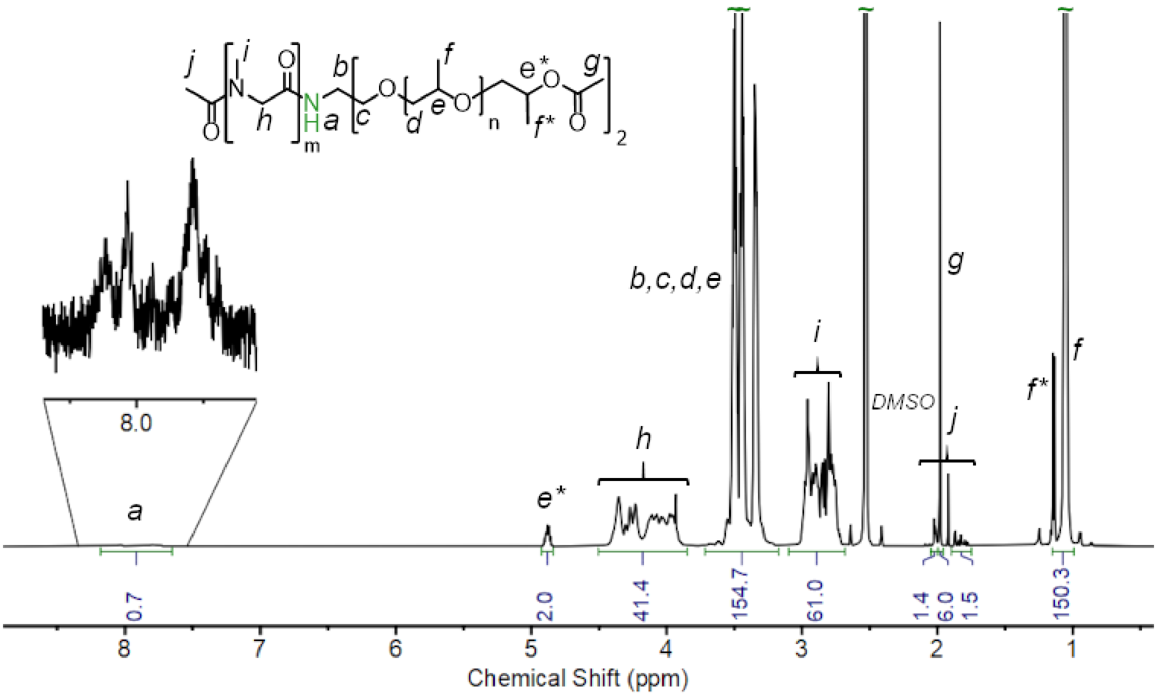

Figure S63. ^{1}H NMR spectrum of PSar-*b*-(PPO)$_2$ (Table S3) obtained from H_2N-(PPO-OAc)$_2$ recorded in DMSO-d_6 with added TFA.

S6. Computational Details

All the calculations were performed with the Gaussian16 package.[2] The optimized structures and harmonic vibrational frequencies were obtained under B3LYP-D3(BJ)/def-TZVP level[3–6] with SMD solvent model (THF).[7] The single point energy was calculated under B2PLYP-D3(BJ)/ma-def2-TZVP level,[4–6,8,9] solvation energy of SMD (THF)[7] model was obtained under M05-2X/6-31G(d) level.[10–13] Transition state structures were confirmed by intrinsic reaction coordinate (IRC)[14] calculation. All the free energies were reported in kcal mol^{-1}. Additionally, the free energies at 1 atm concentration were corrected to a 1 mol L^{-1} standard state using a +1.89 kcal mol^{-1} correction. Isosurfaces of the non-covalent interactions are calculated by Multiwfn[15] using independent gradient model based on Hirshfeld partition (IGMH) analysis. The core-valence bifurcation (CVB) indices are also calculated by Multiwfn based on the topological analysis of the electron localization function for comparison of the intensity of D-H···A hydrogen bonding, where D and A denote the donor and acceptor atoms, respectively. 3D structures were visualized by CYLview 20 and VMD.[16,17]

Cartesian coordinates (for the optimized structures)

^{t}BuP$_2$

P	-1.23666800	0.47773800	-0.11918400
N	-2.52162200	-0.29233200	-0.63036000
N	-1.56377600	1.44420300	1.24819800
N	0.09157500	-0.38769300	0.27609900
N	-0.75795100	1.56953500	-1.36822100
P	1.65866000	-0.30709500	0.10199000
N	2.13077800	-1.38790800	-1.10772200
N	2.49389700	1.08335800	-0.34439600
N	2.33220100	-0.62743300	1.62287100
C	-3.15258800	-1.46398800	-0.01784600
C	-2.43031500	-2.75379600	-0.44859000
H	-1.41824800	-2.77501800	-0.04325400
H	-2.95931600	-3.64474900	-0.09568600
H	-2.36403000	-2.80180600	-1.53799300
C	-4.59576700	-1.51419700	-0.54067800
H	-5.12996400	-2.39097300	-0.16306300
H	-5.14202900	-0.61886200	-0.23408300
H	-4.59883300	-1.55129300	-1.63270500
C	-3.19307600	-1.42870300	1.52151500
H	-3.73549400	-0.55277900	1.87944000
H	-3.69198600	-2.31945100	1.91446700
H	-2.18471200	-1.39584500	1.93854100
C	-2.87017500	2.05270500	1.43127800
H	-3.57856800	1.63041400	0.72347400
H	-3.23766400	1.86435900	2.44640000
H	-2.83930800	3.14075200	1.28532300
C	-0.53114400	2.01130400	2.09052400
H	-0.88624600	2.05172400	3.12577500
H	0.35878700	1.39027900	2.06880800
H	-0.25467300	3.03268500	1.79279800
C	-1.47811900	2.83379900	-1.46230800
H	-2.51554700	2.71419700	-1.80634200
H	-1.48595500	3.33976300	-0.49959000
H	-0.96065500	3.48113100	-2.17529600
C	-0.59715100	0.94276900	-2.67778500

H	-0.04462000	0.00909200	-2.58723300
H	-1.56116800	0.72309000	-3.15404400
H	-0.03126900	1.61036900	-3.33231100
C	2.62809400	2.20230100	0.58115900
H	3.60032300	2.67919900	0.42811800
H	1.84697700	2.95130500	0.41342400
H	2.56966900	1.86035900	1.61026100
C	2.60146400	1.49896000	-1.73824100
H	1.86818100	2.27566200	-1.97146300
H	3.60416100	1.89859900	-1.91748000
H	2.43786000	0.65711600	-2.40309000
C	3.77636100	-0.68594400	1.81508300
H	4.28550700	-0.03763200	1.10662600
H	4.01724100	-0.34261300	2.82503300
H	4.16632900	-1.70457000	1.70196900
C	1.59148800	-1.43787600	2.58206600
H	1.83333600	-2.50478800	2.48992200
H	1.84813300	-1.11690300	3.59537500
H	0.52455500	-1.30668600	2.43020500
C	3.53919600	-1.67086100	-1.35525400
H	4.15944000	-0.82135300	-1.08070700
H	3.88386000	-2.55072500	-0.79907000
H	3.68597700	-1.86570300	-2.42125100
C	1.23622100	-2.45225500	-1.53941400
H	1.39416300	-3.37920000	-0.97307900
H	0.20459700	-2.14230500	-1.41793400
H	1.41865900	-2.66389600	-2.59671000

${}^{t}BuP_2H^+$

P	-1.13515000	0.54678900	-0.09124700
N	-2.53116600	-0.19331900	-0.63631200
N	-1.52989500	1.29937400	1.33736000
N	0.07032400	-0.48146000	0.10967400
N	-0.76895400	1.70941500	-1.25575300
P	1.66418500	-0.32042700	0.05682700
N	2.20957700	-1.14296000	-1.30012800
N	2.39291900	1.17573800	-0.08090100
N	2.24369000	-0.88282800	1.53294300
C	-3.10711400	-1.47925600	-0.15606900
C	-2.37195200	-2.66334100	-0.79451000
H	-1.33100300	-2.68324400	-0.47478600
H	-2.84289600	-3.60561600	-0.50594900
H	-2.39950300	-2.59056000	-1.88441100
C	-4.57507200	-1.47884800	-0.58738700
H	-5.05933900	-2.41074200	-0.29030500
H	-5.11180700	-0.64614700	-0.12925500
H	-4.65879600	-1.38695100	-1.67359100
C	-3.01436100	-1.57023500	1.36735900
H	-3.54315000	-0.74670000	1.84671300
H	-3.47084600	-2.50515200	1.69566800
H	-1.97872300	-1.56808600	1.70886900
C	-2.80582600	1.98205000	1.52225000
H	-3.40921400	1.92036400	0.62100500
H	-3.36276700	1.51742800	2.34116000

H	-2.64576600	3.03566200	1.76852000
C	-0.70397900	1.24454400	2.53413600
H	-1.28389000	0.81887000	3.35895100
H	0.16825200	0.62070900	2.37434600
H	-0.37322400	2.24547500	2.82599800
C	-1.27689500	3.07417800	-1.16066800
H	-2.30029300	3.17048300	-1.54281800
H	-1.24717300	3.42002100	-0.13093200
H	-0.62983200	3.72387900	-1.75303700
C	-0.58517800	1.24223500	-2.62983300
H	-0.15302300	0.24305900	-2.63855800
H	-1.53048200	1.22368800	-3.18713400
H	0.10061600	1.91322300	-3.14876300
C	2.37723300	2.11376600	1.03922200
H	3.31013100	2.68224700	1.04137700
H	1.54317300	2.81789600	0.95022700
H	2.29084200	1.58525700	1.98339600
C	2.56280800	1.83681400	-1.37214900
H	1.79974500	2.60617000	-1.51494300
H	3.54819100	2.30873400	-1.40539400
H	2.49031900	1.11924200	-2.18308300
C	3.67458000	-0.85886600	1.83140100
H	4.16775300	-0.05522900	1.29080900
H	3.81046800	-0.68701300	2.90190700
H	4.15878100	-1.80644000	1.57216800
C	1.53064100	-1.95139200	2.22889900
H	1.89284600	-2.94190000	1.92891800
H	1.68791200	-1.83983800	3.30418000
H	0.46667000	-1.88854300	2.02254600
C	3.64490200	-1.32017600	-1.51263000
H	4.20691900	-0.50759600	-1.05850700
H	4.00059000	-2.26923900	-1.09732900
H	3.84983200	-1.31695700	-2.58572900
C	1.39187000	-2.16630700	-1.94144400
H	1.59443600	-3.16401900	-1.53459600
H	0.33919300	-1.94251100	-1.80778300
H	1.61740200	-2.17931100	-3.01056900
H	-2.70329600	-0.04474400	-1.62262600

EO

C	0.73095200	-0.37614200	0.00016500
H	1.26595500	-0.59440000	0.91974300
C	-0.73104900	-0.37606900	0.00015900
O	0.00008500	0.86184900	-0.00038900
H	-1.26549400	-0.59641800	-0.91921300
H	1.26552000	-0.59657900	-0.91911700
H	-1.26608200	-0.59413300	0.91975100

Et_3B

B	-0.00002700	0.00011300	0.00031800
C	-0.93835100	-1.26619200	0.00028000
H	-1.61441600	-1.16634600	0.86212000
H	-1.61479300	-1.16616600	-0.86123400
C	-0.30348700	-2.65903900	0.00000100

H	0.32694200	-2.81238600	-0.87969900
H	0.32709300	-2.81267800	0.87954100
H	-1.05985100	-3.44895300	-0.00006100
C	-2.15115400	1.59226700	0.00023500
H	-2.59882000	1.12356100	0.88043400
H	-2.45729000	2.64219800	-0.00046800
H	-2.59964900	1.12213700	-0.87877800
C	1.56574300	-0.17946900	-0.00036000
H	1.81673900	-0.81437600	-0.86275400
H	1.81779900	-0.81588900	0.86056500
C	-0.62748600	1.44582900	-0.00033000
H	-0.20314300	1.98076600	-0.86266200
H	-0.20234400	1.98223900	0.86066300
C	2.45473600	1.06662000	0.00014900
H	3.51696600	0.80637700	-0.00068000
H	2.27194100	1.69017100	-0.87883400
H	2.27295600	1.68868100	0.88040200

AM (active monomer)

C	2.21282600	-0.79454200	-0.89270600
H	3.23222100	-0.50069700	-0.67689600
C	1.59887800	-1.90323700	-0.16648400
O	1.29838200	-0.52902800	0.20519400
B	-0.30149400	0.32305000	-0.02336200
C	-1.02744300	-0.44580300	-1.23746500
H	-1.13771200	-1.51708100	-1.03355600
C	0.22482800	1.80050600	-0.38174100
H	0.80508200	1.77423300	-1.31444100
C	-0.93466000	0.12348500	1.44002000
H	-0.23780800	0.48921000	2.20292900
H	0.77296700	-2.43452800	-0.61904900
H	1.82658800	-0.52705000	-1.86748900
H	-1.80023200	0.79824700	1.50551900
C	-1.38556100	-1.29231000	1.81943300
H	-0.54756500	-1.99571800	1.81005400
H	-1.82306100	-1.33143600	2.82231600
H	-2.13621800	-1.67788300	1.12456400
H	-0.66216700	2.40113400	-0.62578500
C	1.03370500	2.54084800	0.68977700
H	1.92690700	1.97782400	0.97522000
H	1.36531000	3.52723100	0.34925500
H	0.44734300	2.69389500	1.59939600
H	-0.44230700	-0.37401600	-2.16371500
C	-2.42932200	0.12425700	-1.51362000
H	-2.38488700	1.18296200	-1.78217500
H	-2.93092300	-0.39843400	-2.33385100
H	-3.07305300	0.04188600	-0.63366900
H	2.16042100	-2.43202300	0.59334800

IN1

C	2.33106900	0.01162800	0.14346500
C	2.27026900	1.18038400	1.12126900
H	3.27349900	1.58994900	1.25503500
H	1.61763100	1.97038800	0.75673800

H	1.90887100	0.84292600	2.09500800
C	2.74630100	0.44867000	-1.25727000
H	3.77096900	0.82453700	-1.22766600
H	2.71543700	-0.39923300	-1.94471200
H	2.09819300	1.23508600	-1.63742400
C	3.26393200	-1.07445200	0.66411400
H	2.94453000	-1.41763700	1.64966500
H	3.27788700	-1.92884500	-0.01472200
H	4.27859100	-0.68214400	0.74698100
O	1.02756900	-0.67846200	0.09502700
C	-0.09163600	-0.05091300	-0.33090800
O	-0.16698800	1.11091500	-0.69221100
N	-1.13527600	-0.91978100	-0.31398200
H	-0.97383600	-1.83897200	0.06730800
C	-2.49445800	-0.50728400	-0.60452500
H	-3.03603900	-1.36472900	-1.00600600
H	-2.46814900	0.26830300	-1.36890500
C	-3.20999800	0.01810000	0.63198200
H	-3.20945000	-0.75351300	1.41148800
H	-2.67883300	0.89545000	1.01610300
O	-4.54521000	0.35371900	0.24538500
H	-4.98821500	0.75072100	1.00461800

IN2

C	-2.34968000	0.17584900	-0.02018200
C	-2.73240700	-0.75379200	1.12838000
H	-3.80918400	-0.93507100	1.10626800
H	-2.21564600	-1.70789000	1.05311400
H	-2.48474500	-0.29309500	2.08721400
C	-2.60793400	-0.45907500	-1.38398600
H	-3.68033400	-0.62269000	-1.51102000
H	-2.26836600	0.20527100	-2.18151400
H	-2.09439100	-1.41295700	-1.47935400
C	-3.09574400	1.50026400	0.09792100
H	-2.88527800	1.97655600	1.05715900
H	-2.79787700	2.18230800	-0.70041900
H	-4.17122900	1.33009700	0.02456800
O	-0.94464100	0.58282800	0.09908200
C	0.06964500	-0.32865400	0.04801000
O	-0.07782600	-1.53083600	-0.11603400
N	1.25063200	0.29819100	0.19942300
H	1.23127200	1.29176600	0.37712200
C	2.54945000	-0.36476700	0.22619200
H	2.52986500	-1.20589000	-0.46747900
H	2.76458200	-0.75641800	1.22763200
C	3.68913500	0.61135000	-0.17255300
H	3.51362800	1.52543000	0.48362400
H	3.39358900	0.98527600	-1.20049800
O	4.90808100	0.08087400	-0.08493100

IN2’

C	2.26456900	0.05789800	0.11884600
C	2.10072300	1.36093800	0.90577200
H	3.05282900	1.89618100	0.95311000

H	1.35515800	1.99919900	0.43669100
H	1.78144100	1.14253500	1.92805100
C	2.66740700	0.33085300	-1.33297100
H	3.63966400	0.83031100	-1.36665200
H	2.74924100	-0.61066600	-1.88214600
H	1.92413100	0.95530600	-1.82256700
C	3.32580500	-0.81606500	0.78816000
H	3.04571300	-1.03495100	1.82065300
H	3.43112100	-1.76333900	0.25504700
H	4.29427700	-0.31062700	0.79093000
O	1.07220300	-0.75258200	0.19544200
C	-0.17435000	-0.28254400	-0.26754400
O	-0.23228000	0.80521000	-0.88212600
N	-1.12443900	-1.14020400	0.03501100
C	-2.44439500	-0.73963800	-0.42148800
H	-3.06719300	-1.63445800	-0.54073100
H	-2.41875900	-0.22064800	-1.39001900
C	-3.13319000	0.17947800	0.57812500
H	-3.18031100	-0.31806100	1.55484600
H	-2.54480200	1.09649400	0.68604900
O	-4.46097300	0.48638700	0.10916200
H	-4.86244400	1.09651500	0.73843400

IN3

C	4.68664500	0.19165900	0.32891100
C	4.37613700	1.68052500	0.44951100
H	5.31169800	2.24266600	0.48527200
H	3.78867000	2.03285400	-0.39504300
H	3.82414500	1.87974600	1.37045800
C	5.37728000	-0.14947500	-0.98807500
H	6.35482700	0.33614800	-1.02060300
H	5.53046500	-1.22785000	-1.06809900
H	4.79262400	0.18743400	-1.84116600
C	5.53005500	-0.27401900	1.50987100
H	5.02078300	-0.06613300	2.45249100
H	5.72198900	-1.34664000	1.44771600
H	6.48750400	0.24941900	1.51146300
O	3.45566100	-0.60095800	0.47757600
C	2.42384700	-0.47992000	-0.39697000
O	2.38929300	0.28511100	-1.34617800
N	1.44866800	-1.36228800	-0.07072000
H	1.56412200	-1.86521300	0.79597400
C	0.12858500	-1.34337200	-0.68309800
H	-0.22771600	-2.37129700	-0.77799600
H	0.22694300	-0.92617000	-1.68381200
C	-0.88361900	-0.52713400	0.12019900
H	-0.90797000	-0.93032600	1.14867900
H	-0.50083000	0.50404400	0.20466500
O	-2.11941200	-0.58952700	-0.50446400
B	-3.28460700	0.21474600	0.07420900
C	-4.53378200	-0.06639300	-0.95741800
C	-2.90694700	1.82532800	0.10886400
C	-3.61835100	-0.31552800	1.60503300
H	-5.38885000	0.57996300	-0.70839300

H	-4.22960200	0.23690200	-1.96973700
C	-5.01696200	-1.52051800	-1.02100400
H	-2.13888600	2.01936300	0.87401000
H	-3.78320500	2.39943800	0.44379700
C	-2.41859800	2.41278300	-1.22069400
H	-3.68471100	-1.41400100	1.60736500
H	-2.77743100	-0.08133700	2.27565900
C	-4.89580100	0.24956000	2.24093900
H	-4.18344300	-2.19064200	-1.25053400
H	-5.43328500	-1.84828900	-0.06277300
H	-5.79236500	-1.68888700	-1.77959700
H	-3.19662600	2.36184000	-1.98887000
H	-2.11222800	3.46410000	-1.14553800
H	-1.56276100	1.84939700	-1.60485800
H	-5.07067300	-0.11646000	3.26077400
H	-4.86334300	1.34236600	2.29713800
H	-5.78275900	-0.00813600	1.65366200

IN3’

C	-3.00757700	-0.20718400	-0.10397000
C	-3.35888400	-1.63292400	-0.52325100
H	-4.43582700	-1.72515700	-0.67743000
H	-2.85098200	-1.89656800	-1.45216500
H	-3.05614900	-2.34414600	0.24712700
C	-3.37542700	0.76879300	-1.22074600
H	-4.44430300	0.69628100	-1.43550800
H	-3.13980900	1.79201100	-0.93797000
H	-2.82769700	0.52018000	-2.13247700
C	-3.70961400	0.13665500	1.20917800
H	-3.39035900	-0.55077600	1.99589200
H	-3.47965000	1.15305500	1.51892800
H	-4.79082500	0.03554100	1.08691400
O	-1.56550700	-0.26966000	0.07846800
C	-0.83388500	0.83486300	0.41576000
O	-1.37505500	1.91770100	0.67520600
N	0.48831100	0.59056600	0.43748500
C	1.26686800	1.74316300	0.88735800
H	2.20411100	1.39680800	1.31491200
H	0.72211200	2.28646600	1.66148900
C	1.56677900	2.71189500	-0.24596200
H	2.10706900	2.19403100	-1.04483200
H	0.62655100	3.09486800	-0.65330900
O	2.36325200	3.78694600	0.28251400
H	2.51890900	4.41728200	-0.43006100
B	1.21993900	-0.84715800	0.14193600
C	0.69896300	-1.47304100	-1.28657200
C	0.82203600	-1.81604400	1.41684600
C	2.85980900	-0.58080300	0.07528600
H	1.31157700	-2.35075900	-1.53075600
H	-0.32258800	-1.84877800	-1.18541700
C	0.74466200	-0.51022500	-2.47842300
H	-0.27193700	-1.86859700	1.47209800
H	1.13763400	-1.31965400	2.34861000
C	1.37194900	-3.24879800	1.43278200

H	3.08497500	0.36924500	-0.42439600
H	3.26099300	-0.46198600	1.09300400
C	3.70562000	-1.64987100	-0.63525600
H	0.10233300	0.35769200	-2.30340400
H	1.75592000	-0.12460500	-2.64799500
H	0.41661800	-0.97130200	-3.41889000
H	2.46108000	-3.26522900	1.52832800
H	0.97352200	-3.84421000	2.26412600
H	1.12476700	-3.78480600	0.51062700
H	4.77953700	-1.43809900	-0.56184300
H	3.54916900	-2.64972600	-0.22486800
H	3.46605300	-1.70599600	-1.70048500

TS1

C	5.48144500	-1.87919800	0.11428700
C	6.03461300	-0.81541000	1.05761600
H	7.10820500	-0.96904800	1.18497200
H	5.86938000	0.18627000	0.66756000
H	5.56124200	-0.89486900	2.03855800
C	6.04510400	-1.74972000	-1.29735700
H	7.12002200	-1.94085300	-1.27591300
H	5.58239600	-2.48441700	-1.95976200
H	5.87619600	-0.75453200	-1.70236100
C	5.74582000	-3.27376600	0.66895100
H	5.31001200	-3.38203800	1.66367600
H	5.31642500	-4.03606700	0.01654100
H	6.82076300	-3.44635600	0.74173200
O	4.01048100	-1.81691400	0.08346300
C	3.35680800	-0.71436400	-0.36051200
O	3.88499400	0.31089700	-0.75676500
N	2.01959200	-0.93284400	-0.32013700
H	1.70868400	-1.78923500	0.11300400
C	1.04089100	0.12022900	-0.53787100
H	0.15533600	-0.32314800	-0.99406900
H	1.45949300	0.83539600	-1.24120200
C	0.64778300	0.81310800	0.77540000
H	0.43280500	0.02559200	1.51238500
H	1.50913900	1.36699800	1.16227100
B	-0.43837600	3.10963800	0.14863700
C	0.90417000	3.88348500	0.71555400
H	0.66469800	4.95441400	0.78760500
H	1.09005200	3.57504500	1.75446300
C	2.21661800	3.75839800	-0.07367800
H	2.10897100	4.15099200	-1.08882600
H	3.03991900	4.31245400	0.39460400
H	2.55041400	2.72332400	-0.17539700
C	-1.79308000	3.78195000	0.79102000
H	-2.68672300	3.27990200	0.39058600
C	-0.51149800	3.10186000	-1.49376500
H	0.39171800	2.64133000	-1.91476800
H	-1.33838800	2.45417900	-1.81789800
C	-0.69527900	4.47818500	-2.14880900
H	-1.65052500	4.93023300	-1.86661800
H	0.08785300	5.17800900	-1.84026300

H	-0.67297600	4.43626000	-3.24454800
O	-0.47698200	1.63451900	0.66375200
H	-1.87923000	4.82251500	0.45038600
C	-1.86996900	3.76969300	2.32270900
H	-1.07776400	4.38173800	2.76513600
H	-2.82251000	4.15161200	2.71006200
H	-1.73876800	2.75639500	2.71362100
C	-2.80038000	0.35432400	-0.70545000
C	-2.26532400	0.42584700	0.63735100
O	-3.67949800	-0.46455500	0.07831700
H	-2.68293600	1.12377300	1.33842900
H	-1.61647100	-0.35267200	0.98845500
B	-3.72403700	-2.06681500	-0.15810700
C	-4.38336400	-2.24357600	-1.64128200
H	-4.47900800	-3.31722000	-1.85193900
C	-4.68933500	-2.58206200	1.04594600
C	-2.21061200	-2.67925600	-0.08739800
H	-1.73855300	-2.45012600	0.87676400
H	-4.95575800	-3.63278200	0.86394100
H	-3.68664200	-1.86978200	-2.40662400
H	-5.64094100	-2.03626600	1.00735800
C	-5.74943100	-1.58244900	-1.86252300
H	-6.11805600	-1.70807700	-2.88707700
H	-6.50857900	-1.99987900	-1.19480900
H	-5.70635000	-0.50798700	-1.66172000
C	-4.11880500	-2.45741000	2.46424800
H	-3.84365200	-1.42237500	2.68846500
H	-4.82940900	-2.77593800	3.23564400
H	-2.22320600	-0.18313400	-1.45069100
H	-3.21631800	-3.06263800	2.59090400
H	-1.56278700	-2.21858400	-0.84700400
C	-2.15315100	-4.20107000	-0.28249700
H	-1.13356200	-4.59526300	-0.20787600
H	-2.75584200	-4.72174100	0.46748200
H	-2.53986300	-4.49443500	-1.26225400
H	-3.29410200	1.23443800	-1.10645100

TS1’

C	1.54231000	-0.49632600	-1.45027600
C	0.76527400	-1.05143500	-0.34742900
O	2.56116200	-1.23732000	-0.78424700
H	0.44523900	-2.07738500	-0.39732500
H	0.80879300	-0.56095900	0.60347900
B	3.75840700	-0.47097600	-0.03165900
C	4.56361800	0.33682900	-1.20438900
H	5.42766200	0.83597900	-0.74519800
C	4.61981100	-1.69643100	0.60981800
C	3.14028900	0.54372600	1.08784400
H	2.51548700	-0.00582000	1.80320300
H	5.57773200	-1.30562000	0.98152000
H	3.94158900	1.15747800	-1.59227300
H	4.89009100	-2.40532800	-0.18405300
C	5.06049000	-0.49891900	-2.39063000
H	5.56410700	0.10644300	-3.15327700

H	5.77086800	-1.26627200	-2.06974300
H	4.23375600	-1.01934100	-2.88331000
C	3.93324900	-2.47478900	1.73910500
H	2.96679900	-2.86987400	1.41224500
H	4.52882500	-3.32496600	2.09190600
H	1.69163300	0.57923100	-1.44337700
H	3.73855200	-1.83803600	2.60700800
H	2.46720700	1.27448400	0.61977000
C	4.19608000	1.32177700	1.88452800
H	3.75097800	1.97232500	2.64575100
H	4.88434800	0.64582200	2.40100500
H	4.80264400	1.95777400	1.23345900
H	1.34251800	-0.87199200	-2.45083600
C	-0.71104000	3.04054100	0.08492700
C	-0.43398000	3.42924500	1.53309600
H	-0.28942500	4.50837400	1.60848000
H	-1.26900100	3.14370700	2.17414300
H	0.46679800	2.93237300	1.89673100
C	-2.00546400	3.68403300	-0.40397400
H	-1.91672400	4.77050900	-0.33888500
H	-2.21564100	3.41407800	-1.43677200
H	-2.84413900	3.37170300	0.22061000
C	0.48042900	3.40519100	-0.79617600
H	1.38094200	2.89815000	-0.44595900
H	0.30295200	3.13474800	-1.83389400
H	0.65517400	4.48176100	-0.73626300
O	-0.87371900	1.58508900	0.17349900
C	-1.19044300	0.83386100	-0.89938900
O	-1.19449700	1.26559100	-2.04556500
N	-1.43482400	-0.47722400	-0.54105900
C	-1.68779500	-1.32736900	-1.72076200
H	-1.57613800	-2.36373700	-1.41415400
H	-0.93108600	-1.12159200	-2.47677800
C	-3.03603000	-1.17494400	-2.40862500
H	-3.85154700	-1.45616000	-1.74300100
H	-3.18039400	-0.14278100	-2.73276600
O	-2.99959400	-2.05761600	-3.54583300
H	-3.85106100	-1.99312400	-3.99337000
B	-2.18724700	-0.84504700	0.94525200
C	-3.40757600	0.22187500	1.21505500
C	-1.07558700	-0.72663200	2.16496500
C	-2.72070000	-2.39445200	0.80620300
H	-3.91946900	-0.10418800	2.12953400
H	-2.98070900	1.19764000	1.47007800
C	-4.47967400	0.44113200	0.14328000
H	-0.37932100	0.08617700	1.94668300
H	-0.46451500	-1.64024500	2.21179500
C	-1.61343300	-0.44749900	3.57856400
H	-3.43325800	-2.50782800	-0.01922000
H	-1.87957300	-3.05465000	0.55000000
C	-3.38682400	-2.97962700	2.05937700
H	-4.05751000	0.84571700	-0.77980900
H	-4.99076900	-0.49023400	-0.11790600
H	-5.25273300	1.14648700	0.47155600

H	-2.34731800	-1.18613200	3.90746800
H	-0.80912100	-0.44053100	4.32392900
H	-2.10152000	0.52917300	3.63090500
H	-3.78294300	-3.98591800	1.88005800
H	-2.68455700	-3.05895000	2.89221100
H	-4.22308400	-2.36245700	2.40059400

IN4

C	-6.58136700	0.03258500	0.18165400
C	-6.56995200	-1.39307600	0.72372800
H	-7.59419000	-1.76824300	0.77302700
H	-5.98679900	-2.05536000	0.08795900
H	-6.15207900	-1.41253200	1.73247800
C	-7.07506100	0.09999400	-1.25990500
H	-8.12151200	-0.20958500	-1.29692000
H	-7.00971800	1.12288600	-1.63673700
H	-6.49580900	-0.55249900	-1.90920900
C	-7.41847200	0.93830600	1.07637900
H	-7.04285300	0.92239900	2.10093600
H	-7.39545900	1.96718900	0.71318400
H	-8.45476400	0.59697600	1.08273700
O	-5.23648100	0.62705300	0.28782900
C	-4.17709400	0.10741400	-0.37614500
O	-4.20171500	-0.86989100	-1.10461100
N	-3.07286400	0.85555900	-0.12600500
H	-3.15616300	1.59414400	0.55528300
C	-1.74927700	0.47236500	-0.58344000
H	-1.16306600	1.38146100	-0.71500400
H	-1.84214600	-0.01529900	-1.55288200
C	-1.06201800	-0.47777300	0.40267100
H	-1.01714700	-0.00756000	1.39457200
H	-1.65497300	-1.39044600	0.48840900
O	0.23053800	-0.87105100	-0.02170300
C	1.24478700	0.10817100	0.21172800
H	1.25254400	0.38878300	1.27349100
H	1.06159300	1.01484800	-0.37621400
C	2.59439000	-0.47142500	-0.17601900
H	2.76413900	-1.37738800	0.42554600
H	2.53447100	-0.80364700	-1.22597100
O	3.56259400	0.50730900	0.01987600
B	5.05850800	0.18695500	-0.08753600
C	5.65141100	-0.19244200	1.40957500
C	5.75755800	1.57949700	-0.63013700
C	5.31832800	-1.02764600	-1.17579600
H	6.75091600	-0.24062900	1.37131500
H	5.42818000	0.63945400	2.09277100
C	5.13462100	-1.48362400	2.05867400
H	5.40153600	1.77095900	-1.65512400
H	6.84676100	1.45437500	-0.72050400
C	5.48432400	2.83200000	0.21207000
H	4.76173400	-1.93346800	-0.89633700
H	4.90497500	-0.73267400	-2.15320800
C	6.78612900	-1.42801800	-1.37756100
H	4.05344300	-1.43775400	2.21832400

H	5.32479600	-2.35843800	1.42815600
H	5.59158600	-1.68911100	3.03570200
H	5.92029000	2.74642300	1.21270500
H	5.88898400	3.75034800	-0.23294400
H	4.40851100	2.97657700	0.34459600
H	6.91532100	-2.22657300	-2.11882900
H	7.39117200	-0.58023800	-1.71322200
H	7.23391400	-1.78438300	-0.44435800

IN4’

C	-2.82426900	-2.22604000	-0.13276400
C	-2.86773900	-2.10305100	-1.65262700
H	-3.24341000	-3.03534400	-2.07913400
H	-3.51824300	-1.28962300	-1.96666500
H	-1.86513700	-1.92830500	-2.04791400
C	-4.21313400	-2.39669200	0.47442100
H	-4.63951200	-3.34555000	0.14174500
H	-4.14987100	-2.41620700	1.56455100
H	-4.87698600	-1.58893900	0.17502800
C	-1.91175300	-3.37362700	0.28357800
H	-0.90669200	-3.23055800	-0.11598500
H	-1.84553500	-3.43711100	1.37108200
H	-2.30606400	-4.31803500	-0.09522300
O	-2.15194600	-1.05616200	0.45099900
C	-2.60206700	0.20135700	0.24910800
O	-3.66535800	0.48658200	-0.28613300
N	-1.72635300	1.11674900	0.74658900
C	-2.08300100	2.52627100	0.66070000
H	-1.67953300	3.03570200	1.53707700
H	-3.16788000	2.61271500	0.67996600
C	-1.56133200	3.19555500	-0.60303800
H	-0.46735900	3.18802600	-0.61223700
H	-1.91854000	2.64540800	-1.48027800
O	-2.05616400	4.54045000	-0.60457000
H	-1.67252300	4.99784900	-1.36195800
C	-0.40842900	0.74207300	1.27062000
H	-0.06117300	1.56773300	1.89245400
H	-0.50607600	-0.13666500	1.90573400
C	0.63178400	0.45415700	0.18727200
H	0.76523900	1.36108200	-0.42341200
H	0.22399300	-0.31576000	-0.48468800
O	1.81224200	0.05417800	0.79939700
B	3.05472900	-0.13959000	-0.06641000
C	4.22752700	-0.61483100	0.98436500
C	3.46799000	1.29012800	-0.79472400
C	2.75787600	-1.28859700	-1.21866000
H	5.20295200	-0.65214500	0.47680900
H	4.33815000	0.15488400	1.76197800
C	3.98271600	-1.95955100	1.67979600
H	2.75290600	1.52808400	-1.59810900
H	4.43353400	1.17676200	-1.30881800
C	3.55440000	2.50086300	0.14255400
H	2.30945600	-2.17546600	-0.74602000
H	1.99254600	-0.92558000	-1.92154300

C	3.97398100	-1.73718400	-2.03960000
H	3.01654800	-1.95571800	2.19248400
H	3.95735600	-2.78500800	0.96091200
H	4.74892400	-2.20915100	2.42528000
H	4.33431400	2.36529900	0.89853300
H	3.77455800	3.44133900	-0.37886400
H	2.61462900	2.63906900	0.68585600
H	3.72891400	-2.48965100	-2.80001500
H	4.43327900	-0.89339800	-2.56483300
H	4.75056800	-2.17039900	-1.40139900

TS2

C	-2.73453700	-0.99178900	1.52493800
C	-2.47128900	-1.64816100	0.26714800
O	-3.81431000	-0.87750100	0.55262200
H	-2.22649800	-0.06316000	1.74755200
H	-2.69334000	-2.69187100	0.13631100
H	-1.90728900	-1.13948800	-0.49222600
B	-4.25929000	0.52136900	-0.22839300
C	-4.75301300	-0.03380200	-1.67291800
H	-5.04966400	0.82612100	-2.28841900
H	-3.89794800	-0.47885900	-2.20233000
C	-5.90548000	-1.04580300	-1.66728700
H	-6.81894600	-0.61089300	-1.25177200
H	-6.15018900	-1.40797200	-2.67218400
H	-5.66109300	-1.92005400	-1.05721900
C	-5.45664600	1.09541200	0.70708600
H	-5.96708200	1.89501100	0.15164500
H	-6.21891900	0.31897300	0.84894100
C	-5.05418600	1.63727500	2.08485700
H	-4.58818200	0.86132700	2.69970900
H	-4.33283200	2.45515600	2.00228300
H	-5.91078700	2.01953300	2.65141900
O	-0.28325800	-2.57189500	0.61018100
C	-2.96862500	1.50435800	-0.34734500
H	-2.16945200	1.03215000	-0.93420700
H	-2.52425800	1.71385400	0.63352200
C	-3.30186100	2.85391500	-1.00162100
H	-2.42502400	3.50568900	-1.07934500
H	-3.69634600	2.72296000	-2.01309700
H	-4.05944600	3.39862300	-0.43088900
H	-3.01530200	-1.58269300	2.38908300
C	0.61690500	-1.57023000	0.58710400
H	1.32504100	-1.56007800	1.45818900
H	0.16214000	-0.53778900	0.60869300
C	1.51531500	-1.58819800	-0.67473100
H	0.87539300	-1.55686900	-1.56103100
H	2.08425300	-2.51639700	-0.70025900
N	2.46024800	-0.47835800	-0.74635400
H	2.08271900	0.45282500	-0.83788900
C	3.74453900	-0.56538900	-0.33185300
O	4.33166300	-1.60008300	-0.05768800
O	4.29580200	0.67939000	-0.29540600
C	5.70556100	0.88533300	0.06335800

C	5.95837400	0.43052100	1.49784400
C	6.61750000	0.17748000	-0.93485000
C	5.86484700	2.39708700	-0.05137000
H	5.25329500	0.91302800	2.17801400
H	5.85636100	-0.64793100	1.59402200
H	6.96935700	0.71603000	1.79647300
H	6.36662500	0.47564300	-1.95517800
H	7.65440500	0.46144300	-0.74287200
H	6.53047800	-0.90351400	-0.85311300
H	6.88757400	2.68371100	0.19913500
H	5.65117400	2.73097000	-1.06831400
H	5.18490100	2.90885000	0.63196000

TS2’

C	0.71487300	1.51538100	0.65446000
C	0.32320500	1.13549600	-0.68349100
O	1.88435400	1.45045900	-0.18931300
H	0.10660800	1.88828300	-1.41991700
H	0.25457100	0.09664100	-0.94481400
B	3.06486700	0.34302600	0.07954200
C	3.69902700	0.78812800	1.51294400
H	4.51974100	0.09845400	1.75193900
C	4.06328200	0.57931700	-1.18054200
C	2.39574000	-1.13898400	0.12887500
H	1.85416400	-1.35430500	-0.79976600
H	4.99664500	0.02908900	-0.99500400
H	2.95986800	0.62960300	2.31222500
H	4.35920000	1.63562300	-1.22086900
C	4.22769000	2.22439100	1.61404900
H	4.60766600	2.46392600	2.61376300
H	5.04528800	2.40038600	0.90925100
H	3.44545000	2.95277800	1.38067900
C	3.51958100	0.17561500	-2.55683400
H	2.59042900	0.70593400	-2.78632000
H	4.22335200	0.39254300	-3.36863300
H	0.67162000	0.75687500	1.42818400
H	3.29505700	-0.89379500	-2.60387600
H	1.64115000	-1.20038800	0.92419300
C	3.41068200	-2.26810100	0.35416500
H	2.93793500	-3.25621700	0.37367200
H	4.16776600	-2.28881600	-0.43551400
H	3.94190600	-2.14753700	1.30249900
H	0.50539300	2.51951200	1.00913400
C	-1.81521700	-2.60124100	0.05496800
C	-0.92322400	-3.42854600	-0.86926600
H	-0.95268800	-4.48204800	-0.58308300
H	-1.25935300	-3.34146500	-1.90444700
H	0.11037400	-3.08324700	-0.81273400
C	-3.26404600	-3.07887700	-0.05723700
H	-3.33111200	-4.13582000	0.21308500
H	-3.91127300	-2.50183300	0.59927100
H	-3.61751300	-2.96777800	-1.08529700
C	-1.29982800	-2.70190700	1.49180900
H	-0.27695700	-2.32355400	1.54926600

H	-1.92615200	-2.12464800	2.16766700
H	-1.29376600	-3.74679600	1.81254000
O	-1.67140600	-1.25493000	-0.46393200
C	-2.33595000	-0.16632300	0.11625200
O	-3.06585100	-0.32963900	1.11085600
N	-2.03291100	0.94229400	-0.53746800
C	-2.61621100	2.15184900	0.00872700
H	-1.97750700	3.00183400	-0.26053000
H	-2.69158900	2.12663500	1.10406900
C	-4.00381800	2.41723600	-0.55593600
H	-3.95008300	2.43521200	-1.65135400
H	-4.67783500	1.60785900	-0.25705000
O	-4.47921300	3.68033900	-0.05515200
H	-5.38011400	3.80543200	-0.37499100

IN2^{h}

C	2.18698700	0.25517900	0.00935300
C	2.57750000	-0.37059700	-1.32748900
H	3.66614000	-0.41794900	-1.40420100
H	2.17430300	-1.37603600	-1.42395500
H	2.20449400	0.23908300	-2.15336200
C	2.62517000	-0.60305700	1.19374800
H	3.71590300	-0.65211200	1.22449000
H	2.27793600	-0.16107100	2.13026900
H	2.23133600	-1.61391600	1.11562000
C	2.77312300	1.65805800	0.12795500
H	2.43159000	2.28795600	-0.69533400
H	2.46953400	2.12320500	1.06750600
H	3.86325900	1.61201600	0.09997300
O	0.74379200	0.50099300	0.05956900
C	-0.15886600	-0.52292800	0.00996900
O	0.13758800	-1.70301000	-0.13393600
N	-1.40270600	-0.04478600	0.16365900
C	-2.61093900	-0.80924300	-0.07898500
H	-2.65516600	-1.12482900	-1.13008300
C	-3.77717900	0.17696200	0.23307900
H	-3.88814500	0.15579900	1.35544700
H	-4.70201200	-0.33714600	-0.14038600
O	-3.55822200	1.40495600	-0.27552300
H	-2.63993900	-1.70299400	0.55118200
H	-1.61032800	0.95174200	0.05055600

IN2’^{h}

C	2.13075700	0.19134500	0.02390200
C	2.70097100	-1.16845700	-0.38707000
H	3.73951000	-1.25612500	-0.05673700
H	2.11731700	-1.97566400	0.04928600
H	2.68046200	-1.26951300	-1.47511900
C	2.10900600	0.35167300	1.54599300
H	3.12794400	0.32542400	1.94138700
H	1.66431500	1.31326000	1.81441600
H	1.52591200	-0.44223400	2.00648100
C	2.96991800	1.30783400	-0.59720700
H	2.97213200	1.22374300	-1.68587900

H	2.56353500	2.28560300	-0.33052000
H	4.00189300	1.25333300	-0.24329200
O	0.81948500	0.40643100	-0.54711900
C	-0.27205400	-0.41519500	-0.21695500
O	-0.11472800	-1.36708800	0.57446500
N	-1.35130900	-0.01396300	-0.86050300
C	-2.56018500	-0.75910200	-0.54839000
H	-3.17567600	-0.82348900	-1.45500400
C	-3.36249400	-0.01625200	0.52247600
H	-2.87456100	-0.14424300	1.49851500
H	-4.38532100	-0.39749600	0.60011200
O	-3.43635300	1.37487700	0.19220700
H	-2.57918600	1.52036800	-0.25479700
H	-2.36185800	-1.78005900	-0.19824100

IN3[h]

C	-4.16731400	0.51738700	-0.06826000
C	-4.87762800	0.06260100	1.20363800
H	-5.93303900	0.33780400	1.14775300
H	-4.80308200	-1.01447300	1.33456100
H	-4.44349700	0.55502300	2.07645300
C	-4.68266200	-0.20882800	-1.30755200
H	-5.73027000	0.05239200	-1.47175200
H	-4.11400700	0.09575400	-2.18872700
H	-4.60536600	-1.28792300	-1.19485500
C	-4.30146800	2.02598300	-0.24170200
H	-3.89668100	2.55053300	0.62550500
H	-3.76388400	2.36040600	-1.13055200
H	-5.35319300	2.29615500	-0.35037200
O	-2.71586200	0.33470900	0.06326800
C	-2.16321800	-0.89669800	0.22887100
O	-2.78828200	-1.94441400	0.31283600
N	-0.82184100	-0.79089100	0.27120900
C	0.07351100	-1.89849300	0.55100300
H	0.13070400	-2.08048300	1.63100400
C	1.45542500	-1.54343900	0.01551400
H	1.41236100	-1.53981300	-1.08589700
H	2.14774700	-2.35048700	0.30499800
O	1.83389000	-0.30593400	0.52496400
H	-0.30727400	-2.80248800	0.07456900
H	-0.39008000	0.12281700	0.29906300
B	3.04228700	0.42846200	-0.06129100
C	3.20583800	1.75523800	0.89772500
C	2.73265900	0.87565300	-1.62182500
C	4.41430700	-0.50244900	0.02404000
H	3.96371500	2.43097800	0.47141000
H	2.26677700	2.32725700	0.88547500
C	3.56754000	1.46968100	2.36076500
H	2.63739400	-0.00955800	-2.26670000
H	4.41215000	-1.05084500	0.97788300
H	2.83995100	0.78592100	2.80787600
H	4.54915200	0.99287400	2.44700000
H	3.59614200	2.37234200	2.98468000
H	3.60683500	1.41460500	-2.01946800

C	1.48470200	1.74252200	-1.82984900
H	0.59053300	1.23627000	-1.45346800
H	1.55839600	2.69314600	-1.29329000
H	1.29699100	1.98387400	-2.88395400
C	4.69729600	-1.50269600	-1.10807500
H	3.87741200	-2.21283500	-1.24608900
H	4.83270800	-0.99207900	-2.06623600
H	5.60528400	-2.09443800	-0.93356900
H	5.28567600	0.16755100	0.09038600

IN3'[h]

C	2.87324000	-0.34744200	-0.21243900
C	3.41825000	-0.35857900	1.21488300
H	4.47773000	-0.62505400	1.20243700
H	3.30640700	0.61677000	1.68334200
H	2.88701300	-1.10045500	1.81491400
C	3.55400500	0.71378800	-1.07602200
H	4.61398700	0.47254800	-1.18748800
H	3.10327500	0.73344800	-2.07099400
H	3.45773100	1.70026200	-0.62978300
C	3.04501200	-1.72450800	-0.84912000
H	2.54654100	-2.48920600	-0.25172300
H	2.61508800	-1.73785500	-1.85211900
H	4.10498100	-1.97577100	-0.92219000
O	1.42803100	-0.17178800	-0.20943300
C	0.83806500	0.92920900	0.34271500
O	1.49912900	1.90565200	0.71453400
N	-0.50311300	0.81240400	0.40725800
C	-1.15667000	2.03911400	0.86899000
H	-2.14550100	1.79008200	1.23938300
C	-1.28056100	3.07334000	-0.24527700
H	-0.28173900	3.40990900	-0.54250900
H	-1.84085700	3.94190900	0.11062700
O	-1.98860400	2.57033800	-1.38452600
H	-1.65964900	1.67269700	-1.53432100
H	-0.59071500	2.48909500	1.68874800
B	-1.33461700	-0.56202900	0.07922000
C	-0.70894800	-1.79330600	0.97758200
C	-1.22754300	-0.83734100	-1.55624700
C	-2.91312400	-0.32342900	0.49971000
H	-1.35918200	-2.67328500	0.87954700
H	0.26011000	-2.10396600	0.57942900
C	-0.53824900	-1.48816600	2.47059900
H	-0.32283200	-0.35267000	-1.93962300
H	-2.06881600	-0.34401800	-2.07228900
C	-1.18946000	-2.29934800	-2.02440800
H	-3.01045300	-0.09839900	1.57186700
H	-3.31492500	0.55819700	-0.01637300
C	-3.84754600	-1.49918300	0.18470100
H	0.13121500	-0.63627400	2.62486200
H	-1.49185600	-1.22797500	2.94187700
H	-0.12260600	-2.33170600	3.03599100
H	-2.06272000	-2.86435300	-1.68769300
H	-1.14959900	-2.39097600	-3.11711200

H	-0.30893200	-2.81294000	-1.62874900
H	-4.88060400	-1.30508600	0.49733800
H	-3.87538100	-1.71131600	-0.88766800
H	-3.53011000	-2.41881600	0.68475700

IN1^{Me}

C	2.60050000	0.14519300	-0.09991000
C	2.49408700	0.32101100	-1.61115100
H	3.49256800	0.27309600	-2.05024800
H	1.87939100	-0.45630200	-2.05949200
H	2.06411500	1.29575500	-1.85122600
C	3.11721800	-1.23629100	0.28772000
H	4.14480300	-1.34749500	-0.06419600
H	3.11570700	-1.35169100	1.37361100
H	2.51151800	-2.02547100	-0.15181700
C	3.47740600	1.23390500	0.50571100
H	3.08532000	2.22357800	0.26514900
H	3.52399500	1.13218600	1.59128600
H	4.49031100	1.15718400	0.10760200
O	1.29137500	0.39552600	0.53302700
C	0.21429200	-0.37670400	0.26280700
O	0.18767900	-1.32431000	-0.50371300
N	-0.84839200	0.05279200	0.98955000
H	-0.72995800	0.88310700	1.54766400
C	-2.17335900	-0.51449200	0.83985200
H	-2.73028400	-0.32131000	1.75818000
C	-2.94809300	0.05880100	-0.34760000
H	-2.38659500	-0.16309100	-1.26246900
C	-3.17082000	1.55830000	-0.24279500
H	-2.21843500	2.09284200	-0.23743500
H	-3.74698900	1.92254800	-1.09749600
H	-3.71703700	1.80403800	0.67181800
O	-4.19187400	-0.65949000	-0.36100900
H	-4.71959200	-0.32748600	-1.09770500
H	-2.08491100	-1.59317000	0.71567100

IN2^{Me}

C	-2.54512300	0.18974600	0.10132300
C	-2.48052200	0.14329700	1.62558800
H	-3.49407200	0.11142400	2.03133600
H	-1.93683800	-0.73260100	1.97180300
H	-1.98720900	1.03817900	2.01096400
C	-3.15005600	-1.08172400	-0.48797200
H	-4.19242900	-1.16758700	-0.17345600
H	-3.12621100	-1.04229300	-1.57925800
H	-2.61092200	-1.96626700	-0.15661200
C	-3.32967900	1.41327100	-0.35931100
H	-2.87821500	2.32822200	0.02837100
H	-3.34886700	1.47085100	-1.44913100
H	-4.35770800	1.35468000	0.00222900
O	-1.21019400	0.42972800	-0.45957500
C	-0.19082500	-0.45925500	-0.27859000
O	-0.27418100	-1.49649800	0.35994800
N	0.90665700	-0.02822800	-0.93847800

C	2.23991200	-0.59383300	-0.72976200
H	2.79707300	-0.50519800	-1.66748700
C	3.09069100	0.05262800	0.40489200
H	2.41698700	-0.00126000	1.31315200
C	3.24380800	1.57092400	0.11782000
H	2.28801600	2.10763900	0.04541300
H	3.82538700	2.02997500	0.92234200
H	3.79862900	1.71317900	-0.81757700
O	4.26180600	-0.58295800	0.54436800
H	2.12345700	-1.65158000	-0.50140300
H	0.84763100	0.89773300	-1.33319500

IN3^{Me}

C	4.88996100	-0.10396900	0.30235400
C	4.80301200	1.25961000	0.98129800
H	5.81078300	1.62760400	1.18510200
H	4.28728100	1.98243100	0.35337300
H	4.27237800	1.17647300	1.93213600
C	5.55229900	-0.02434800	-1.06991500
H	6.59343400	0.28312600	-0.95137800
H	5.53999500	-1.00291700	-1.55472400
H	5.04769600	0.69318700	-1.71285300
C	5.62775700	-1.09694300	1.19279100
H	5.13285600	-1.18727900	2.16129000
H	5.66012300	-2.08295900	0.72592600
H	6.65192700	-0.75853500	1.35790900
O	3.55118600	-0.70254400	0.19059300
C	2.56941300	-0.11775100	-0.54559500
O	2.67876900	0.93177700	-1.15753400
N	1.45966500	-0.89096800	-0.50704400
H	1.49137800	-1.70153800	0.09238000
C	0.16985000	-0.48776200	-1.04396800
H	-0.25145700	-1.31531600	-1.61794100
H	0.33557500	0.34655400	-1.72403600
C	-0.85022600	-0.08584400	0.03392300
H	-0.90811900	-0.92809400	0.74368500
O	-2.06282400	0.10620700	-0.62113300
B	-3.42054400	0.08016600	0.08884800
C	-4.47646800	-0.39799000	-1.08353500
C	-3.82509600	1.59611200	0.62257700
C	-3.40643800	-0.97570700	1.35804800
H	-5.51611800	-0.27041000	-0.74614600
H	-4.36764700	0.27750200	-1.94400000
C	-4.29225200	-1.83515300	-1.58330300
H	-3.20555600	1.87338500	1.48868900
H	-4.85642800	1.58667700	1.00684900
C	-3.71329500	2.70995100	-0.42546200
H	-3.01143800	-1.95109500	1.03624100
H	-2.70804600	-0.63059200	2.13556100
C	-4.76904400	-1.20845300	2.02486300
H	-3.25894500	-1.99600400	-1.90545500
H	-4.49805600	-2.56599900	-0.79418800
H	-4.94261300	-2.08928800	-2.43039700
H	-4.40819900	2.54622800	-1.25558800

H	-3.92585700	3.70916500	-0.02371200
H	-2.70859600	2.73434500	-0.85626100
H	-4.72323300	-1.91963300	2.85931600
H	-5.18281300	-0.27704500	2.42334900
H	-5.50263800	-1.59871100	1.31266600
C	-0.37023000	1.14097600	0.81553800
H	0.61415500	0.96890900	1.26053100
H	-0.30342700	2.00787000	0.15343200
H	-1.06583100	1.37802000	1.62022800

IN2’^{Me}

C	-2.56238600	0.12188800	0.09835600
C	-2.46338300	0.18004000	1.62530800
H	-3.44668100	0.01393300	2.07386000
H	-1.76931700	-0.57307100	1.99076300
H	-2.10811700	1.16496400	1.93912800
C	-3.01758500	-1.25999900	-0.37847600
H	-4.01982100	-1.47881900	0.00001100
H	-3.05291300	-1.28563000	-1.47065700
H	-2.32889800	-2.02813900	-0.03421100
C	-3.54899700	1.18253700	-0.39063800
H	-3.22645700	2.17775500	-0.07751600
H	-3.61256200	1.17151700	-1.48069400
H	-4.54554900	0.99731800	0.01675500
O	-1.32014600	0.51058700	-0.52686300
C	-0.13555400	-0.23526700	-0.34151000
O	-0.14750700	-1.22946300	0.41715000
N	0.84201400	0.28009600	-1.05299800
C	2.10768600	-0.41393300	-0.90500800
H	2.72647200	-0.21863500	-1.79044100
C	2.90726900	0.03778200	0.31933300
H	2.29209200	-0.14816800	1.20701700
C	3.29120400	1.50683600	0.26748400
H	2.39631000	2.12733400	0.19757900
H	3.84346600	1.80354100	1.16529200
H	3.91939200	1.70633900	-0.60576700
O	4.08761100	-0.79620400	0.38111700
H	4.62467800	-0.48689300	1.12059300
H	1.98462400	-1.50267800	-0.82103900

IN3’^{Me}

C	3.09354100	-0.00158100	0.10529300
C	3.58187900	-1.28063500	0.78188400
H	4.66941000	-1.26385300	0.87742900
H	3.14743500	-1.37806300	1.77814000
H	3.29815200	-2.15675900	0.19657700
C	3.45267500	1.21043800	0.96441300
H	4.53417700	1.24758700	1.11626600
H	3.12834900	2.13342400	0.49018200
H	2.97407000	1.13104200	1.94331000
C	3.68459300	0.10106700	-1.30038400
H	3.36939600	-0.75285600	-1.90413000
H	3.36297100	1.01530500	-1.79295800
H	4.77576900	0.09242500	-1.24202000

O	1.65257300	-0.19325600	0.05429200
C	0.82139900	0.74317700	-0.49704600
O	1.26260500	1.80137100	-0.96094100
N	-0.47077300	0.36659500	-0.45485000
C	-1.38375200	1.35771300	-1.01879600
H	-2.19286000	0.84562700	-1.53457000
H	-0.86107300	1.97982200	-1.74615000
C	-2.01402500	2.27688800	0.03031700
H	-2.48508300	1.65276800	0.79684700
O	-3.04050000	3.01495000	-0.66821200
H	-3.41460800	3.65297800	-0.04878000
B	-1.01477800	-1.14683600	-0.10438200
C	-0.50521800	-1.61490400	1.38634800
C	-0.40705800	-2.11282100	-1.29668400
C	-2.67715100	-1.13007000	-0.15575100
H	-0.99503500	-2.56259700	1.64625800
H	0.56573200	-1.83345500	1.36959700
C	-0.77453500	-0.61005800	2.51169200
H	0.68652300	-2.03587900	-1.27735700
H	-0.71125800	-1.69966400	-2.27172500
C	-0.78002300	-3.60140000	-1.27571300
H	-3.08371300	-0.19511800	0.24476900
H	-3.01667600	-1.15267800	-1.20210700
C	-3.39913600	-2.26271300	0.59250500
H	-0.23014900	0.32217500	2.33668000
H	-1.83630400	-0.34801500	2.57439900
H	-0.47652700	-0.97886400	3.50163300
H	-1.85028600	-3.75633600	-1.43808900
H	-0.25728500	-4.17722200	-2.05004900
H	-0.53257900	-4.06414700	-0.31462300
H	-4.48299600	-2.23905300	0.42449500
H	-3.05050200	-3.25284800	0.29220700
H	-3.24458700	-2.18965400	1.67248600
C	-1.03514700	3.22068400	0.70771400
H	-0.25504600	2.66086100	1.22432700
H	-0.55827700	3.87526700	-0.02336600
H	-1.55351900	3.83656700	1.45031500

$IN1^{Ph}$

C	-3.95180500	0.00660800	-0.08989000
C	-4.20469200	-0.72940100	1.22084800
H	-5.24497600	-1.05910300	1.25038700
H	-3.56213500	-1.60131300	1.32047700
H	-4.03501600	-0.06420600	2.06995500
C	-4.08278300	-0.90420600	-1.30517100
H	-5.11787600	-1.24089900	-1.38903900
H	-3.82725800	-0.36125400	-2.21736500
H	-3.43997600	-1.77763000	-1.22197700
C	-4.87495700	1.21100300	-0.21790700
H	-4.75069000	1.88509600	0.63120500
H	-4.66483100	1.76280200	-1.13561000
H	-5.91314600	0.87722600	-0.24538100
O	-2.61033000	0.63131700	-0.06801000
C	-1.48753200	-0.10624500	0.03792200

O	-1.42965400	-1.31692100	0.12075800
N	-0.41425000	0.74584600	0.03682600
C	0.95021900	0.43480200	0.11783200
C	1.84898900	1.50987400	0.10643500
C	1.45016900	-0.86750200	0.21324100
C	3.21331400	1.28577900	0.18526700
H	1.46721400	2.52234900	0.03815700
C	2.82318200	-1.07044600	0.29057300
H	0.77094600	-1.70393800	0.22770000
C	3.72585600	-0.00954000	0.27911400
H	3.89124500	2.13145600	0.17520600
H	3.19686700	-2.08553600	0.36303700
H	-0.64118700	1.72733900	-0.03676000
C	5.20422000	-0.25153300	0.32326400
H	5.41377000	-1.18305700	0.85912300
H	5.70233000	0.56805200	0.85195600
O	5.70398900	-0.33802400	-1.02699800
H	6.65735600	-0.48444200	-0.97401700

IN2^{Ph}

C	-3.90445400	0.01664100	-0.06656500
C	-4.13750900	-0.62640900	1.29667500
H	-5.17789300	-0.94989200	1.36834200
H	-3.49405700	-1.49063600	1.44520800
H	-3.95023400	0.09541200	2.09441500
C	-4.06701500	-0.97661400	-1.21244000
H	-5.10520200	-1.31251400	-1.25183500
H	-3.82745400	-0.50015000	-2.16531900
H	-3.42525100	-1.84482400	-1.08166900
C	-4.82880900	1.21229300	-0.25949700
H	-4.68486600	1.94388200	0.53740800
H	-4.63616900	1.69817000	-1.21750700
H	-5.86853500	0.88206700	-0.24217200
O	-2.56539600	0.63384700	-0.11363500
C	-1.43941100	-0.10661100	0.02172200
O	-1.39767600	-1.31212300	0.17965900
N	-0.36514500	0.72953900	-0.05059100
C	1.00647600	0.41545800	0.05560800
C	1.90168700	1.49021900	0.11414900
C	1.50929200	-0.88684600	0.10235000
C	3.26753900	1.26296100	0.21173700
H	1.51625800	2.50423200	0.08362400
C	2.88465300	-1.08695800	0.20385800
H	0.83183600	-1.72516000	0.06457500
C	3.79251900	-0.03062100	0.26880600
H	3.94383400	2.11074800	0.25272700
H	3.26074400	-2.10468400	0.23767600
H	-0.58536500	1.70890900	-0.15903100
C	5.30461600	-0.27251000	0.25743600
H	5.46194800	-1.21707100	0.85732800
H	5.74240900	0.53334100	0.91898400
O	5.81513600	-0.30980300	-0.98498000

IN3^{Ph}

B	-4.39555300	0.50955200	-0.02075500
C	-5.58135300	-0.48607400	-0.59491600
C	-3.52738800	1.16452300	-1.27056600
C	-5.02089900	1.72882600	0.89310500
H	-6.21217200	0.07081200	-1.30383400
H	-5.14935200	-1.29923800	-1.19551000
C	-6.47743700	-1.11447400	0.47868800
H	-2.68925700	1.74125500	-0.85124100
H	-4.16108400	1.91258800	-1.77114500
C	-2.97375200	0.23450600	-2.36084400
H	-5.39829000	1.30777500	1.83586400
H	-4.20131400	2.40194100	1.19049500
C	-6.13234600	2.56902600	0.25375300
H	-5.87797300	-1.67240100	1.20472400
H	-7.02178600	-0.35158700	1.04411100
H	-7.22560500	-1.80700700	0.07185100
H	-3.77215100	-0.34381100	-2.83664100
H	-2.45728600	0.77980600	-3.16137800
H	-2.25563300	-0.48295000	-1.95699000
H	-6.47855200	3.38693500	0.89808500
H	-5.80301700	3.02340400	-0.68645400
H	-7.00951600	1.95825800	0.01705100
C	6.11955700	0.51593500	-0.05852900
C	6.32325400	0.82456400	1.42099000
H	7.27521300	1.34297900	1.55169900
H	5.52790400	1.45617500	1.81014100
H	6.35717400	-0.09981400	2.00131100
C	5.97318200	1.77866800	-0.90086500
H	6.91093900	2.33729500	-0.87548000
H	5.75948900	1.52019800	-1.94003700
H	5.17759100	2.41849100	-0.52609600
C	7.25476100	-0.35212800	-0.58636700
H	7.33371900	-1.27596500	-0.01081000
H	7.09016200	-0.60733400	-1.63457200
H	8.19977000	0.18717300	-0.50606200
O	4.93988700	-0.35372200	-0.23688700
C	3.69985300	0.05459100	0.11824200
O	3.41607700	1.13513400	0.59790400
N	2.82301300	-0.95523600	-0.15487500
C	1.43184200	-0.99835700	0.06163500
C	0.78338300	-2.20576800	-0.22940200
C	0.67637200	0.07660500	0.53234900
C	-0.58514800	-2.32869800	-0.05198700
H	1.36280400	-3.04454500	-0.60018300
C	-0.69860500	-0.06935000	0.70675500
H	1.15963500	1.01341400	0.75913300
C	-1.35279100	-1.26096600	0.41940700
H	-1.06612500	-3.27188800	-0.29123700
H	-1.29040800	0.76271300	1.06324000
H	3.23490000	-1.79235700	-0.54063700
C	-2.85370700	-1.41349100	0.61169500
H	-3.00262700	-2.17707100	1.39544500
H	-3.26420900	-1.85607400	-0.30708200
O	-3.50457800	-0.24489900	0.97414400

IN2’^{Ph}

C	3.90915900	-0.00629800	0.09473000
C	4.15335700	-0.70560400	-1.24392100
H	5.17134300	-1.10217100	-1.27615700
H	3.44980000	-1.52184700	-1.38959100
H	4.03930200	0.00583400	-2.06544100
C	4.01012300	-0.98349200	1.26769100
H	5.02509500	-1.38415400	1.33065500
H	3.78926600	-0.46804200	2.20558400
H	3.30988100	-1.80674700	1.14887100
C	4.92093600	1.12388600	0.27815300
H	4.84147600	1.84484500	-0.53782900
H	4.74182700	1.64914400	1.21842100
H	5.93794400	0.72620000	0.29166500
O	2.63625600	0.69025700	0.09700600
C	1.42961700	0.00833000	-0.04516500
O	1.42426300	-1.22602900	-0.16624500
N	0.43114700	0.89087200	-0.02294100
C	-0.88404300	0.49802700	-0.11195800
C	-1.84971100	1.53781100	-0.10095200
C	-1.39825700	-0.82148600	-0.21265900
C	-3.20711800	1.28739700	-0.17983400
H	-1.48581100	2.55651000	-0.03115800
C	-2.76598800	-1.05506600	-0.28867900
H	-0.70316700	-1.64537200	-0.23293400
C	-3.70120800	-0.01941100	-0.27505200
H	-3.90380000	2.11999200	-0.16856700
H	-3.11788800	-2.08004300	-0.36334200
C	-5.16974400	-0.29219000	-0.32293300
H	-5.35997200	-1.23442200	-0.84880100
H	-5.68632300	0.50977500	-0.86252000
O	-5.69804600	-0.37788600	1.02370700
H	-6.64952300	-0.52776700	0.94946000

IN3’^{Ph}

B	-0.78706400	1.71150100	-0.01669800
C	-1.65439600	2.03177600	1.34267700
C	-1.74184300	1.94130000	-1.33671900
C	0.55039900	2.67375500	-0.09383800
H	-1.98619400	3.07942600	1.30103900
H	-2.57339800	1.43629100	1.33081200
C	-0.94633400	1.80148500	2.68189700
H	-2.00654900	3.00736300	-1.38979100
H	-2.69049000	1.41335800	-1.19491100
C	-1.15557000	1.52193700	-2.68862600
H	1.13098200	2.45776800	-0.99997700
C	0.25821900	4.17968500	-0.06964100
H	-0.67781700	0.74778700	2.80484500
H	-0.01765900	2.37681900	2.75610900
H	-1.56411200	2.07588700	3.54619000
H	-0.94300900	0.44872200	-2.70689300
H	-1.82825300	1.72914600	-3.53035700
H	-0.21247000	2.03685500	-2.89947300

H	1.17063000	4.78327000	-0.14554200
H	-0.39225400	4.47963700	-0.89680800
H	-0.24625400	4.47848700	0.85399600
C	-3.36868200	-1.70852900	0.02583700
C	-3.28701600	-2.60930800	1.25723500
H	-4.14518100	-3.28545300	1.27595700
H	-2.37219900	-3.19672300	1.25115800
H	-3.31014000	-2.00444200	2.16666100
C	-3.26316900	-2.50451100	-1.27405500
H	-4.11894300	-3.17781200	-1.36367100
H	-3.27173900	-1.82672900	-2.13070500
H	-2.34771800	-3.09083100	-1.30055400
C	-4.67167900	-0.91315800	0.04640700
H	-4.74042600	-0.31257000	0.95490500
H	-4.72595700	-0.24274300	-0.81278100
H	-5.52641700	-1.59155400	0.01297700
O	-2.35326900	-0.66534400	0.07675900
C	-1.02146500	-0.95974300	0.04381600
O	-0.60259600	-2.12036700	0.04078900
N	-0.26178200	0.15350700	0.01859100
C	1.14063800	-0.11829000	0.00945400
C	1.83995900	-0.21404200	-1.19283300
C	1.84896100	-0.24795000	1.20350600
C	3.21436100	-0.41866600	-1.20005300
H	1.29770600	-0.11538200	-2.12376400
C	3.22294700	-0.45264000	1.19531000
H	1.31366400	-0.17287800	2.14076300
C	3.92725800	-0.53619700	-0.00657400
H	3.74258500	-0.48227800	-2.14523300
H	3.75814900	-0.54213700	2.13445700
C	5.40814400	-0.77184500	-0.01483300
H	5.86389600	-0.31482500	0.87010200
H	5.85390100	-0.31409000	-0.90440600
O	5.66421400	-2.19228600	-0.01642900
H	6.62254700	-2.31223800	-0.01548400
H	1.23972900	2.45344300	0.73099000

S7. Reference

(1) Hermanek, S. Boron-11 NMR spectra of boranes, main-group heteroboranes, and substituted derivatives. Factors influencing chemical shifts of skeletal atoms. *Chem. Rev.* **1992**, *92* (2), 325-362. DOI: 10.1021/cr00010a007

(2) Frisch, M. J.; Trucks, G. W.; Schlegel, H. B.; Scuseria, G. E.; Robb, M. A.; Cheeseman, J. R.; Scalmani, G.; Barone, V.; Petersson, G. A.; Nakatsuji, H.; X. Li, M. C.; Marenich, A. V.; Bloino, J.; Janesko, B. G.; Gomperts, R.; Mennucci, B.; Hratchian, H. P.; Ortiz, J. V.; Izmaylov, A. F.; Sonnenberg, J. L.; Williams-Young, D.; Ding, F.; Lipparini, F.; Egidi, F.; Goings, J.; Peng, B.; Petrone, A.; Henderson, T.; Ranasinghe, D.; Zakrzewski, V. G.; Gao, J.; Rega, N.; Zheng, G.; Liang, W.; Hada, M.; Ehara, M.; Toyota, K.; Fukuda, R.; Hasegawa, J.; Ishida, M.; Nakajima, T.; Honda, Y.; Kitao, O.; Nakai, H.; Vreven, T.; Throssell, K.; Jr., J. A. M.; Peralta, J. E.; Ogliaro, F.; Bearpark, M. J.; Heyd, J. J.; Brothers, E. N.; Kudin, K. N.; Staroverov, V. N.; Keith, T. A.; Kobayashi, R.; Normand, J.; Raghavachari, K.; Rendell, A. P.; Burant, J. C.; Iyengar, S. S.; Tomasi, J.; Cossi, M.; Millam, J. M.; Klene, M.; Adamo, C.; Cammi, R.; Ochterski, J. W.; Martin, R. L.; Morokuma, K.; Farkas, O.; Foresman, J. B.; Fox, D. J., Gaussian 16 Rev. C.01 **2016**.

(3) Stephens, P. J.; Devlin, F. J.; Chabalowski, C. F.; Frisch, M. J. Ab Initio Calculation of Vibrational Absorption and Circular Dichroism Spectra Using Density Functional Force Fields. *J. Phys. Chem.* **1994**, *98* (45), 11623–11627. DOI: 10.1021/j100096a001

(4) Grimme, S.; Antony, J.; Ehrlich, S.; Krieg, H. A Consistent and Accurate *Ab Initio* Parametrization of Density Functional Dispersion Correction (DFT-D) for the 94 Elements H-Pu. *J. Chem. Phys.* **2010**, *132* (15), 154104. DOI: 10.1063/1.3382344

(5) Grimme, S.; Ehrlich, S.; Goerigk, L. Effect of the Damping Function in Dispersion Corrected Density Functional Theory. *J. Comput. Chem.* **2011**, *32* (7), 1456–1465. DOI: 10.1002/jcc.21759

(6) Weigend, F.; Ahlrichs, R. Balanced Basis Sets of Split Valence, Triple Zeta Valence and Quadruple Zeta Valence Quality for H to Rn: Design and Assessment of Accuracy. *Phys. Chem. Chem. Phys.* **2005**, *7* (18), 3297. DOI: doi.org/10.1039/b508541a

(7) Marenich, A. V.; Cramer, C. J.; Truhlar, D. G. Universal Solvation Model Based on Solute Electron Density and on a Continuum Model of the Solvent Defined by the Bulk Dielectric Constant and Atomic Surface Tensions. *J. Phys. Chem. B* **2009**, *113* (18), 6378–6396. DOI: 10.1021/jp810292n

(8) Grimme, S. Semiempirical Hybrid Density Functional with Perturbative Second-Order Correlation. *J. Chem. Phys.* **2006**, *124* (3), 034108. DOI: 10.1063/1.2148954

(9) Zheng, J.; Xu, X.; Truhlar, D. G. Minimally Augmented Karlsruhe Basis Sets. *Theor. Chem. Acc.* **2011**, *128* (3), 295–305. DOI: 10.1007/s00214-010-0846-z

(10) Zhao, Y.; Schultz, N. E.; Truhlar, D. G. Design of Density Functionals by Combining the Method of Constraint Satisfaction with Parametrization for Thermochemistry, Thermochemical Kinetics, and Noncovalent Interactions. *J. Chem. Theory Comput.* **2006**, *2* (2), 364–382. DOI: 10.1021/ct0502763

(11) Hehre, W. J.; Ditchfield, R.; Pople, J. A. Self—Consistent Molecular Orbital Methods. XII. Further Extensions of Gaussian—Type Basis Sets for Use in Molecular Orbital Studies of Organic Molecules. *J. Chem. Phys.* **1972**, *56* (5), 2257–2261. DOI: 10.1063/1.1677527

(12) Hariharan, P. C.; Pople, J. A. The Influence of Polarization Functions on Molecular Orbital Hydrogenation Energies. *Theor. Chim. Acta* **1973**, *28* (3), 213–222. DOI: 10.1007/BF00533485

(13) Dill, J. D.; Pople, J. A. Self-Consistent Molecular Orbital Methods. XV. Extended Gaussian-Type Basis Sets for Lithium, Beryllium, and Boron. *J. Chem. Phys.* **1975**, *62* (7), 2921–2923. DOI: 10.1063/1.430801

(14) Fukui, K. The Path of Chemical Reactions - the IRC Approach. *Acc. Chem. Res.* **1981**, *14* (12), 363–368. DOI: 10.1021/ar00072a001

(15) Lu, T.; Chen, F. Multiwfn: A Multifunctional Wavefunction Analyzer. *J. Comput. Chem.* **2012**, *33* (5), 580–592. DOI: 10.1002/jcc.22885

(16) CYLview20; Legault, C. Y., Université de Sherbrooke, 2020. http://www.cylview.org (accessed 2024-03-16).

(17) Humphrey, W.; Dalke, A.; Schulten, K. VMD: Visual Molecular Dynamics. *J. Mol. Graph.* **1996**, *14* (1), 33–38. DOI: 10.1016/0263-7855(96)00018-5
